# A high-throughput and open-source platform for embryo phenomics

**DOI:** 10.1371/journal.pbio.3000074

**Published:** 2018-12-13

**Authors:** Oliver Tills, John I. Spicer, Andrew Grimmer, Simone Marini, Vun Wen Jie, Ellen Tully, Simon D. Rundle

**Affiliations:** 1 Marine Biology and Ecology Research Centre, School of Biological and Marine Sciences, University of Plymouth, Plymouth, Devon, United Kingdom; 2 Istituto di Scienze Marine, Consiglio Nazionale delle Ricerche, Sede Secondaria di Lerici, Forte Santa Teresa, Lerici (La Spezia), Italy; University of Sussex, UNITED KINGDOM

## Abstract

Phenomics has the potential to facilitate significant advances in biology but requires the development of high-throughput technologies capable of generating and analysing high-dimensional data. There are significant challenges associated with building such technologies, not least those required for investigating dynamic processes such as embryonic development, during which high rates of temporal, spatial, and functional change are inherently difficult to capture. Here, we present EmbryoPhenomics, an accessible high-throughput platform for phenomics in aquatic embryos comprising an Open-source Video Microscope (OpenVIM) that produces high-resolution videos of multiple embryos under tightly controlled environmental conditions. These videos are then analysed by the Python package Embryo Computer Vision (EmbryoCV), which extracts phenomic data for morphological, physiological, behavioural, and proxy traits during the process of embryonic development. We demonstrate the broad-scale applicability of EmbryoPhenomics in a series of experiments assessing chronic, acute, and multistressor responses to environmental change (temperature and salinity) in >30 million images of >600 embryos of two species with markedly different patterns of development—the pond snail *Radix balthica* and the marine amphipod *Orchestia gammarellus*. The challenge of phenomics is significant but so too are the rewards, and it is particularly relevant to the urgent task of assessing complex organismal responses to current rates of environmental change. EmbryoPhenomics can acquire and process data capturing functional, temporal, and spatial responses in the earliest, most dynamic life stages and is potentially game changing for those interested in studying development and phenomics more widely.

## Abstract 2

EmbryoPhenomics is an open-source technology platform for high-throughput phenome screening of aquatic embryos. This paper demonstrates its application in experiments assessing the sensitivity of aquatic embryos to environmental stress, consisting of more than 600 embryos and more than 30 million images.

## Author summary

Phenomics is the collection of high-dimensional phenotypic data on an organism-wide scale, and it requires high-throughput technologies. However, a lack of technologies for efficiently visualising and measuring whole-organism responses to different environments represents a serious challenge for biologists. This challenge is most apparent when studying complex responses, such as those occurring during the dynamic period of embryonic development, when the phenotype changes markedly through time. Here, we present EmbryoPhenomics (www.embryophenomics.org), a new open-source technological platform comprising high-throughput bioimaging hardware that produces high-resolution video of multiple, developing embryos maintained under controlled environmental conditions and software for automatically quantifying embryo responses from these videos. We demonstrate the broad applicability of EmbryoPhenomics using four experiments assessing responses to global change (elevated temperature and salinity) in which we generate data for more than 600 embryos produced from video comprising more than 30 million images. EmbryoPhenomics was used to capture functional, temporal, and spatial change in morphological, physiological, and behavioural responses in the earliest, most dynamic life stages and addresses a serious bottleneck in biology. Such capabilities are urgently required, particularly within the context of assessing the response of embryos to the current unprecedented rates of global environmental change.

## Introduction

Phenomics is the acquisition of high-dimensional phenotypic data on an organism-wide scale [1]. It is both analogous and complementary to genomics and has a similar capacity to facilitate advances in biology. However, it is now recognised that in many ways, the challenges of phenomics dwarf those faced at the advent of modern genomics [2,3]. The information content of phenomes is far greater than genomes [1,4], and whereas genomics has benefited from standardised tools and technologies that are readily applicable to different species and scales of study, phenomics requires the development and application of less generic approaches [5]. Consequently, a lack of transferrable technologies for high-dimensional acquisition of phenome-level data remains the greatest bottleneck to the advancement of phenomics and is recognised as a key challenge in biology [3,5].

Technologies for acquiring high-resolution phenomic data enable scales of data collection [6] and combinatorial analyses that would never be possible using manual approaches [7]. Such analyses permit the relationships and interactions among multiple traits to be explored and integrated and, for phenomic data, have been demonstrated to have greater discriminatory and explanatory power than univariate approaches, thereby providing novel biological insight [8,9]. High-dimensional phenotyping also allows for the identification and measurement of ‘proxy’ traits—phenome-level measures that are not detectable using manual observation [4]. In plant root phenomics, such proxy traits have been shown to possess strong discriminatory power and include automated traits with natural manual trait equivalents and novel measurements for which there was no manual trait equivalent [9]. Another key asset of data sets acquired using high-throughput phenomic technologies is the ability to incorporate biological complexity in the interrogation of a particular experimental response. For example, analysis of phenomic data for mice identified a combinatorial signal from >200 phenotypic traits that was predictive of a Huntington disease genotype [8]. Such studies point to the power of phenomics to transform our understanding of phenome-level response via the development and application of appropriate technologies.

The rewards of phenomics will arguably be greatest when used to quantify aspects of the phenome for which current methods are most limiting. Embryonic development is a dynamic process with high levels of intraindividual temporal, spatial, and functional change, meaning that traditional approaches to its quantification at the whole-organism level can never be thorough. Furthermore, in addition to high levels of intraindividual change, embryonic development also exhibits high levels of interindividual variation [10], and therefore, an optimum approach to its study is to adopt longitudinal observation of large numbers of embryos [11]. This task is difficult to achieve and necessitates compromises in i) the number of embryos studied, ii) the number of phenomic measures quantified, iii) the precision with which measurements are made, and iv) the temporal resolution of measurements. Such compromises occur due to the highly dynamic nature of biological development but also because of the complexity and interconnectedness of biological responses through time. Therefore, automated technologies with the capability to make high-throughput longitudinal measurements of developing embryos offer huge potential to alleviate a major limitation in the application of phenomics.

There are good examples of when automated longitudinal observation of developing embryos has been used effectively for commercial applications. Automated noninvasive imaging and analysis of cytokinetic patterns in early-stage cell cleavages in human embryos can predict survival to the blastocyst stage with >93% sensitivity [12]. Consequently, time-lapse imaging is now routinely offered as an option in in vitro fertilisation (IVF) treatments to select embryos for implantation on the basis of early cytokinetic parameters, and this approach significantly increases success rates [13]. Technologies used for nonhuman, high-throughput screening include the ImageXpress system for widefield cellular imaging [14, 15] and the EthoVision platform [16] for use with zebrafish embryos and larvae, with a focus on tracking [17]. A limitation of commercial platforms remains their transferability and applicability to different species, research questions, and study designs. Consequently, some laboratories are turning to self-built, open-access solutions, comprising open-source hardware [18, 19] and software [20, 21], and such technologies are becoming increasingly central to biology. They have advantages, including greater opportunities for educational engagement [22], accelerated innovation, reduced cost, reduced redundant problem solving in different laboratories, and more rapid advancement of scientific discovery via a greater return on investment [23]. Such accessible technologies for high-throughput phenomics during the dynamic and sensitive process of embryonic development are urgently required, particularly within the context of assessing the impacts of global change [4].

Here, we present EmbryoPhenomics, an open-source high-throughput platform for phenomics in aquatic embryos that consists of an Open-source Video Microscope (OpenVIM), with experimental control over the embryonic environment, and a Python package Embryo Computer Vision (EmbryoCV) for high-dimensional measurement of phenomic traits from video data sets. The EmbryoPhenomics platform enables the quantification and integration of interindividual and intraindividual temporal change in morphological, physiological, and behavioural traits with high resolution and to an unprecedented scale in a largely automated workflow. We demonstrate the application of EmbryoPhenomics for quantifying integrated organismal responses to environmental change, using experiments assessing i) chronic, ii) acute, and iii) interactive effects of environmental stressors. Furthermore, we show the efficacy and transferability of this platform using two ecologically important species with radically different patterns of development: *Radix balthica*, a freshwater gastropod mollusc, and *Orchestia gammarellus*, a marine amphipod crustacean.

## Results

### EmbryoPhenomics platform overview

**Fig 1:**
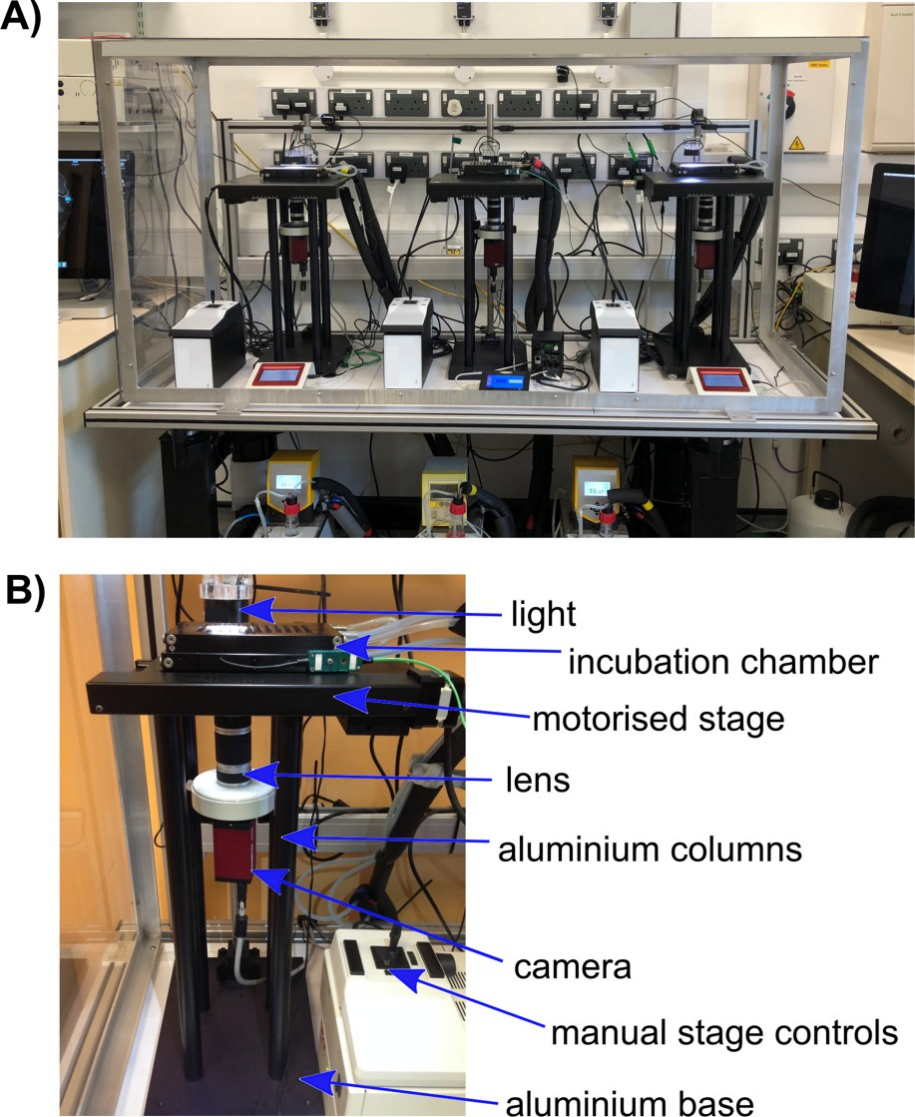
A. Photograph of three OpenVIM systems at University of Plymouth on a vibration insulating imaging table. B. Annotated photograph of a single OpenVIM system. OpenVIM, Open-source Video Microscope.

**Fig 2:**
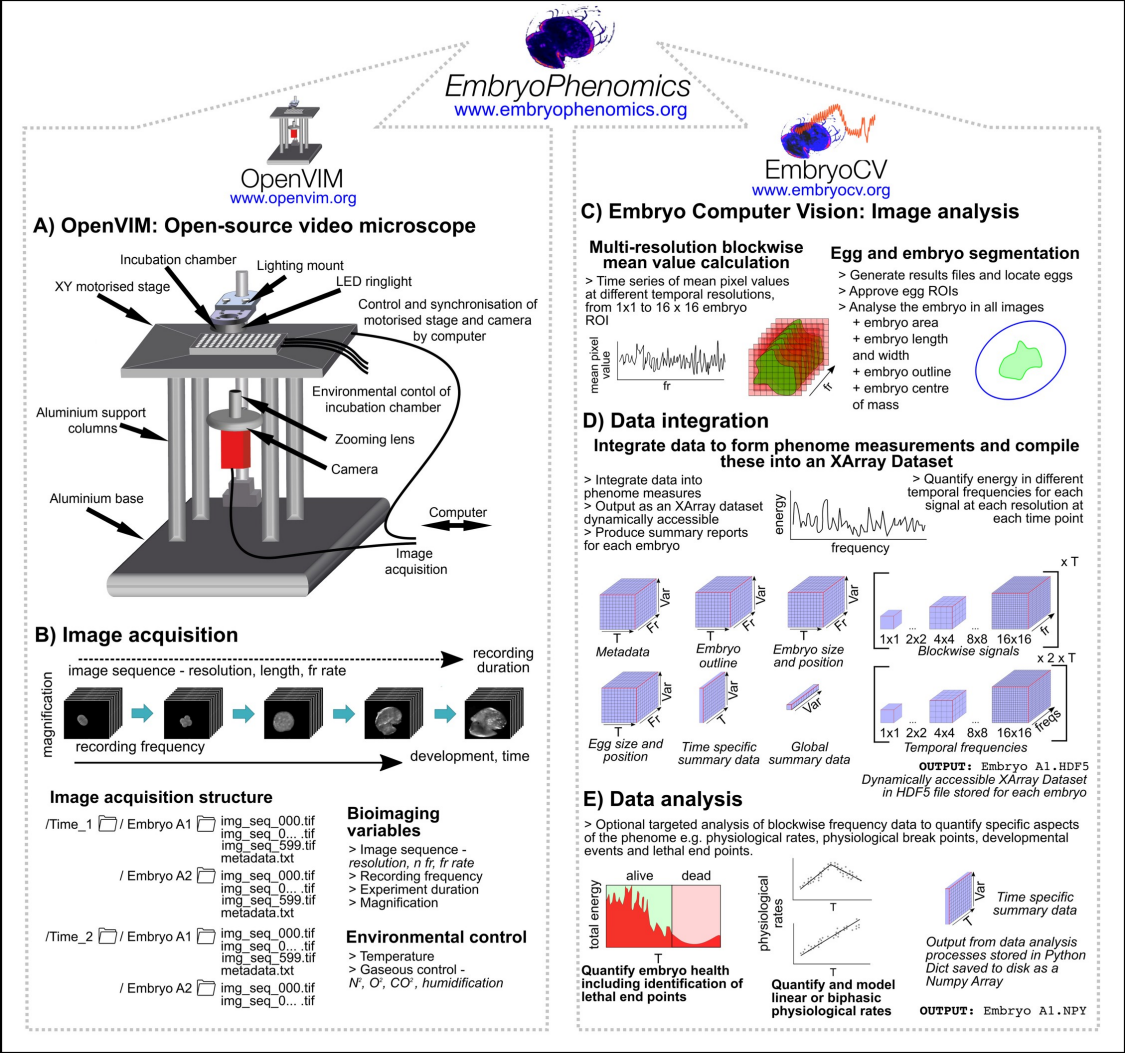
Schematic outline of the EmbryoPhenomics platform. A. OpenVIM: technical drawing of the OpenVIM bioimaging hardware with labelling of its different components. B. Image acquisition using OpenVIM: bioimaging and environmental variables controlled using OpenVIM in the process of performing an experiment and acquiring images. The structure of image acquisition and storage is outlined. C. EmbryoCV: outline of the processes within the three functional modules of EmbryoCV: Image Analysis, Data Integration, and Data Analysis. Data are output for each embryo as Pandas Data Panels [24] from the Image Analysis stage (C), HDF5 Xarray Dataset files [25] from the Data Integration stage (D), and Python Dictionaries in the Data Analysis stage (E). The structure of data within each stage are outlined, including their different dimensions. EmbryoCV, Embryo Computer Vision; fr, frame; freqs, frequency; HDF5, Hierarchical Data Format 5; OpenVIM, Open-source Video Microscope; ROI, region of interest; T, time; Var, phenomic variable.

The EmbryoPhenomics platform (www.embryophenomics.org) consists of open-source hardware (Figs 1 and 2A, OpenVIM, www.openvim.org) and software (Fig 2C–2E, EmbryoCV, www.embryocv.org, S1 Code). OpenVIM performs long-term imaging of large numbers of aquatic embryos with coupled environmental control (Fig 2B). EmbryoCV is a Python package for automated and robust analysis of the resultant video to extract and integrate large quantities of data to form biologically relevant phenome-level measurements.

OpenVIM can acquire images of large numbers of developing embryos at different temporal scales (S1 Video). The resultant image sequence time series can be used to visualise short-term changes in the physiology (e.g., heart rate) and behaviour (e.g., spinning and crawling rates) of embryos in real time throughout the course of an experiment and longer-term changes in form and function (e.g., morphometrics and physiology) through ontogeny. The use of high–depth-of-field optics (see Materials and methods) enables long-term and fully automated simultaneous recording of large numbers of embryos for the duration of their development, including species with embryonic life history stages lasting many weeks or even months.

Analysis of the image time series produced by OpenVIM is performed offline, following image acquisition, by EmbryoCV, a purpose-built Python based package (S1 Code, www.embryocv.org). The EmbryoCV software performs analysis and extraction of data from every image sequence acquired (Fig 2C, Image Analysis), and these are integrated to form data sets that include morphological, behavioural, physiological, and proxy measurements. Proxy measurements include the measurement of mean pixel intensities (i.e., grey-level intensities) at multiple resolutions within each frame of an image sequence to form time series signals that are subsequently analysed to quantify energy within different temporal frequency bins (Fig 2D, see Materials and methods for more information). These proxy traits can be used for quantification of both holistic (lethal and sublethal classification) and specific measures (quantification and modelling of cardiac rates) of embryo health and physiology (Fig 2E).

**Fig 3:**
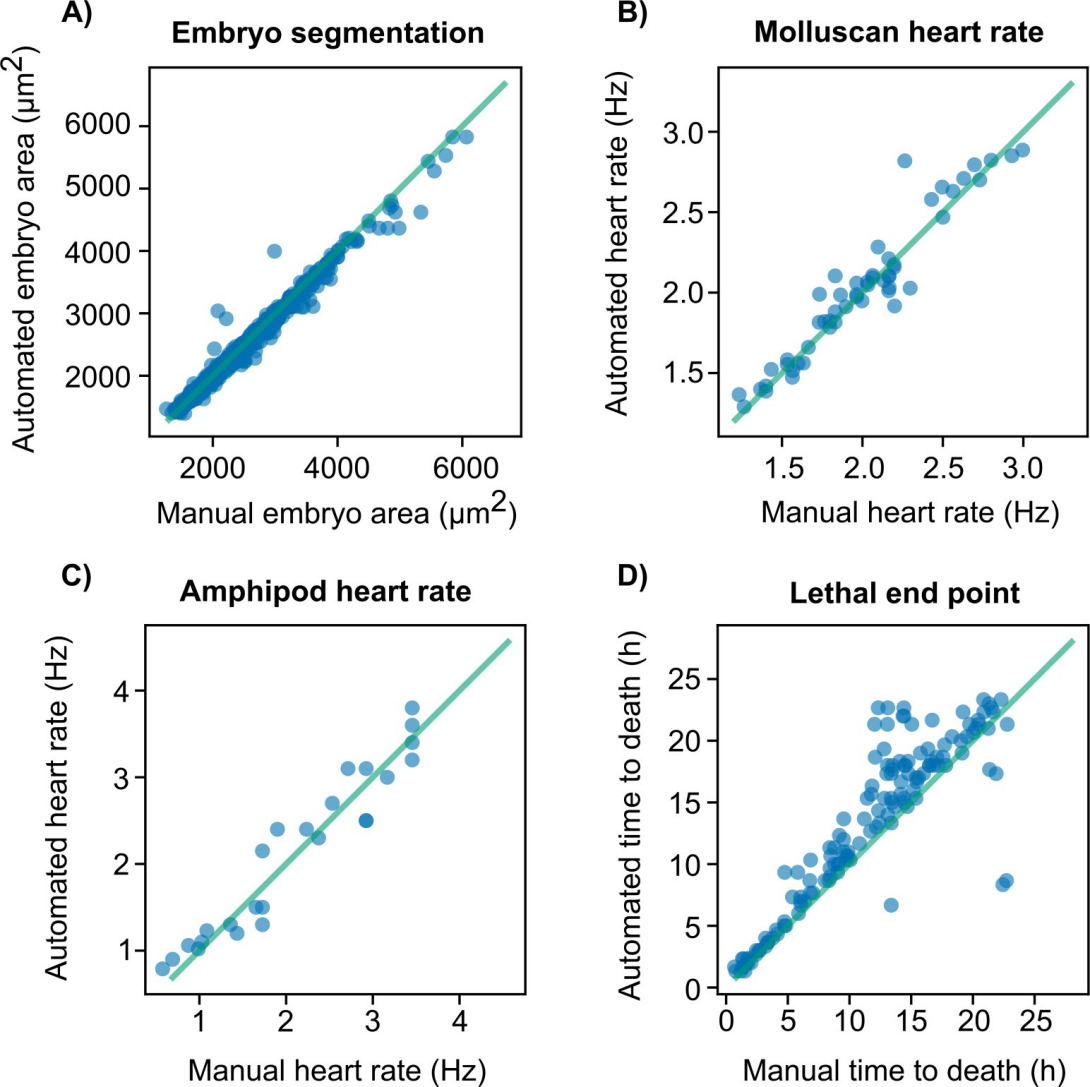
Comparison of data produced by EmbryoCV with manually quantified measures (S1 Data). A. Area of *R*. *balthica* embryos determined via both manual drawing of ROIs and automated embryo segmentation (r_614_ = 0.989, *P* < 0.001). B. Heart rate of *R*. *balthica* embryos determined using both manual video analysis and the automated EmbryoCV identifyHeartRateForAllEmbryos process (r_52_ = 0.956, *P* < 0.001). C. Heart rate of *O*. *gammarellus* determined using manual video analysis and the automated EmbryoCV identifyHeartRateForAllEmbryos process (r_25_ = 0.961, *P* < 0.001). D. Lethal end points (time to death) for *R*. *balthica* embryos at E3, E7, and E9 developmental stages was determined using manual video analysis, and automated measurements were generated using the lethalEndPoint EmbryoCV process (r_140_ = 0.892, *P* < 0.001). EmbryoCV, Embryo Computer Vision; ROI, region of interest.

The accuracies of measurements made by EmbryoCV were assessed via comparison with manual quantification of spatial and temporal traits equivalent to the measurements performed by EmbryoCV in a randomised subset of images from each experiment. Concordance between measurements made manually and those made by EmbryoCV was high for both spatial and temporal measurements (Fig 3, see Materials and methods for more information).

The ability of the EmbryoPhenomics platform to quantify integrated organismal responses to environmental change was assessed using experiments of different designs: i) chronic, ii) acute, and iii) interactive effects. Furthermore, we demonstrate the transferability of the platform using two ecologically important species with different patterns of development: *R*. *balthica*, a freshwater gastropod mollusc, and *O*. *gammarellus*, a marine amphipod crustacean. These experiments incorporated the acquisition of >30 million images of 623 embryos by OpenVIM, of which 95% were successfully analysed using EmbryoCV. Due to abnormal development, 4% of embryos were removed manually from the analysis, and of the remaining embryos, 98% were successfully characterised using the EmbryoCV workflow (Table 1).

**Table 1:** Overview of experimental treatments, design, and image-acquisition parameters.

Treatment	*N* Embryos imaged	Acquisition details (RS, FR, ROI, data acquired, magnification)	Duration (min–max; h)	Total images acquired	Successfully analysed (embryos; images)	Embryos manually removed (insufficient data; abnormal development)
**Experiment 1**		RS = 600 frames, repeated hourly; FR = 20 fps; ROI = 750 x 750 px; bit depth = 16 bits; image data = 21 TB; phenome data = 510 GB; magnification = 200x				
20°C	48		292–367	7.53 M	42; 7.14 M	0; 6
25°C	48		292–364	5.37 M	44; 4.66 M	0; 4
30°C	48		196–269	4.83 M	32; 4.51 M	1; 14
Total	144			17.73	119; 16.32 M	1; 24
Sample video and data sets			https://doi.org/10.5281/zenodo.1419971			
**Experiment 2**		RS = 600 frames, repeated 20 min; FR = 20 fps; ROI = 750 x 750 px; bit depth = 16 bits; image data = 9.2 TB; phenome data = 121 GB; magnification = 200x				
36°C, St = E3	63		24 h	2.52 M	58; 2.44 M	0; 0
36°C, St = E7	56		24 h	2.42 M	56; 2.41 M	0; 0
36°C, St = E9	64		24 h	2.76 M	64; 2.75 M	0; 0
Total	183			7.7 M	178; 7.6 M	0; 0
Sample video and data sets			https://doi.org/10.5281/zenodo.1419226			
**Experiment 3**		RS = 600 frames, repeated hourly; FR = 20 fps; ROI = 750 x 750 px; bit depth = 16 bits; image data = 5 TB; phenome data = 44.17 GB; magnification = 200x				
20°C, S = 0	48		24 h	720,000	46; 679,650	2; 0
20°C, S = 5	48		24 h	720,000	46; 681,720	2; 0
25°C, S = 0	48		24 h	720,000	48; 704,160	0; 0
25°C, S = 5	48		24 h	720,000	46; 681,030	2; 0
30°C, S = 0	48		24 h	720,000	47; 698,655	1; 0
30°C, S = 5	48		24 h	720,000	44; 636,240	4; 0
Total	288			4.3 M	277; 4.24 M	11; 0
Sample video and data sets			https://doi.org/10.5281/zenodo.1419207			
**Experiment 4**		RS = 2100 frames, repeated hourly) FR = 38 fps; ROI = 700 x 700 px; bit depth = 16 bits; image data = 4.6TB; phenome data = 44.17 GB				
15°C	4		24 h	201,600	4; 201,600	0; 0
20°C	4		24 h	201,600	4; 201,600	0; 0
Total	8			403,200	8; 403,200	0; 0
Sample video and data sets			https://doi.org/10.5281/zenodo.1420304			
**TOTAL**	**623**			**30.13 M**	**582; 28.52 M**	**12; 24**

**Abbreviations:** fps, frames per second; FR, frame rate; GB, gigabyte; M, million; px, pixels; ROI, region of interest; RS, recording schedule; TB, terabyte.

### Experiment 1: Developmental responses to chronic elevated temperatures

**Fig 4:**
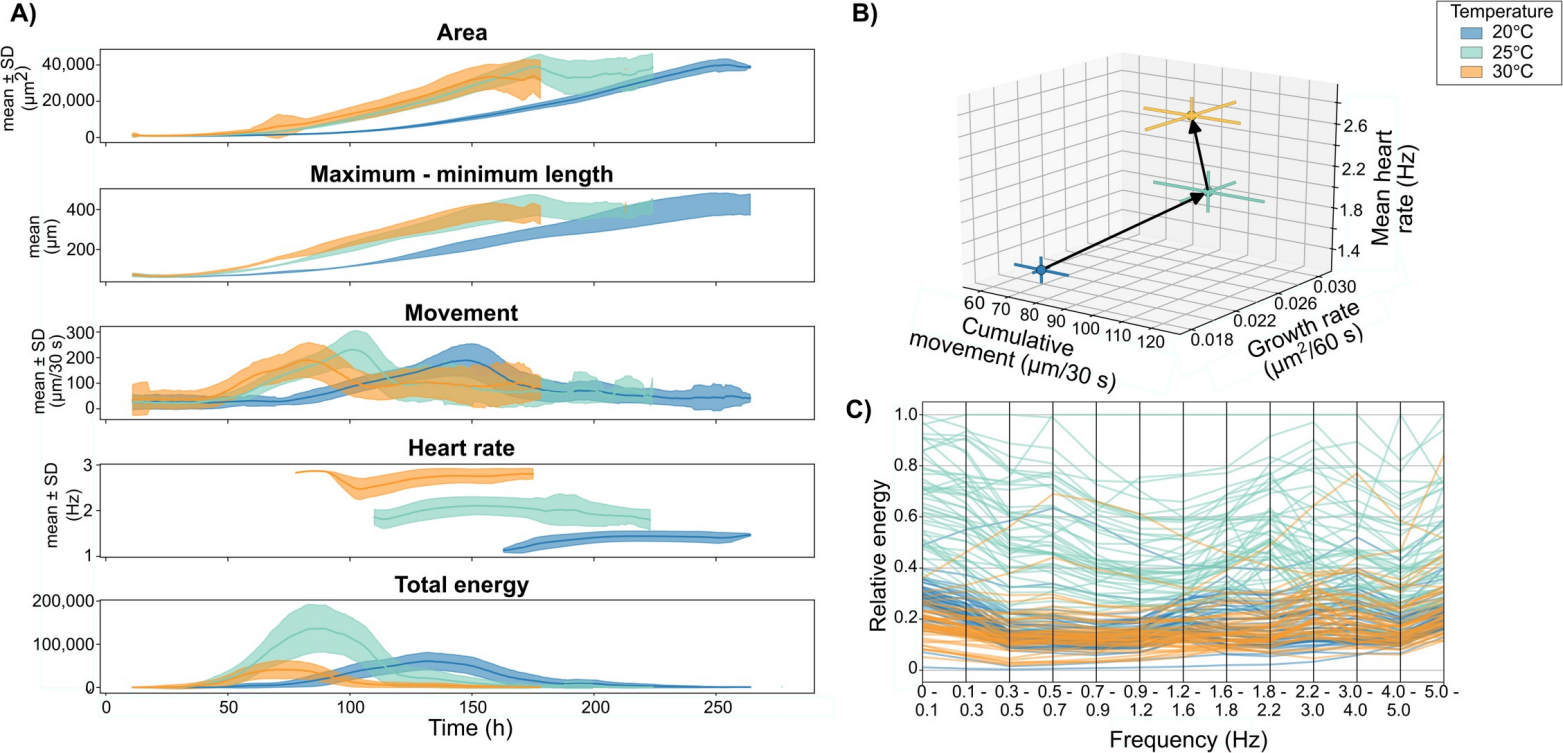
A. Developmental time series of a range of phenome components in *R*. *balthica* cultured under contrasting temperatures (S2 Data). B. The response of morphological growth rate, physiological (heart rate), and behavioural (cumulative movement) parameters of *R*. *balthica* embryos cultured under contrasting temperatures (mean ± 1 SD). C. Parallel coordinate plot of the relative energy within different frequency bin ranges during the development of individual *R*. *balthica* embryos cultured under contrasting temperatures.

We examined phenome-level differences of embryos maintained in contrasting environments from the first cell division until hatching (Fig 4). One cell–stage *R*. *balthica* embryos were placed in three temperatures (T = 20, 25, and 30°C, n = 48 for each temperature) and recorded using OpenVIM for 30 s at 20 frames per second every hour for the duration of their development. This generated a total of 17.73 million images for all 144 embryos and these were subsequently analysed by EmbryoCV. The embryo was successfully identified and measured by the Image Analysis stage of EmbryoCV in 16.32 million of the acquired images (92%) and in 143 of the 144 embryos recorded using the OpenVIM (Table 1). Further to the core EmbryoCV steps of Image Analysis and Data Integration, an additional Data Analysis process (Fig 2E) was used to quantify the ontogeny of cardiac function for each embryo and to fit an appropriate model to these data. The ontogeny of cardiac function in *R*. *balthica* exhibits a segmented pattern, and consequently, a segmented regression model was applied to the heart rate measurements extracted for each embryo within the EmbryoCV function measureHeartRatesForAllEmbryos.

The measurements made by EmbryoCV revealed significant effects of temperature on growth rates (F_2, 51.6_ = 212.28, *P* < 0.001), rates of movement (F_2, 64.7_ = 58.1, *P* < 0.001), and heart rates (F_2, 59.6_ = 661.7, *P* < 0.001) but also revealed a marked difference in the direction and magnitude of responses between temperature increments (Fig 4A and 4B). An increase in movement, growth rate, and heart rate was evident in embryos grown in 25°C compared with 20°C. However, between 25°C and 30°C, growth rate and rates of movement decreased marginally, yet heart rate was significantly accelerated well beyond the net increase observed between 20°C and 25°C. Restriction in the extent to which growth rates and rates of movement can be accelerated in response to temperature are of biological interest and suggest that chronic exposure to higher temperatures may prove problematic for *R*. *balthica*. This is further supported by a reduction in the ‘Total Energy’ proxy trait in the time series at 30°C compared to both 20°C and 25°C. Total Energy is a measure of the power within pixel intensity (i.e., grey-level) fluctuations at all temporal frequencies in the embryo. Consequently, a reduction in the Total Energy proxy trait (Fig 4B) is indicative of a reduction in the overall activity of embryos, including both net and gross embryo movements. In addition to a reduction in Total Energy, there was also a corresponding reduction in the energy across frequency bands in 30°C embryos (Fig 4C).

### Experiment 2: Lethal end points for different developmental stages

To assess the capability of the EmbryoPhenomics platform for measuring extreme (i.e., lethal) biological responses, we used an acute 24-h exposure of three developmental stages (E3^*n* = 63^, E7^*n* = 56^, E9^*n* = 64^) of *R*. *balthica* embryos to an elevated temperature of 36°C. OpenVIM recorded individual embryos for 30 s at 20 frames per second every 20 minutes for 24 h. This generated a total of 7.7 million images for all 183 embryos, and these were subsequently analysed by EmbryoCV. We assessed the ability of EmbryoCV to quantify the occurrence of lethal end points (time to death) within developmental stages that exhibit different forms of biological response. In addition to the core EmbryoCV processes of Image Analysis and Data Integration, an additional Data Analysis process—identifyLethalEndPoint—was used to identify time to death for each embryo from the data that had been collected in the preceding stages, including size, movement, and energy within different frequency bands.

**Fig 5:**
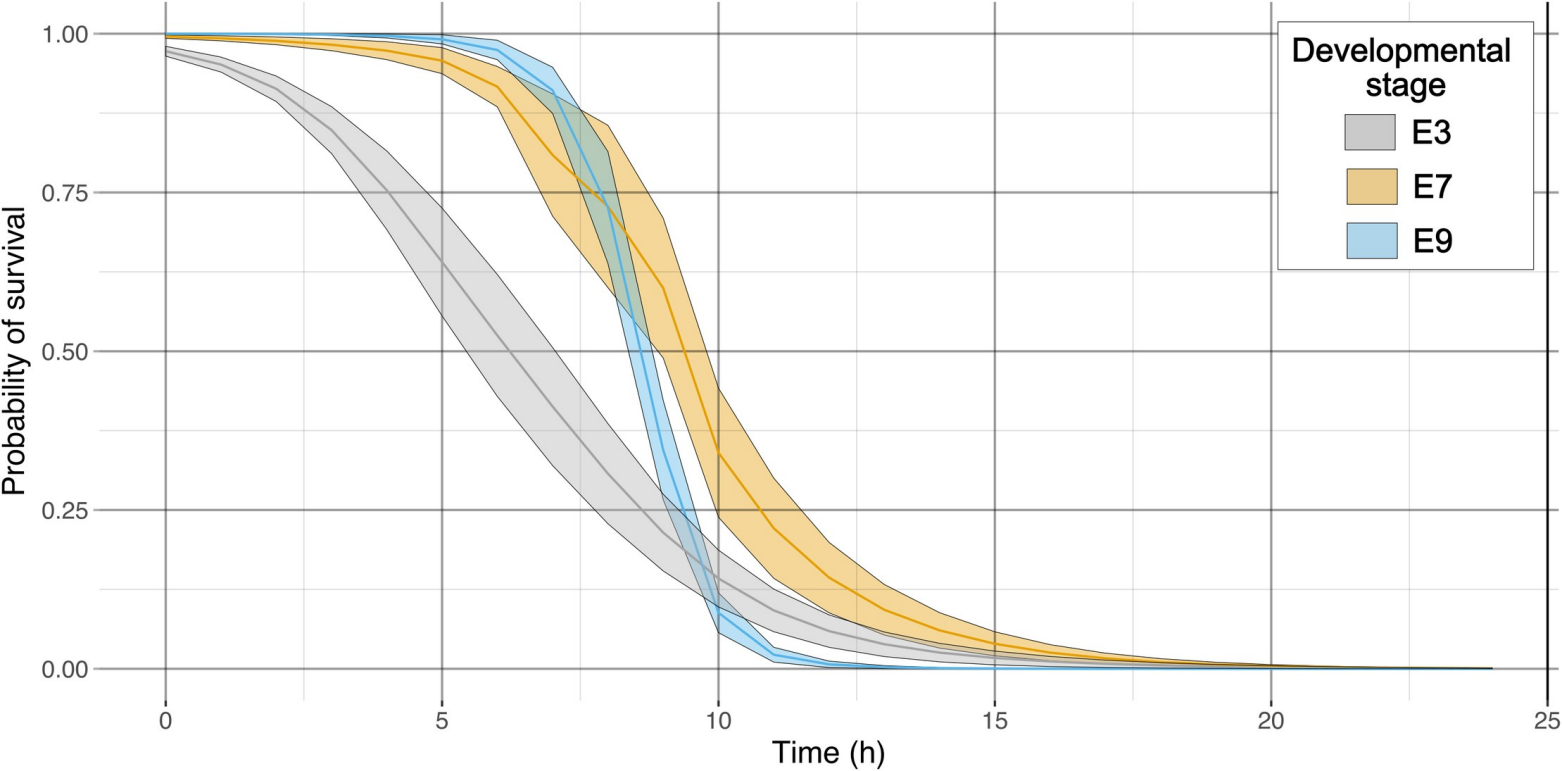
Duration of exposure-dependant probability of survival curves at 36°C for different developmental stages of *R*. *balthica* (S3 Data).

Of the 183 embryos studied, 93% were correctly identified as exhibiting a lethal end point. Concordance with manually determined lethal end points was high for all three developmental stages (E3 = 98.8%, E7 = 99.3%, E9 = 99.3%; Fig 3D). The sensitivity of the three developmental stages, E3, E7, and E9, to elevated temperature, as measured by the median lethal time (LT) for a proportion of organisms, was significantly different (Fig 5; LT25 − F_2,20_ = 4.98, *P* = 0.018; LT50 − F_2,20_ = 11.09, *P* < 0.001; LT75 − F_2,20_ = 14.04, *P* < 0.001). The earliest developmental stage (E3) had a lower LT50 (8.6 h ± 1.82) and LT75 (4.11 h ± 0.98) than the two later stages (E7: LT50 = 22.94 h ± 3.21, LT75 = 14.59 h ± 2.31; E9: LT50 = 16.21 h ± 1.81, LT75 = 13.28 h ± 1.21), indicating a greater sensitivity to thermal stress. These early stage embryos exhibited a ciliary-driven spinning behaviour, and their lethal end point coincided with a loss of osmotic control, made visual by a rapid increase and subsequent decrease in embryo area. Automated peak identification was used in the EmbryoCV function identifyLethalEndPoint to identify this lethal end point. E7-stage embryos possess a weak heartbeat and a transitionary form of locomotion consisting of part ciliary-driven gliding and part muscular crawling on the egg capsule wall, whereas E9-stage embryos possess a strong heartbeat, radula movements, and muscular contractions. Lethal end points in E7- and E9-stage embryos were indicated by a loss of cardiac function and a cessation of gross embryo movements. The EmbryoCV identifyLethalEndPoints function identified this response in these later developmental stages and detected lethal end points via a decrease in the relative energies within specific summed frequency bins within the energy proxy traits.

### Experiment 3: Short-term responses to multiple stressors

**Fig 6:**
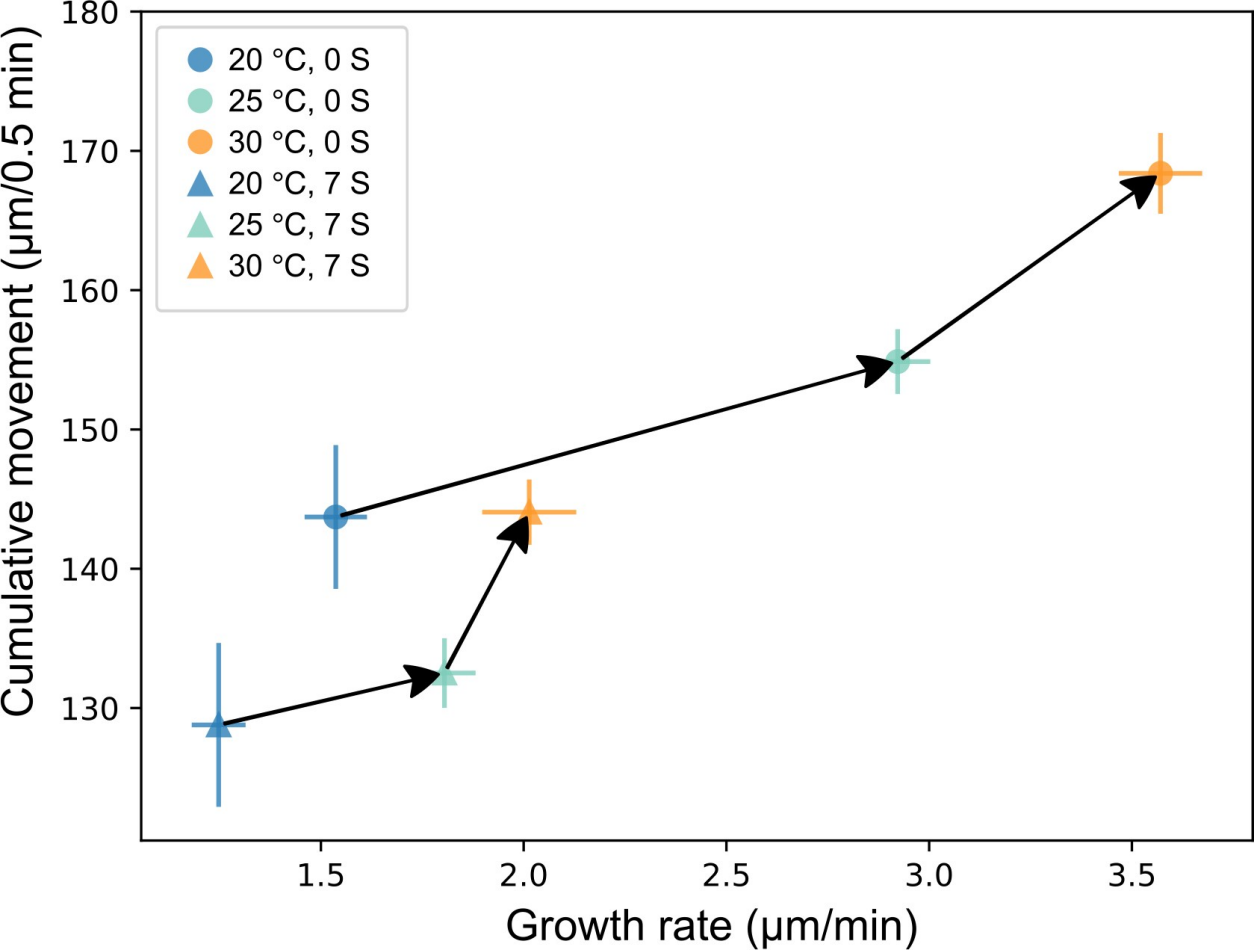
Movement and growth rate responses of E3 developmental–stage *R*. *balthica*^(*n* = 277)^ exposed to combinations of temperature and salinity for a period of 24 h (means ± 1 SD, S4 Data).

The ability of the EmbryoPhenomics platform to detect complex multifaceted responses within multistressor experiments was assessed using a 24-h exposure of E3-stage *R*. *balthica* embryos (*n* = 288) to contrasting temperatures (20°C, 25°C, 30°C) and salinities (the degree of being saline, 0 or 7 parts per thousand). OpenVIM recorded embryos for 30 s at 20 frames per second for 24 h. This generated a total of 4.3 million images for all 288 embryos, and these were subsequently analysed by EmbryoCV (Fig 6). There were significant effects of temperature and salinity on movement (temperature, F_2, 271_ = 17.06, *P* < 0.001; salinity, F_1, 273_ = 43.08, *P* < 0.001) and significant effects of, and a significant interaction between, temperature and salinity on growth rate (temperature, F_2, 259_ = 138.2, *P* < 0.001; salinity, F_1, 261_ = 149.4, *P* < 0.001; temperature*salinity, F_2, 261_ = 27.8, *P* < 0.001).

In a salinity of 0, EmbryoCV identified increases in both cumulative movement and growth rate that were associated with an increase in temperature from 20°C to 25°C; however, from 25°C to 30°C, the increase in growth rate was markedly reduced despite a similar increase in movement. At a salinity of 7, both growth rate and movement were reduced at each temperature, relative to the 0-salinity treatment. Furthermore, the proportionate magnitude of response comparing 20°C to 25°C and 25°C to 30°C was more pronounced in salinity 7, with a significant interaction in the response of embryo growth rates to temperature and salinity. This suggests that the ability to tolerate elevated temperatures was compromised by the addition of a salinity stress.

### Experiment 4: Effects of temperature on the ontogeny of cardiac function

The EmbryoCV software and OpenVIM hardware have been developed to be versatile and extendible to a wide range of species and scientific applications. A core process within EmbryoCV is the use of a multiresolution blockwise signal quantification that is capable of quantifying embryonic traits with different functional forms and of capturing embryo responses as holistic proxy measures.

**Fig 7:**
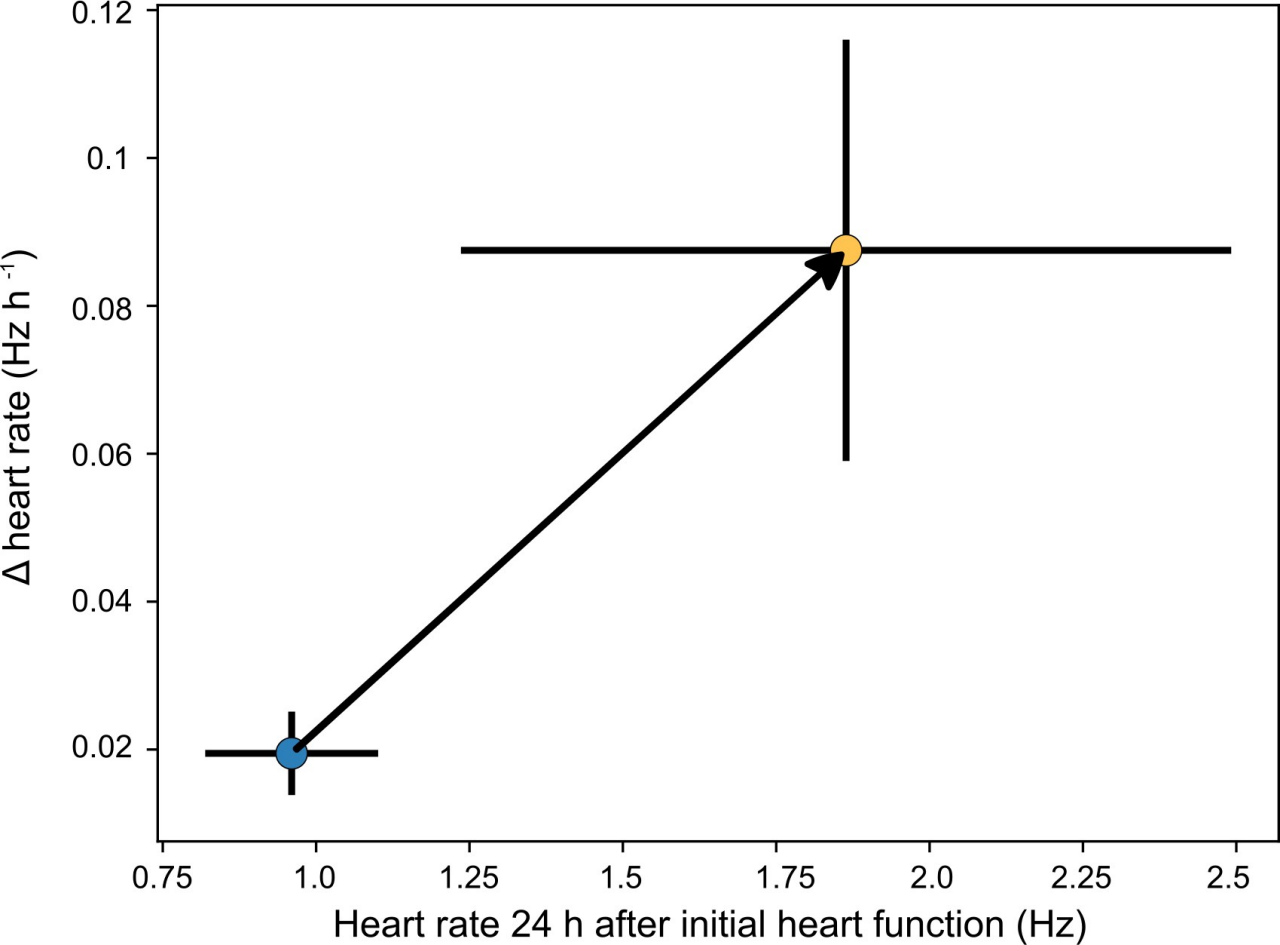
Early cardiac ontogenic response to elevated temperature in *O*. *gammarellus*^(*n* = 8)^. Blue = 15°C, orange = 20°C (means ± 1 SD, S5 Data).

During the development of *R*. *balthica*, the embryo exhibits both ciliary-driven rotation and a muscular crawling behaviour within the egg capsule. Furthermore, *R*. *balthica* possesses a two-chambered globular heart and undergoes the process of torsion in which its mantle cavity (where the heart is located) rotates by 180°. The automated quantification of cardiac function in *R*. *balthica* is demonstrated in Experiment 1, and here, we apply the same process to quantifying the response of cardiac function in the intertidal amphipod *O*. *gammarellus* during a 24 h period in contrasting temperatures (Fig 7). The development and morphology of *O*. *gammarellus* is markedly different to *R*. *balthica*. *O*. *gammarellus* fills its egg capsule and possesses a tubular heart positioned dorsoventrally and with a cardiac rate approximately double that of *R*. *balthica*. Despite these differences, EmbryoCV achieved high levels of concordance for measurements of cardiac rate compared to manual measurements for *O*. *gammarellus* (see Fig 3C).

OpenVIM recorded *O*. *gammarellus* embryos maintained at either 15°C or 20°C for 54 s at 38 frames per second every hour from 24 h to 48 h after the initial onset of cardiac function. Rates of increase in heart rate from 24 h to 48 h following first heart function were significantly different (H_1_ = 5.33, *P* = 0.021) and approximately four times faster in 20°C than 15°C. Such high rates of acceleration in the physiological ontogeny of cardiac function in response to a 5°C increase in temperature are interesting and suggest a high thermal sensitivity of *O*. *gammarellus*.

## Discussion

Here, we demonstrate the ability of the open-source EmbryoPhenomics platform to quantify complex phenome-level responses of large numbers of developing aquatic embryos. EmbryoPhenomics is versatile and we demonstrate its application to a broad range of experimental designs, ranging from 2-week to 24-h experiments, and demonstrate its application to two species. These experiments include assessment of responses to acute and chronic thermal stress, arguably one of the greatest current threats to biodiversity and one to which early life stages can have heightened sensitivity [26]. EmbryoPhenomics was also used to quantify the response of 288 early developmental–stage *R*. *balthica* embryos to combined thermal and saline stress and revealed an inhibitory effect of salinity on growth and movement that contrasted with the effects of elevated temperature. The generation of high-dimensional data is a prerequisite to phenomics, and owing particularly to the dynamic nature of embryonic development, this capability will facilitate advances in our understanding of this pivotal life stage. EmbryoCV and OpenVIM are versatile, extendible, and open-source technologies applicable to a range of species and study designs. Our aim is that these resources will develop a community of users, in a similar model to that seen with the Open Selective Plane Illumination Microscopy (OpenSPIM) project [19]. In contrast to more restrictive and focused technologies for embryo phenotyping [27, 28], both OpenVIM and EmbryoCV are intentionally modular, versatile, high throughput, and transferrable to species with contrasting developmental patterns. EmbryoPhenomics can therefore underpin the generation of data describing whole-organism responses with a scale, diversity, and quality that is required to study embryonic development in a manner befitting phenomics, which compares with that produced using molecular-omic technologies.

Concordance between manually and EmbryoCV-determined measures of embryo spatial and temporal characteristics was high (Fig 3). The blockwise signal and frequency workflow was effective at locating and quantifying both the two-chambered globular heart of *R*. *balthica* but also the elongated, tubular heart of *O*. *gammarellus*. The typical maximum cardiac rate of *R*. *balthica* at 20°C is 1.5 Hz, whereas *O*. *gammarellus* has a higher cardiac rate of 3.5 Hz with sustained periods of diapause, presenting challenges in the effective manual quantification of rate. Here, for *O*. *gammarellus*, to account for the skew introduced by diapause, beat-to-beat timings were recorded manually, and the rates calculated from the median timing were closely correlated with the frequency measurements produced by EmbryoCV. For *R*. *balthica*, direct comparisons of manually determined rate, i.e., counts of heartbeats, were closely correlated with EmbryoCV determined rates. Movements of the embryo meant that the heart and other features of interest were not always visible in the image. Consequently, EmbryoCV applies spectral frequency analysis across different areas of the embryo and attempts to identify biologically relevant frequencies for modelling heart rate. These data then inform the fitting of models to heart rate, and the resultant cardiac rates were closely correlated to manual measures for both species in Experiments 1 and 4. A similar workflow also underpins the automated identification of lethal end points across the three developmental stages of *R*. *balthica* studied in Experiment 2. Here, developmental stage–specific algorithms identifying rapid increases in size indicative of osmotic control, rapid reductions in energy within particular frequency bands, or a combination of the two and were highly effective at producing classifications of lethal end points closely aligned with those ascertained manually. Further optimisation and mining of the data produced by EmbryoPhenomics will allow tailored end points to be identified, including responses that, while present, are not immediately apparent to human observers in the multitemporal video produced by OpenVIM.

Phenomics offers the potential to facilitate rapid advances in our understanding of biology via the generation of a quantity and quality of phenotype-level data that is more appropriate to addressing questions focused on understanding the complexities of organismal biology. The EmbryoPhenomics platform provides the technological capability for the study of organismal development in a way that captures temporal, spatial, and functional diversity at both interindividual and intraindividual levels—a task identified as a major challenge in biology [29, 11]. Here, we demonstrate the capability of OpenVIM to document the development of large numbers of aquatic embryos (*N* > 600) in experimental treatments ranging from long- (>240 h) to short-term (24 h) exposures. Of the >30 million images acquired across the four experiments described here, EmbryoCV successfully extracted phenome-level data from 95%, producing high dimensional data describing morphological, physiological, and behavioural embryo responses.

## Materials and methods

### OpenVIM

Image acquisition in the EmbryoPhenomics platform is achieved using OpenVIM, a modular, custom assembled video microscope that enables long-term automated image acquisition of large numbers of aquatic embryos. OpenVIM is modular and can incorporate a range of different components, enabling a wide range of operating parameters (Figs 1 and 2; see S1 Table for a guide to components and S1 Text for an assembly guide). The OpenVIM configuration applied in the current studies is a Charge Coupled Device camera (monochrome, resolution: 2048 x 2048; Pike 421B, Allied Vision Technology, Stradtroda, Germany) connected to high–depth-of-field optics (magnification: 20–200x; VHZ20R, Keyence, Milton Keynes, United Kingdom) inverted and mounted in an aluminium frame atop which an XY motorised stage (Scan, Marzhauser, Wetzlar, Germany) is fixed (Fig 1A). The motorised stage houses an incubation chamber (T range: min = 10–15°C below ambient, max = 60°C; Bold Line Cryo, OkoLab, Naples, Italy) for multiwell plates, providing temperature control and high levels of humidification to minimise evaporation. Dark field lighting is provided by an LED ring light (LDR2-42-SW2, CCS, London, UK) mounted above the incubation chamber. The camera and motorised stage are synchronised using MicroManager [30], a plugin for ImageJ [31].

Image acquisition is performed using the Multidimensional Acquisition function in MicroManager. A sequence of images is acquired of each individual embryo in succession, and this process is repeated for the duration of the experiment using a Beanshell script (S2 Code). Images are stored as individual sequences of TIFF format 16-bit images with accompanying metadata, and these are written to 6-TB hard drives (ST6000DM004, Seagate, Dublin, Ireland) using a hard drive enclosure (TeSU, DATOptic, California, United States of America) for offline processing using EmbryoCV. Compiled video file samples from each treatment within each experiment are provided in a Zenodo repository—refer to Table 1 for the DOI link to each experiment.

### EmbryoCV

The software component of the EmbryoPhenomics platform is EmbryoCV (www.embryocv.org), a Python [32] class written in Python 2.7 (S1 Code). EmbryoCV has dependencies, including the open-source libraries OpenCV [33], Sci-Kit [34], Numpy [34], Pandas [24], XArray [25], Matplotlib [35], and Pyqtgraph. Python is an increasingly popular language with biologists [36] and was used to develop EmbryoCV to maximise the utility and extendibility of this platform by potential users.

#### Workflow

The code of EmbryoCV is structured as a series of functional modules: EmbryoCV.py, dataHandling.py, imageAnalysis.py, dataIntegration.py, dataAnalysis.py, and eggUI.py. But the user experience is intentionally simple, consisting of the following user callable functions:

1. Initiating an EmbryoCV analysis: users begin by creating an instance of EmbryoCV and provide it with some information about the experiment. EmbryoCV will generate a results file for each embryo and extract information from MicroManager metadata for each image sequence, including the time of acquisition for each image. Furthermore, at this stage, EmbryoCV attempts to locate the egg in each image sequence.
  Command: experiment = embryocv.embryocv(‘pathToFiles','new',scale = micrometers_per_pixel, species = ‘speciesname’).
2. Validating egg identification: an optional stage during which a user interface is used for modifying the egg ROI size, shape, and position prior to beginning the more computationally demanding aspects of the analysis.
  Command: experiment.validateEggs()
3. Quantify embryo traits: measurement of size, shape, position, and multiresolution blockwise signals are made from every image within each image sequence and stored to the embryo results files. Multiresolution blockwise signals of mean pixel intensity are produced at different spatial resolutions 1 x 1 (whole embryo ROI), 2 x 2, 4 x 4, 8 x 8, and 16 x 16. Power spectral densities within different temporal frequencies are then calculated using Welch’s method from the signal module of Scipy [34, 37] to produce a spectrum of power within different frequency bands within different resolutions of each image sequence.
  Command: experiment.quantifyAllEmbryos()
4. Integrate embryo traits: raw measurements from the preceding step are integrated to form biologically relevant measures, including time-specific measurements such as minimum, maximum, and mean size and movement at each time point during the experiment but also global measurements such as growth rate. Frequency analysis is also applied to the blockwise signals generated in the preceding step to quantify energy within different frequency bands. At this stage, data are transformed into a dynamically accessible XArray Dataset.
  Command: experiment.savePhenomeMeasuresForAllEmbryos(‘pathToSave’)
5. Focussed data analysis optional steps:
  - Generate summary reports: produces PDF reports for individual embryos, including developmental time series of time, movement patterns, and energy within different frequency bands.
    Command: experiment.generateSummaryReports(‘pathToSave’)
  - Quantify and model cardiac rates: identify cardiac rates from within the frequency data output and fit either a segmented (*R*. *balthica*) or linear (*O*. *gammarellus*) model. Time series of cardiac rates, including parameters from the model are output.
    Command: experiment.measureHeartRateFoAllEmbryos(‘pathToSave’)
  - Quantify lethal end points: use data from previous processes to identify lethal end points in different stage embryos, optimised to work with E3-, E7-, and E11-stage *R*. *balthica* embryos, using either reductions in energy within particular frequency bands or sudden increases in size indicative of a failure of osmotic control or a combination of the two.
    Command: analysis.identifyLethalEndPoints(‘pathToSave’, ‘developmentalStage’)

The experiments described in this paper were analysed on Apple Mac computers (MacPro, 12 core, 64-GB RAM). The most computationally intensive stage of EmbryoCV is quantifyAllEmbryos (Fig 2C), during which the embryo is segmented (approximately 20 frames s^-1^) and data are stored to disk as a Pandas Dataframe via pickle. A high proportion (>95%) of the 30.03 million images acquired by OpenVIM across the four experiments analysed were successfully analysed by EmbryoCV.

### Manual validation

#### Spatial accuracy

Assessment of the accuracy of the embryo size measurements produced by EmbryoCV was performed by comparison of manual and automated measurements of embryo area (Fig 3). Images (*N* = 617) were randomly selected from Experiment 3 and were presented to users (*N* = 7) for manual analysis in Fiji [21] with a simple ActionBar [38] user interface. Users applied the polygon tool to draw manually around the perimeter of the embryo, and the results were recorded to file, together with the image ID, using an ActionBar macro (S3 Code). The area of these manually recorded outlines was determined using the same OpenCV procedure as for the outlines generated by EmbryoCV.

#### Temporal accuracy

Heart rate measurements performed by EmbryoCV were validated by comparison with heart rate measurements made by performing manual video analysis. The heart rate of *R*. *balthica* is significantly slower than in *O*. *gammarellus* and more regular in its rhythm. Consequently, manual counts of the number of heartbeats visible within the video at a particular time point were considered a reliable benchmark against which to compare the heart frequency identified for *R*. *balthica* embryos by EmbryoCV.

*O*. *gammarellus* has an irregular heart rhythm, including extended periods of asystole; therefore, it was necessary to record the timing of individual heartbeats to enable a representative heart frequency to be calculated via analysis of beat-to-beat timings. A Fiji macro (S4 Code) was used to record the active frames in an image sequence at which each heartbeat occurred via manual pressing of a space bar, and this was subsequently used to generate a time series of beat-to-beat timings. Owing to the influence of asystole in producing a non-normal distribution of beat-to-beat timings, the median beat-to-beat timing was calculated, and this was used in comparison with the heart rate quantification performed by EmbryoCV.

In Experiment 2, the accuracy of lethal end points for *R*. *balthica*, recorded as time to death, was assessed using a comparison with manually determined lethal end points for all embryos. Mortality was defined as the time at which all visible signs of life ceased, including body movements and heart function. Manual video analysis was used to determine the time of death, and this was compared with the lethal end points determined using the identifyLethalEndPoints function in EmbryoCV. Of the 157 embryos analysed, 93% were identified correctly as exhibiting a lethal end point during the experiment. Of the 7% that were misidentified, 25% were identified as having a lethal end point, contrary to the lack of manually identified lethal end points, and 75% had no lethal end point identified. Concordance between manual and automated lethal end points was high in all three developmental stages (Fig 3D).

#### Experimental designs

Experiments 1–3 used *R*. *balthica* embryos produced in the laboratory from a source population at Chilton Moor on the Somerset Levels, UK (51.19° N 2.88° W) and maintained in the laboratory for a minimum of 7 days prior to experiments. Snails were maintained at 15°C in a 20 L aquaria containing Artificial Pond Water (APW, ASTM 1980) with 90 mg Ca^2+^/litre [39]. Water was changed twice weekly and snails were fed lettuce and spinach ad libitum. Eggs were dissected from egg masses under low-power microscopy (Leica MZ12, x40) and were placed in individual wells of a 96-well microtitre plate (Greiner Bio-One) before being inserted into the incubation chamber of the OpenVIM within the relevant treatment solution (Experiments 1–2: APW, Experiment 3: APW and APW combined with Instant Ocean to form a salinity of 7 parts per 1,000) and at the relevant treatment temperature (Experiment 1: 20°C, 25°C, and 30°C; Experiment 2: 36°C; Experiment 3: 20°C, 25°C, and 30°C). Water was changed every 2 days.

*O*. *gammarellus* were collected by hand from Mount Batten beach (50.35° N 4.13° W) and were maintained in a 1-L aquaria on damp filter paper at 15°C and were fed carrot ad libitium. Embryos were harvested from mothers within 48 h of collection via extraction using a paint brush from the brood pouch. They were then staged and inserted into a prewarmed multiwell plate at the relevant treatment temperature (15°C, 20°C). Water was changed every 2 days. Automated image acquisition using OpenVIM and subsequent image analysis using EmbryoCV was performed for each experiment as outlined in Table 1—sample video and EmbryoCV data from each experiment can be accessed via the DOIs in Table 1.

## Supporting information

S1 Code EmbryoCV Python class, containing the following components: EmbryoCV.py, dataHandling.py, imageAnalysis.py, dataIntegration.py, dataAnalysis.py, and eggUI.py. EmbryoCV, Embryo Computer Vision. (ZIP)

S2 Code A sequence of images is acquired of each individual embryo in succession, and this process is repeated for the duration of the experiment using a Beanshell script. (TXT)

S3 Code ImageJ ActionBar macro used for manual analysis of phenotypic traits. Interface provides users ability to measure and record embryo (polygon tool) and egg outlines (ellipse), with automated saving of results, for comparison with EmbryoCV-determined measures. EmbryoCV, Embryo Computer Vision.
(DOCX)

S4 Code ImageJ macro used for recording beat-to-beat timings in the cardiac activity of the amphipod *O*. *gammarellus*. (DOCX)

S1 Video Time-lapse and real-time video of *R*. *balthica* embryos developing in 20°C and 25°C acquired using OpenVIM. OpenVIM, Open-source Video Microscope.
(MP4)

S1 Table Guide to OpenVIM components. OpenVIM, Open-source Video Microscope.
(PDF)

S1 Text Assembly guide for OpenVIM. OpenVIM, Open-source Video Microscope.
(PDF)

S1 Data Comparison of manually and EmbryoCV-performed measurements of temporal and spatial phenome-level traits. EmbryoCV, Embryo Computer Vision.
(CSV)

S2 Data Growth rate and mean distance moved during each recording and mean heart rate of *R*. *balthica* embryos from first cell division until hatching at 20°C, 25°C, and 30°C, as measured using EmbryoCV. EmbryoCV, Embryo Computer Vision.
(CSV)

S3 Data Proportion of alive and dead E3-, E7-, and E9-stage *R*. *balthica* embryos during a 24 h period at 36°C, as measured using EmbryoCV. EmbryoCV, Embryo Computer Vision.
(CSV)

S4 Data Growth rate and mean distance moved during each recording in E3-stage *R*. *balthica* embryos during a 24 h exposure to 30°C and a salinity of 0 or 7. (CSV)

S5 Data Heart rate following first heart function and rate of subsequent change in *O*. *gammarellus* cultured at 15°C and 20°C for 24 h, as measured using EmbryoCV. EmbryoCV, Embryo Computer Vision. (CSV)

## Abbreviations

APW: artificial pond water
EmbryoCV: Embryo Computer Vision
IVF: in vitro fertilisation
LT: lethal time
OpenSPIM: Open Selective Plane Illumination Microscopy
OpenVIM: Open-source Video Microscope
