# Supplementary material for: A high-throughput and open-source platform for embryo phenomics
Source: PLoS Biol. 2018 Dec 13;16(12):e3000074. doi: 10.1371/journal.pbio.3000074 (PMC6292576; doi:10.1371/journal.pbio.3000074)
Supplement: S3 Code — Interface provides users ability to measure and record embryo (polygon tool) and egg outlines (ellipse), with automated saving of results, for comparison with EmbryoCV-determined measures. EmbryoCV, Embryo Computer Vision. (DOCX) [file pbio.3000074.s003.docx]

**Supplementary 3. ImageJ ActionBar macro used for manual analysis of phenotypic traits.**

// Action Bar description file :manual_validation

run("Action Bar","/plugins/ActionBar/manual_validation.txt");

exit;

<onTop>

<startupAction>

var measure = "notSet";

</startupAction>

//<line>

<DnDAction>

file=getArgument();

savedir = "/Users/mberdevbio4/Dropbox/manualMeasurements/";

call("ij.Prefs.set", "validate.savedir",savedir);

open(file);

run("8-bit");

</DnDAction>

//</line>

// Embryo XY coordinates

<line>

<button> 1 line 1

label=Embryo Outline

icon=noicon

//manual_validation/image1_1.png

arg=<macro>

dir = getDirectory("image");

call("ij.Prefs.set", "validate.dir",dir);

name=getTitle;

call("ij.Prefs.set", "validate.name",name);

setTool("polygon");

measure = "embryo";

call("ij.Prefs.set", "validate.measure",measure);

</macro>

// Egg ellipse

<button> 2 line 1

label=Egg Ellipse

icon=noicon

arg=<macro>

dir = getDirectory("image");

call("ij.Prefs.set", "validate.dir",dir);

name=getTitle;

call("ij.Prefs.set", "validate.name",name);

setTool("ellipse");

run("Set Measurements...", "centroid fit redirect=None decimal=3");

measure = "egg";

call("ij.Prefs.set", "validate.measure",measure);

</macro>

<button> 3 line 1

label=PulseToPulse

icon=noicon

arg=<macro>

dir = getDirectory("image");

call("ij.Prefs.set", "validate.dir",dir);

name=getTitle;

call("ij.Prefs.set", "validate.name",name);

measure = "pulsetopulse";

call("ij.Prefs.set", "validate.measure",measure);

</macro>

// Show IJ

<button> 4 line 1

label=Show/Hide IJ

icon=noicon

arg=<hide>

</macro>

</line>

<line>

<button> 5 line 2

label=Save Measurement

icon = noicon

arg=<macro>

measure = call("ij.Prefs.get", "validate.measure",measure);

name = call("ij.Prefs.get", "validate.name",name);

dir = call("ij.Prefs.get", "validate.dir",dir);

savedir = call("ij.Prefs.get", "validate.savedir",savedir);

if (measure == "egg"){

run("Clear Results");

run("Measure");

saveAs("Results", savedir + getInfo("slice.label") + "_eggEllipse_results.txt");

selectWindow("Results");

run("Close");

}

if (measure == "embryo"){

saveAs("XY Coordinates", savedir + getInfo("slice.label") + "_embryoXY_results.txt");

}

if (measure != "embryo" && measure !="egg"){

print('Make measurement before attempting to save');

}

</macro>

</line>

<text> Drop an image file here

//<sticky>

// end of file

**Appendix 4. ImageJ macro used for recording beat to beat timings in cardiac activity.**

macro "InstanceRecord [i]" {

print(getTitle());

id=getImageID();

lastSlice=-2;

while(true) {

slice = getSliceNumber();

if(isKeyDown("space") && slice>(lastSlice + 2)) {

if(getImageID()!=id) exit("different image, recording of frames stopped");

print(slice); beep();

lastSlice=slice;

}

wait(100);

}

**Appendix 5. EmbryoCV Python Class, containing the components: EmbryoCV.py, dataHandling.py, imageAnalysis.py, dataIntegration.py, dataAnalysis.py, eggUI.py.**

**EmbryoCV.py**

# Import dependencies

import pandas as pd

import os

from imageAnalysis import imageAnalysis

from dataHandling import dataHandling

from dataAnalysis import dataAnalysis

from xarrayDataAnalysis import xarrayDataAnalysis

from dataIntegration import dataIntegration

import warnings

#from collapsedDataAnalysis import collapsedDataAnalysis

class EmbryoCV(imageAnalysis, dataHandling, dataAnalysis, xarrayDataAnalysis, dataIntegration):

'''''

- embryoCV class(parentPath, new = 'no')

> Initialise with two argments parent path i.e. the folder containing the sequentially

labelled MicroManager folders for each time point

/Volumes/DataDrive/Time_1/Position1

................../Time_1/Position2

................../Time_2/Position1

................../Time_2/Position2

parentPath = '/Volumes/DataDrive/'

> Second argument specifies mode:

- 'new' = a new analysis - build results files and locate eggs

- 'resume' = load a previous analysis.

- 'results' = load a previous analysis, but just from the results folder,

(i.e. the results folder is seperate from the original images)

- 'xarray' = load an XArray version of the results. This is encouraged over

summaryresults below as it is fast (data is accessed dynamically from disk)

and it contains all of the results data in a format that can be easily added to

with subsequent analysis.

- 'summaryresults' = load a reduced version of the results (no embryo

outlines or blockwise/freq data) from a numpy dict.

- filterResultsList(fileSize)

A function to generate a size filtered list of results files from the

results directory. Can be used if some of the results files are incomplete,

owing to either a failed or prematurely aborted analysis. Provide the

file size to filter by in bytes.

- seqImport(seqPath)

Import a continuous sequence of images from specified dirctory. Images

are converted from 16 bits to 8 bits, scaled and assigned to 'seq', a numpy

array.

- imImport(n,m)

Import a single image using 'currentFolder'and 'filename' taken from the

currently loaded results . n specifies the results panel and m the sequence

number.

- getEmbryoLabels(parentPath)

Generate a list of embryo labels from the specified parent path. The embryo

list is assigned to embryoLabels.

- getEmbryoLabelsFromResultsFolder(resultsDir)

Use the results files in the results directory to generate a list of embryo

labels. These are assigned to embryoLabels.

- getEmbryoFolders(parentPath, embryo)

Generate a list of folders containing image sequences for a particular embryo.

The embryo folder list is assigned to embryoFolders and is ordered by

date created.

- createResultsFolder()

Generate a 'phenomeData' results folder in the parentPath.

- generateResultsAndFindEggs(parentPath, scale, eggInt=1234):

Generate results files for each embryo

'''''

#==============================================================================

#==============================================================================

def __init__(self,parentPath,mode='unspecified', scale='na',exclude = 'na',species='rbalthica', dataformat=True):

if mode == 'resume':

self.mode = mode

self.dataformat = dataformat

self.parentPath = parentPath

self.exclude = exclude

if (species == 'rbalthica') or (species =='ogammarellus'):

self.species = species

else:

print '** ' + str(species) + ' not currently supported' + ' **'

self.getEmbryoLabels(self.parentPath)

print len(self.embryoLabels), 'embryos identified'

self.resultsDir = os.path.dirname(self.parentPath + "phenomeData/")

self.loadMetadata()

self.embryo = self.embryoLabels[0]

#self.loadResults()

#self.getEmbryoFolders(self.parentPath, self.embryo)

print 'EmbryoCV launched in resume mode. Resume analysis at appropriate stage'

elif mode == 'new':

self.mode = mode

self.dataformat = dataformat

self.parentPath = parentPath

self.resultsDir = os.path.dirname(self.parentPath + "phenomeData/")

self.exclude = exclude

if (species == 'rbalthica') or (species =='ogammarellus'):

self.species = species

else:

print '** ' + str(species) + ' not currently supported' + ' **'

self.getEmbryoLabels(self.parentPath)

self.embryo = self.embryoLabels[0]

if scale != 'na':

self.scale = scale

print 'New instance of EmbryoCV launched.'

print len(self.embryoLabels), 'embryos identified'

# If R.balthica - generate results table and locate eggs..

if self.species == 'rbalthica':

print 'Identifying eggs and generating results tables'

# Non-parallel version - useful for debugging..

# self.generateResultsAndFindEggs(self.parentPath,self.scale)

self.parallelGenerateResultsAndFindEggs(self.parentPath,self.scale)

# If O. gammarellus generate results table, but dont locate eggs

# as egg and embryo are indistinguishable from each other, so diff.

# approach required.

if self.species == 'ogammarellus':

self.generateResults(self.parentPath,self.scale)

else:

print 'Warning: scale not given!'

elif mode == 'results':

self.mode = mode

self.dataformat = dataformat

self.parentPath = parentPath

self.resultsDir = self.parentPath

self.exclude = exclude

if (species == 'rbalthica') or (species =='ogammarellus'):

self.species = species

else:

print '** ' + str(species) + ' not currently supported' + ' **'

self.getEmbryoLabelsFromResultsFolder(self.parentPath)

self.loadMetadata()

if (species == 'rbalthica') or (species =='ogammarellus'):

self.species = species

else:

print '** ' + str(species) + ' not currently supported' + ' **'

print len(self.embryoLabels), 'embryos identified'

self.resultsDir = os.path.dirname(self.parentPath)

# Depreciated..

#elif mode == 'summaryresults':

# self.mode = mode

# self.parentPath = parentPath

# self.collapsedAnalysis = collapsedDataAnalysis()

# self.collapsedAnalysis.parentPath = self.parentPath

elif mode == 'xarray':

self.mode = mode

self.exclude = exclude

self.parentPath = parentPath

self.resultsDir = self.parentPath

self.dataformat = dataformat

if (species == 'rbalthica') or (species =='ogammarellus'):

self.species = species

else:

print '** ' + str(species) + ' not currently supported' + ' **'

self.getXREmbryoLabelsFromResultsFolder(self.parentPath)

self.loadMetadata()

print len(self.embryoLabels), 'embryos identified'

self.xarray = xarrayDataAnalysis()

#self.xarray.

parentPath = self.parentPath

else:

print 'Invalid mode argument (resume, new or results).'

# Surpress warning regarding ctypes.

warnings.filterwarnings('ignore',category=pd.io.pytables.PerformanceWarning)

**dataHandling.py**

# Import dependencies

import cv2

import numpy as np

import pandas as pd

import glob

import os

import sys

import eggUI

import viewOutput

import time

import pathos

import json

import xarray as xr

from PyQt5.Qt import *

from PyQt5 import QtGui

class dataHandling(object):

#==============================================================================

# Produce a list of embryos with results fileslarger than a particular size.

#==============================================================================

def filterResultsList(self,fileSize):

#self.resultsDir = os.path.dirname(self.parentPath + "phenomeData/")

self.sizeFilteredEmbryos = []

for f in range(len(self.embryoLabels)):

self.embryo = self.embryoLabels[f]

resultsStructure = self.resultsDir +'/' + self.embryo + '.pandas'

size = os.path.getsize(resultsStructure)/1000000

if size >= fileSize:

self.sizeFilteredEmbryos.append(self.embryo)

#==============================================================================

# Function to identify the egg

#==============================================================================

def seqImport(self, n):

self.seq = np.zeros(shape=(self.results.shape[1],self.imImport(0,0).shape[0],self.imImport(0,0).shape[1]))

for f in range(self.results.shape[1]):

self.seq[f] = cv2.imread(self.results.iloc[n]['currentFolder'][f] + self.results.iloc[n]['file'][f],cv2.IMREAD_ANYDEPTH)

ran = (self.seq.max() - self.seq.min()) / 255.

self.seq = self.seq/ran

self.seq = self.seq-self.seq.min()

self.seq = np.ascontiguousarray(self.seq.astype(np.uint8))

#np.ascontiguousarray

#==============================================================================

# Import single image using results

#==============================================================================

def imImport(self,n,m):

im = cv2.imread(self.results.iloc[n]['currentFolder'][m] + self.results.iloc[n]['file'][m],cv2.IMREAD_ANYDEPTH)

ran = (im.max()-im.min())/255.

out = (im/ran)

out = out-out.min()

out = out.astype(np.uint8)

return out

#==============================================================================

# Get embryo labels from a parentPath

#==============================================================================

def getEmbryoLabels(self,parentPath):

folders = glob.glob(parentPath + "*/*/")

# Trouble with getctime .. doesn't seem well supported. Therefore switched.

folders.sort(key=os.path.getmtime)

if len(folders)<1:

print 'Data not found.'

embryoLabels = []

for p in range(len(folders)):

embryoLabels.append(os.path.basename(os.path.normpath((folders[p]))))

if self.species == 'rbalthica':

# Reduce to only the unique labels

embryoLabels = np.unique(embryoLabels)

self.embryoLabels = embryoLabels[embryoLabels != 'BLANK']

# Use to remove 'problematic embryos'

if self.exclude is not 'na':

if type(self.exclude) is str:

self.embryoLabels = self.embryoLabels[self.embryoLabels != self.exclude]

else:

self.embryoLabels = self.embryoLabels

for p in range(len(self.exclude)):

self.embryoLabels = self.embryoLabels[self.embryoLabels != str(self.exclude[p])]

elif self.species == 'ogammarellus':

# Add a filter to deal with 'copied data - such as Orchestia methods MS data'

labs = []

for p in range(len(folders)):

labs.append(str(os.path.basename(os.path.normpath((folders[p])))).split(' ')[0])

# Reduce to only the unique labels

embryoLabels = np.unique(labs)

self.embryoLabels = embryoLabels[embryoLabels != 'BLANK']

#==============================================================================

# Get embryo labels from a parentPath

#==============================================================================

def getEmbryoLabelsFromResultsFolder(self,resultsDir):

if self.dataformat:

folders = glob.glob(resultsDir + "/*.pandas")

embryoLabels = []

resultsDir +"/"

for f in range(len(folders)):

embryoLabels.append(folders[f].replace(".pandas", ""))

#embryoLabels[f] = embryoLabels[f].replace(resultsDir +"/","")

embryoLabels[f] = embryoLabels[f].replace(resultsDir,"")

# Reduce to only the unique labels (shouldn't be a problem as from results).

embryoLabels = np.unique(embryoLabels)

self.embryoLabels = embryoLabels[embryoLabels != 'BLANK']

# Use to remove 'problematic embryos'

if self.exclude is not 'na':

if type(self.exclude) is str:

self.embryoLabels = self.embryoLabels[self.embryoLabels != self.exclude]

else:

self.embryoLabels = self.embryoLabels

for p in range(len(self.exclude)):

self.embryoLabels = self.embryoLabels[self.embryoLabels != str(self.exclude[p])]

else:

# If raw does not equal True (i.e. if data do not follow the typical raw MicroManager format

# due perhaps to being copied from elesewhere).

# For example Parent Folder/EmbryoA/Time1

# /Time2

# /EmbryoB/Time1

# /Time2

folders = glob.glob(resultsDir + "/*.pandas")

embryoLabels = []

resultsDir +"/"

for f in range(len(folders)):

embryoLabels.append(folders[f].replace(".pandas", ""))

embryoLabels[f] = embryoLabels[f].replace(resultsDir +"/","")

# Reduce to only the unique labels (shouldn't be a problem as from results).

embryoLabels = np.unique(embryoLabels)

self.embryoLabels = embryoLabels[embryoLabels != 'BLANK']

# Debug

print self.embryoLabels

# Debug

# print self.embryoLabels

#==============================================================================

# Get embryo labels from a parentPath with XARRAY data

#==============================================================================

def getXREmbryoLabelsFromResultsFolder(self,resultsDir):

# If data in normal MM format..

folders = glob.glob(resultsDir + "/*.HDF5")

embryoLabels = []

resultsDir +"/"

for f in range(len(folders)):

embryoLabels.append(folders[f].replace(".HDF5", ""))

embryoLabels.append(folders[f].replace("dataset", ""))

embryoLabels[f] = embryoLabels[f].replace(resultsDir +"/","")

# Reduce to only the unique labels (shouldn't be a problem as from results).

embryoLabels = np.unique(embryoLabels)

self.embryoLabels = embryoLabels[embryoLabels != 'BLANK']

# Debug

# print self.embryoLabels

#==============================================================================

# Get folders for a particular embryo

#==============================================================================

def getEmbryoFolders(self, parentPath, embryo):

if self.mode == 'results':

self.parentPath = parentPath

self.embryo = embryo

self.embryoFolders = self.results.ix[:,0,'currentFolder']

self.getShortenedPaths()

if (self.mode == 'resume'):

self.parentPath = parentPath

self.embryo = embryo

if self.species == 'rbalthica':

#self.embryoFolders = self.results.items

# Oli changed (1211) to deal with corrupted HD (2017018)

#self.embryoFolders = glob.glob(parentPath + "*/" + embryo +"/")

self.embryoFolders = self.results.ix[:,0,'currentFolder']

self.embryoFolders = list(self.embryoFolders)

self.embryoFolders.sort(key=os.path.getmtime)

#self.embryoFolders.sort(key=os.path.getmtime)

self.getShortenedPaths()

elif self.species == 'ogammarellus':

self.embryoFolders = self.results.items

self.embryoFolders = glob.glob(parentPath + "*/" + embryo +" */")

self.embryoFolders.sort(key=os.path.getmtime)

self.getShortenedPaths()

if (self.mode == 'new'):

if self.species == 'rbalthica':

self.parentPath = parentPath

self.embryo = embryo

self.embryoFolders = glob.glob(parentPath + "*/" + embryo +"/")

self.embryoFolders.sort(key=os.path.getmtime)

self.getShortenedPaths()

elif self.species == 'ogammarellus':

#self.embryoFolders = self.results.items

self.embryoFolders = glob.glob(parentPath + "*/" + embryo +" */")

self.embryoFolders.sort(key=os.path.getmtime)

self.getShortenedPaths()

#==============================================================================

# Get list of shortenedPaths

#==============================================================================

def getShortenedPaths(self):

if (self.mode == 'results')|(self.mode == 'resume'):

self.shortenedPaths = self.results.items

if self.mode == 'new':

self.shortenedPaths=[]

for f in range(len(self.embryoFolders)):

self.shortenedPaths.append(os.path.relpath(self.embryoFolders[f], self.parentPath))

#==============================================================================

# Create a directory for saving results

#==============================================================================

def createResultsFolder(self):

self.resultsDir = os.path.dirname(self.parentPath + "phenomeData/")

if not os.path.exists(self.resultsDir):

os.makedirs(self.resultsDir)

#==============================================================================

# Generate a results panel suitable for experiment AND then Run egg ID

#==============================================================================

def generateResultsAndFindEggs(self, parentPath, scale, eggInt=1234):

self.eggInt = eggInt

totts = time.time()

self.compiledData = {}

self.parentPath = parentPath

self.scale = scale

self.getEmbryoLabels(self.parentPath)

self.createResultsFolder()

for e in range(len(self.embryoLabels)):

ts = time.time()

print 'Initiation started for', self.embryoLabels[e]

self.getEmbryoFolders(self.parentPath,self.embryoLabels[e])

for f in range(len(self.embryoFolders)):

print f, self.embryoFolders[f]

self.currentFolder = self.embryoFolders[f]

self.shortenedPath = self.shortenedPaths[f]

self.parseMetadata()

self.createResultsTable()

self.compiledData[self.shortenedPath] = self.results

print 'Initiation complete for', self.embryoLabels[e], ('in {} s'.format(time.time()-ts))

self.resultSheets = pd.Panel.from_dict(self.compiledData)

self.resultSheets.to_pickle(self.resultsDir + "/" + self.embryo + '.pandas')

self.results=[]

self.resultSheets=[]

self.compiledData={}

self.saveMetadata()

self.runEggID(self.eggInt)

print 'Egg identification and creation of results files is now complete. '

print 'This took ', ('{} s'.format(time.time()-totts))

#==============================================================================

# Just generate results - route for O. gammarellus analysis. Initiated upon creating

# a 'new' experiment.

#

# Generate a results panel suitable for experiment AND then Run egg ID

#==============================================================================

def generateResults(self, parentPath, scale):

totts = time.time()

self.compiledData = {}

self.parentPath = parentPath

self.scale = scale

self.getEmbryoLabels(self.parentPath)

self.createResultsFolder()

for e in range(len(self.embryoLabels)):

ts = time.time()

print 'Initiation started for', self.embryoLabels[e]

self.getEmbryoFolders(self.parentPath,self.embryoLabels[e])

for f in range(len(self.embryoFolders)):

print f, self.embryoFolders[f]

self.currentFolder = self.embryoFolders[f]

self.shortenedPath = self.shortenedPaths[f]

self.parseMetadata()

self.createResultsTable()

self.compiledData[self.shortenedPath] = self.results

print 'Results file created for ', self.embryoLabels[e], ('in {} s'.format(time.time()-ts))

self.resultSheets = pd.Panel.from_dict(self.compiledData)

self.resultSheets.to_pickle(self.resultsDir + "/" + self.embryo + '.pandas')

self.results=[]

self.resultSheets=[]

self.compiledData={}

self.saveMetadata()

#self.runEggID(self.eggInt)

print 'This took ', ('{} s'.format(time.time()-totts))

print 'Egg identification and creation of results files is now complete. Use instance.analyseAllEmbryos function to continue'

#==============================================================================

# Create results structure

#==============================================================================

def createResultsTable(self):

global da

# Store embryo outline as numpy array, but results as panda.

cols = ['embryo','currentFolder','parentPath','file','scale','UUID','dateTime','elapsedTime',

'area','centroidX','centroidY','solidity','aspect','extent',

'hullArea','bboxMincol','bboxMinrow','bboxWidth','bboxHeight', 'embryoOutline'

,'eggRotBBox','eggBoxPoints', 'blockWise']

#, 'eggBBox'

self.results = pd.DataFrame(index = range(int(len(self.filtFiles))), columns = cols)

self.results.embryo = self.embryo

self.results.currentFolder = self.currentFolder

self.results.file = self.filtFiles

self.results.parentPath = self.parentPath

self.results.scale = self.scale

self.results.dateTime = self.filtTimes

self.results.elapsedTime = self.filtElapsedTimes

self.results.UUID = self.filtUUID

#==============================================================================

# Load metadata

#==============================================================================

def parseMetadata(self):

# Search currentFolder for metadata

with open(glob.glob(self.currentFolder + "/*.txt")[0], 'r') as f:

jsonDict = json.loads(f.read())

times = []

elapsedTimes = []

files = []

UUID = []

for i in jsonDict.keys():

if i!=u'Summary':

times.append(jsonDict[i]['Time'])

elapsedTimes.append(jsonDict[i]['ElapsedTime-ms'])

files.append(jsonDict[i]['FileName'])

UUID.append(jsonDict[i]['UUID'])

meta = [(y,x,z,a) for (y,x,z,a) in sorted(zip(times,elapsedTimes,files,UUID))]

self.filtTimes = []

self.filtElapsedTimes = []

self.filtFiles = []

self.filtUUID = []

for i in range(len(meta)):

self.filtTimes.append(str(meta[i][0]))

self.filtElapsedTimes.append(str(meta[i][1]))

self.filtFiles.append(str(meta[i][2]))

self.filtUUID.append(str(meta[i][3]))

#==============================================================================

# Apply locateEgg to image sequence at specified interval

#==============================================================================

def getIntervIndicesFromSequence(self, n):

# n = Specifies how many frames to skip in between the locateEgg.

#self.intN = self.results.shape[1]-1

self.intN = n

self.eggIDIms = []

# If n = 1234 (default), only the first image from each sequence is taken.

if self.intN == 1234:

self.eggIDIms.append(0)

# If an interval is specified...

else:

seqLength = self.results.shape[1]

# Get number of IDs necessary, based on n and seqLength.

eggIDNum = seqLength/n

# Get frame indices

for i in range(eggIDNum):

self.eggIDIms.append(i*self.intN)

#==============================================================================

# Import a non continuous sequence

#==============================================================================

def nonContSeqImport(self, f):

# Create np stack of appropriate size

im = self.imImport(0,0)

self.seq = np.zeros(shape=(int(len(self.eggIDIms)),im.shape[0],im.shape[1]))

# Loop over the eggIDIms

for g in range(len(self.eggIDIms)):

# Import image one by one, from correct folder (argument) and iterated eggIDIm.

self.seq[g] = self.imImport(f,self.eggIDIms[g])

self.seq = self.seq.astype(np.uint8)

#==============================================================================

# Load results for current embryo

#==============================================================================

def loadResults(self):

# Iterate over embryo labels...

resultsStructure = self.resultsDir +'/' + self.embryo + '.pandas'

results = pd.read_pickle(resultsStructure)

# Reorder panels so they are chronological, using the first datetime from each panel.

dates = pd.to_datetime(results.ix[:,0,6])

# Sort them

dates.sort_values(inplace=True)

# Reindex axis

self.results = results.reindex_axis(dates.index, copy='False')

#==============================================================================

# Load results for current embryo - XARRAY

#==============================================================================

def loadXRResults(self):

# Iterate over embryo labels...

if self.mode is 'xarray':

if float(str(glob.glob(self.parentPath + self.embryo + "dataset*")[0]).find('log')) > 0:

self.results = 'NoData'

else:

self.results = xr.open_mfdataset(self.parentPath + self.embryo + 'dataset.HDF5')

#==============================================================================

# Load, but return results

#==============================================================================

def returnResults(self, embryo):

# Iterate over embryo labels...

resultsStructure = self.resultsDir +'/' + embryo + '.pandas'

results = pd.read_pickle(resultsStructure)

# Reorder panels so they are chronological, using the first datetime from each panel.

dates = pd.to_datetime(results.ix[:,0,6])

# Sort them

dates.sort_values(inplace=True)

# Reindex axis

results = results.reindex_axis(dates.index, copy='False')

return results

#==============================================================================

# Apply egg ID

#==============================================================================

# Run egg ID and add to the results table before saving

# If no eggInt given then just run on the first image of each sequence

def runEggID(self, eggInt = 1234):

self.eggInt = eggInt

for e in range(len(self.embryoLabels)):

self.embryo = self.embryoLabels[e]

self.getEmbryoFolders(self.parentPath, self.embryo)

self.loadResults()

#if self.eggInt ==1234:

#self.eggIDIms = []

#self.eggIDIms.append(0)

self.getIntervIndicesFromSequence(int(self.eggInt))

for f in range(len(self.embryoFolders)):

print self.embryoFolders[f]

self.nonContSeqImport(f)

for g in range(len(self.eggIDIms)):

# If g = [0] i.e. eggInt = 1234, make g = 0.

if len(self.eggIDIms) == 1:

g =0

# NOW perform the eggID on each intervalled image

self.locateEgg(self.seq[g])

# AND store eggID output in results table

#self.results.ix[f,self.eggIDIms[g],'eggBBox'] = self.eggBBox

self.results.ix[f,self.eggIDIms[g],'eggRotBBox'] = self.eggRotBBox

self.results.ix[f,self.eggIDIms[g],'eggBoxPoints'] = self.boxPoints

self.results.to_pickle(self.resultsDir + "/" + self.embryo + '.pandas')

#==============================================================================

# Fill missing egg measurements with ones from either earlier or later time points

# in the data panel series. Note: This is a suboptimal solution and should be

# avoided via manual checking using eggUI.

#==============================================================================

def fillEggMeasurements(self):

# Fill non calculated values frames from most nrecent previously calculated.

for f in range(self.results.shape[0]):

#self.results.ix[f,:,'eggBBox'] = self.results.ix[f,:,'eggBBox'].fillna(method = 'ffill')

#self.results.ix[f,:,'eggBBox'] = self.results.ix[f,:,'eggBBox'].fillna(method = 'bfill')

self.results.ix[f,:,'eggBoxPoints'] = self.results.ix[f,:,'eggBoxPoints'].fillna(method = 'ffill')

self.results.ix[f,:,'eggBoxPoints'] = self.results.ix[f,:,'eggBoxPoints'].fillna(method = 'bfill')

self.results.ix[f,:,'eggRotBBox'] = self.results.ix[f,:,'eggRotBBox'].fillna(method = 'ffill')

self.results.ix[f,:,'eggRotBBox'] = self.results.ix[f,:,'eggRotBBox'].fillna(method = 'bfill')

self.results.ix[f,:,'bboxMincol'] = self.results.ix[f,:,'bboxMincol'].fillna(method = 'ffill')

self.results.ix[f,:,'bboxMinrow'] = self.results.ix[f,:,'bboxMinrow'].fillna(method = 'bfill')

# If no value in first datapoint in first panel..

for f in range(self.results.shape[0]):

null = self.results.ix[:,0,'eggBoxPoints'].isnull()

if null[f]:

for g in range(len(null)-f):

if ~null[(f+g)]:

#self.results.ix[f,0,'eggBBox'] = self.results.ix[(f+g),0,'eggBBox']

#self.results.ix[f,:,'eggBBox'] = self.results.ix[f,:,'eggBBox'].fillna(method = 'ffill')

self.results.ix[f,0,'eggBoxPoints'] = self.results.ix[(f+g),0,'eggBoxPoints']

self.results.ix[f,:,'eggBoxPoints'] = self.results.ix[f,:,'eggBoxPoints'].fillna(method = 'ffill')

self.results.ix[f,0,'eggRotBBox'] = self.results.ix[(f+g),0,'eggRotBBox']

self.results.ix[f,:,'eggRotBBox'] = self.results.ix[f,:,'eggRotBBox'].fillna(method = 'ffill')

self.results.ix[f,0,'bboxMincol'] = self.results.ix[(f+g),0,'bboxMincol']

self.results.ix[f,:,'bboxMincol'] = self.results.ix[f,:,'bboxMincol'].fillna(method = 'ffill')

self.results.ix[f,0,'bboxMinrow'] = self.results.ix[(f+g),0,'bboxMinrow']

self.results.ix[f,:,'bboxMinrow'] = self.results.ix[f,:,'bboxMinrow'].fillna(method = 'ffill')

break

# Now repeat process but filling later panels in the dataframe with data from earlier panels.

for f in range(self.results.shape[0]):

null = self.results.ix[:,0,'eggBoxPoints'].isnull()

if null[f]:

#print f

for g in range(f,0,-1):

if ~null[g]:

#self.results.ix[f,0,'eggBBox'] = self.results.ix[g,0,'eggBBox']

#self.results.ix[f,:,'eggBBox'] = self.results.ix[f,:,'eggBBox'].fillna(method = 'ffill')

self.results.ix[f,0,'eggBoxPoints'] = self.results.ix[g,0,'eggBoxPoints']

self.results.ix[f,:,'eggBoxPoints'] = self.results.ix[f,:,'eggBoxPoints'].fillna(method = 'ffill')

self.results.ix[f,0,'eggRotBBox'] = self.results.ix[g,0,'eggRotBBox']

self.results.ix[f,:,'eggRotBBox'] = self.results.ix[f,:,'eggRotBBox'].fillna(method = 'ffill')

self.results.ix[f,0,'bboxMincol'] = self.results.ix[g,0,'bboxMincol']

self.results.ix[f,:,'bboxMincol'] = self.results.ix[f,:,'bboxMincol'].fillna(method = 'ffill')

self.results.ix[f,0,'bboxMinrow'] = self.results.ix[g,0,'bboxMinrow']

self.results.ix[f,:,'bboxMinrow'] = self.results.ix[f,:,'bboxMinrow'].fillna(method = 'ffill')

break

#==============================================================================

# Apply embryo segmentation to all embryos

#==============================================================================

def segmentAllEmbryos(self):

for e in range(len(self.embryoLabels)):

print 'Starting segmentation of', self.embryoLabels[e]

# Make embyo label current (for saving)

self.embryo = self.embryoLabels[e]

self.loadResults()

# Interpolate egg measurements to whole dataframe

self.fillEggMeasurements()

self.getEmbryoFolders(self.parentPath,self.embryoLabels[e])

self.runEmbryoSegmentation()

print 'Finished segmentation of', self.embryoLabels[e]

#==============================================================================

# Apply embryo segmentation to specific embryo

#==============================================================================

def segmentSpecificEmbryos(self, embryo):

# Make embyo label current (for saving)

if isinstance(embryo, str):

self.embryo = embryo

self.loadResults()

self.fillEggMeasurements()

self.getEmbryoFolders(self.parentPath,self.embryo)

self.runEmbryoSegmentation()

print self.embryo, 'segmentation complete'

if isinstance(embryo, list):

for e in range(len(embryo)):

self.embryo = embryo[e]

self.loadResults()

self.getEmbryoFolders(self.parentPath,self.embryo)

self.runEmbryoSegmentation()

print self.embryo, 'segmentation complete'

#==============================================================================

# Worker function for parallel embryo segmentation

#==============================================================================

def parallelSegmentAllEmbryos(self,e):

print 'Analysis started for:', self.embryoLabels[e]

# Make embyo label current (for saving)

ts = time.time()

self.embryo = self.embryoLabels[e]

self.loadResults()

if self.species == 'rbalthica':

# Interpolate egg measurements to whole dataframe

self.fillEggMeasurements()

self.getEmbryoFolders(self.parentPath,self.embryoLabels[e])

self.runParEmbryoSegmentation()

print 'Analysis complete for', self.embryoLabels[e], ('in {} s'.format(time.time()-ts))

#==============================================================================

# Multiprocessing parallel embryo segmetnation function

#==============================================================================

def quantifyAllEmbryos(self,par=True,exclude='na'):

# Exclude any embryos that user does not want to be analysed.

if exclude is not 'na':

if type(exclude) is str:

self.embryoLabels = self.embryoLabels[self.embryoLabels != exclude]

else:

for p in range(len(exclude)):

self.embryoLabels = self.embryoLabels[self.embryoLabels != str(exclude[p])]

if par is True:

# Four seems a good compromise for processing vs data IO on a 12 core

# MacPro, with data on a 7200 RPM SATA, connected via USB3.0/eSATA or Thunderbolt.

cpuCount = 4

# Uncomment if you want to maximise cpu usage, note that data IO from

# drives will likely become limiting and cause serious issues..

#cpuCount = pathos.multiprocessing.cpu_count()

self.getEmbryoLabels(self.parentPath)

jobSize= len(self.embryoLabels)

jobRange= range(len(self.embryoLabels))

if cpuCount > len(self.embryoLabels):

pool = pathos.multiprocessing.ProcessPool(jobSize)

pool.map(self.parallelSegmentAllEmbryos, jobRange)

else:

pool = pathos.multiprocessing.ProcessPool(cpuCount)

pool.map(self.parallelSegmentAllEmbryos, jobRange)

else:

# If par is not True then use non parallel version.

self.segmentAllEmbryos()

#==============================================================================

# Save metadata

#==============================================================================

def saveMetadata(self):

if self.species == 'rbalthica':

metadata = {'embryoLabels':self.embryoLabels, 'scale':self.scale, 'eggInt':self.eggInt}

elif self.species == 'ogammarellus':

metadata = {'embryoLabels':self.embryoLabels, 'scale':self.scale, 'eggInt':1234}

np.save(self.resultsDir + "/phenomeMetadata", metadata)

#==============================================================================

# Load metadata

#==============================================================================

def loadMetadata(self):

self.metadata = np.load(self.resultsDir + "/phenomeMetadata.npy")

self.scale = self.metadata[()]['scale']

self.embryoLabels = self.metadata[()]['embryoLabels']

# Use to remove 'problematic embryos'

if self.exclude is not 'na':

if type(self.exclude) is str:

self.embryoLabels = self.embryoLabels[self.embryoLabels != self.exclude]

else:

self.embryoLabels = self.embryoLabels

for p in range(len(self.exclude)):

self.embryoLabels = self.embryoLabels[self.embryoLabels != str(self.exclude[p])]

#==============================================================================

# Multiprocessing parallel embryo segmetnation functions

#==============================================================================

# Performs analysis for each embryo, looping over embryoFolders.

def initEmParallel(self,e):

print 'Initiation in progress for ', self.embryoLabels[e]

# Make embyo label current (for saving)

#ts = time.time()

self.getEmbryoFolders(self.parentPath,self.embryoLabels[e])

self.embryo = self.embryoLabels[e]

for f in range(len(self.embryoFolders)):

self.currentFolder = self.embryoFolders[f]

self.shortenedPath = self.shortenedPaths[f]

#self.shortenedPath = os.path.relpath(self.currentFolder, self.parentPath)

self.parseMetadata()

self.createResultsTable()

self.compiledData[self.shortenedPath] = self.results

self.resultSheets = pd.Panel.from_dict(self.compiledData)

self.resultSheets.to_pickle(self.resultsDir + "/" + self.embryo + '.pandas')

self.results=[]

self.resultSheets=[]

self.compiledData={}

# Now the results file is created and saved open it and populate with egg measurements

self.loadResults()

self.getIntervIndicesFromSequence(int(self.eggInt))

for f in range(len(self.embryoFolders)):

self.nonContSeqImport(f)

for g in range(len(self.eggIDIms)):

if len(self.eggIDIms) == 1:

g =0

# NOW perform the eggID on each intervalled image

self.locateEgg(self.seq[g])

# AND store eggID output in results table

#self.results.ix[f,self.eggIDIms[g],'eggBBox'] = self.eggBBox

self.results.ix[f,self.eggIDIms[g],'eggRotBBox'] = self.eggRotBBox

self.results.ix[f,self.eggIDIms[g],'eggBoxPoints'] = self.boxPoints

self.results.to_pickle(self.resultsDir + "/" + self.embryo + '.pandas')

# Creates worker pool and allocates jobs.

def parallelInitiation(self):

self.getEmbryoLabels(self.parentPath)

#cpuCount = pathos.multiprocessing.cpu_count()

cpuCount = pathos.multiprocessing.cpu_count()

jobSize= len(self.embryoLabels)

jobRange= range(len(self.embryoLabels))

if cpuCount > len(self.embryoLabels):

pool = pathos.multiprocessing.ProcessPool(jobSize)

pool.map(self.initEmParallel, jobRange)

else:

pool = pathos.multiprocessing.ProcessPool(cpuCount)

pool.map(self.initEmParallel, jobRange)

# Worker function ensuring appropriate functions are called before and after parallel processing.

def parallelGenerateResultsAndFindEggs(self, parentPath, scale, eggInt=1234):

if self.species == 'rbalthica':

totts = time.time()

self.eggInt = eggInt

self.compiledData = {}

self.parentPath = parentPath

self.scale = scale

self.getEmbryoLabels(self.parentPath)

# Debug

print self.embryoLabels

print len(self.embryoLabels)

self.createResultsFolder()

self.parallelInitiation()

self.saveMetadata()

print 'Egg identification and creation of results files is now complete. '

print 'This took ', ('{} s'.format(time.time()-totts))

#==============================================================================

# Return a pandas panel with blockwise and embryooutline removed - can be

# useful for some downstream, restricted analysis.

#==============================================================================

def reduceData(self):

res = self.results.drop('blockWise', axis=2)

res = res.drop('embryoOutline', axis=2)

return res

#==============================================================================

# Save a restricted dataset (excluding blockwise data and embryo outlines

# to a Numpy dictionary.

#==============================================================================

def saveReducedResults(self, savePath):

# Use function and add output to results dict.

reducedResults = {}

for f in range(len(self.embryoLabels)):

self.embryo = self.embryoLabels[f]

self.results = self.returnResults(self.embryo)

reduced = self.reduceData()

# Take embryo label and assign results

#exec('self.%s = reduced' % self.embryo)

print self.embryoLabels[f], 'loaded'

reducedResults[self.embryoLabels[f]] = reduced

# Get an appropriate name to save..

out = self.parentPath.replace('/phenomeData/','')

out = out.split('/')

out = out[len(out)-1]

# Finally save

np.save(savePath + out + '_reducedPhenomeResults.npy', reducedResults)

#==============================================================================

# Functionn to launch Egg ID UI

#==============================================================================

def validateEggs(self, eggInt = 1234):

self.eggInt = eggInt

app = 0

#QtGui.QApplication.setGraphicsSystem('raster')

app = QtGui.QApplication(sys.argv)

self.UI = eggUI.eggUI()

self.dataForUI(0)

self.UI.embryoFolders = self.embryoFolders

self.UI.showUI(self.UI.compSeq, self.results[:,self.eggIDIms,'eggRotBBox'].values, self.results[:,self.eggIDIms,'eggBoxPoints'].values,list(self.embryoLabels), self.eggInt)

#instance1.UI.showUI(instance1.UI.compSeq, instance1.results[:,instance1.eggIDIms,'eggRotBBox'].values, instance1.results[:,instance1.eggIDIms,'eggBoxPoints'].values,list(instance1.embryoLabels), instance1.eggInt)

self.UI.diag.imv.sigTimeChanged.connect(self.UI.updateOpenCVEggROICurrEmbryo)

self.UI.diag.table.itemSelectionChanged.connect(self.supplyUINewEmbryoData)

self.UI.approveROI_btn.clicked.connect(self.saveUpdatedROI)

# Update image when timeline slider is changed.

# self.UI.diag.imv.timeLine.sigPositionChanged.connect(self.UI.updateImage)

app.exec_()

#==============================================================================

# Functions for the validateEggs() user interface - to validate egg locations etc..

#==============================================================================

#==============================================================================

# Load data for the eggUI for a particular embryo

#==============================================================================

def dataForUI(self,e):

# Make embyo label current (for saving)

self.embryo = self.embryoLabels[e]

# Load results

self.loadResults()

self.getEmbryoFolders(self.parentPath, self.embryo)

# If self.eggInt = 1234, only check the first image of each image sequence

# /time series. This is the default. However, users can check very image if

# desired by setting an appropriate eggInt.

if self.eggInt ==1234:

self.eggIDIms = []

self.eggIDIms.append(0)

self.intN = self.results.shape[1]-1

self.getIntervIndicesFromSequence(int(self.eggInt))

else:

# Get indices for loading, based on interval (intN).

self.getIntervIndicesFromSequence(int(self.eggInt))

# Create an empty stack for the egg approval images. Use first im for dimensions

im = self.imImport(0,0)

self.UI.compSeq = np.zeros(shape=(int(len(self.eggIDIms)*len(self.embryoFolders)),im.shape[0],im.shape[1]))

self.UI.eggUIimPaths = []

for e in range(len(self.embryoFolders)):

self.UI.eggUIimPaths[e*len(self.eggIDIms):(e*len(self.eggIDIms)+len(self.eggIDIms))] = self.results.iloc[e]['currentFolder'][self.eggIDIms] + self.results.iloc[e]['file'][self.eggIDIms]

#==============================================================================

# Save changes to egg ROI to disk.

#==============================================================================

def saveUpdatedROI(self):

# Save changes for each embryo's ROI

for r in range(self.UI.eggRotBBox.shape[1]):

self.results.iloc[r]['eggRotBBox'][self.eggIDIms] = self.UI.eggRotBBox[:,r]

for r in range(self.UI.eggBoxPoints.shape[1]):

self.results.iloc[r]['eggBoxPoints'][self.eggIDIms] = self.UI.eggBoxPoints[:,r]

# Store changes to disk

self.results.to_pickle(self.resultsDir + "/" + self.embryo + '.pandas')

#==============================================================================

# When table row selection changes, load new embryo data

#==============================================================================

def supplyUINewEmbryoData(self):

# Debug

# print 'supplyUINewEmbryoData', self.UI.diag.table.currentRow()

currRow = self.UI.diag.table.currentRow()

self.dataForUI(currRow)

self.UI.updateUI(self.UI.compSeq, self.results[:,self.eggIDIms,'eggRotBBox'].values, self.results[:,self.eggIDIms,'eggBoxPoints'].values)

#self.dataforViewOutput(currRow)

#==============================================================================

# View Output functions

#==============================================================================

def viewOutput(self):

app = 0

app = QApplication(sys.argv)

self.outputUI = viewOutput.viewOutput()

self.outputUI.scale = self.scale

self.dataforViewOutput(0)

self.outputUI.showUI(list(self.embryoLabels))

#self.dataForUI(0)

#self.UI.outputUI(self.compSeq, self.results[:,self.eggIDIms,'eggRotBBox'].values, self.results[:,self.eggIDIms,'eggBoxPoints'].values,list(self.embryoLabels))

#test.UI.diag.imv.sigTimeChanged.connect(updateAlert)

#self.UI.diag.imv.sigTimeChanged.connect(self.UI.updateOpenCVEggROICurrEmbryo)

self.outputUI.diag.table.itemSelectionChanged.connect(self.supplyoutputUINewEmbryoData)

#self.UI.approveROI_btn.clicked.connect(self.saveUpdatedROI)

#test.UI.roi.sigRegionChangeFinished.connect(roiChanged)

app.exec_()

# Provide results data

def dataforViewOutput(self,n=0):

# Make embyo label current (for saving)

self.embryo = self.embryoLabels[int(n)]

# Load results

self.loadResults()

self.outputUI.results = self.results

self.getEmbryoFolders(self.parentPath, self.embryo)

# Send timeSeriesEmbryoBB data ...

self.outputUI.embryoBBRange = self.timeSeriesEmbryoBB()

def supplyoutputUINewEmbryoData(self):

# Debug

# print 'supplyUINewEmbryoData', self.UI.diag.table.currentRow()

currRow = self.outputUI.diag.table.currentRow()

self.dataforViewOutput(currRow)

self.outputUI.updateUI()

#==============================================================================

# Get Embryo bounding box max and min locations across entire data panel..

#==============================================================================

def timeSeriesEmbryoBB(self):

maxX = np.zeros(len(self.results))

maxY = np.zeros(len(self.results))

minX = np.zeros(len(self.results))

minY = np.zeros(len(self.results))

for f in range(len(self.results)):

self.currentFolder = self.embryoFolders[f]

self.shortenedPath = self.shortenedPaths[f]

#self.getShortenedPath()

out = self.getEmbryoBB()

maxX[f], maxY[f], minX[f], minY[f] = out['maxX'],out['maxY'],out['minX'],out['minY']

maxX = max(maxX)

maxY = max(maxY)

minX = min(minX)

minY = min(minY)

return {'minX':minX, 'minY':minY, 'maxX':maxX, 'maxY':maxY}

# Get Egg bounding box max and min locations across entire data panel..

def timeSeriesEggBB(self):

maxX = np.zeros(len(self.results))

maxY = np.zeros(len(self.results))

minX = np.zeros(len(self.results))

minY = np.zeros(len(self.results))

for f in range(len(self.results)):

self.currentFolder = self.embryoFolders[f]

self.shortenedPath = self.shortenedPaths[f]

out = self.getSeqEggBB()

maxX[f], maxY[f], minX[f], minY[f] = out['eggMaxX'],out['eggMaxY'],out['eggMinX'],out['eggMinY']

maxX = max(maxX)

maxY = max(maxY)

minX = min(minX)

minY = min(minY)

return {'minX':minX, 'minY':minY, 'maxX':maxX, 'maxY':maxY}

**imageAnalysis.py**

# Import dependencies

import cv2

import numpy as np

from skimage.segmentation import clear_border

import time

import skimage

import math

import pathos

class imageAnalysis(object):

#==============================================================================

# Identify the egg

#==============================================================================

def locateEgg(self,im):

#self.eggBBox = []

self.eggRotBBox = []

self.boxPoints = []

origIm = im.copy()

#kernel = np.ones((4,4),np.uint8)

# Apply otsu thresholding to ID egg

ret2,thresh = cv2.threshold(im,0,255,cv2.THRESH_BINARY+cv2.THRESH_OTSU)

# Open to remove all small/thin elements, including egg (usually leaving blobs and embryo.)

s1 = cv2.getStructuringElement(cv2.MORPH_ELLIPSE, (12,12))

thresh2 = cv2.morphologyEx(thresh, cv2.MORPH_OPEN, s1)

# Debug

# plt.imshow(thresh2)

# Now use inverted threshold of the opened image to remove embryo and other large items from original OTSU threshold.

origIm[thresh == thresh2] =0

# Apply adaptive threshold

origIm = cv2.adaptiveThreshold(origIm, 255, cv2.ADAPTIVE_THRESH_MEAN_C, cv2.THRESH_BINARY, 101,0)

# Use stats to remove non-egg candidates (rather than erode, which tends to remove too much egg)

num, labels, stats, centroids = cv2.connectedComponentsWithStats(origIm)

# 0 = n of blobs, 1 = labelled matrix, 2 = stats (leftMostX, topMostY, Width, Height, Area), 3 = centroids (X and Y

# Calculate distance from centre of image

disFrCen = np.sqrt(((centroids[:,0] - (im.shape[0]/2))**2) + ((centroids[:,1] - (im.shape[1]/2))**2))

# Get longest axis

lon = np.amax(stats[:,2:3], axis=1)

# Transpose stats array and add the extras

stats = stats.transpose()

stats = np.vstack([stats, centroids[:,0],centroids[:,1], disFrCen, lon, range(num)])

# Now filter the blobs

filtStats = stats[9,(stats[8,:]>20) & (stats[7,:]<(np.amax(im.shape[1:2])/0.8))& (stats[8,:]<(np.amax(origIm.shape[1:2])))]

if filtStats.shape[0] ==0:

#self.eggBBox=['nan','nan','nan','nan']

self.eggRotBBox = ['nan','nan','nan','nan','nan']

self.boxPoints = (('nan','nan'),('nan','nan'),('nan','nan'),('nan','nan'))

else:

#ind = int(ind)

mask = np.zeros_like(labels)

for m in range(len(filtStats)):

mask[labels == int(filtStats[m])]=1

# Debug

# plt.imshow(mask)

# Clear border

origIm = clear_border(mask, buffer_size=20, bgval=0)

# plt.imshow(testim)

# Find contours

contIm, contour,hier = cv2.findContours(origIm,cv2.RETR_CCOMP,cv2.CHAIN_APPROX_SIMPLE)

# If no contours found then do not continue and store nans.

if len(contour) ==0:

#self.eggBBox=['nan','nan','nan','nan']

self.eggRotBBox = ['nan','nan','nan','nan','nan']

self.boxPoints = (('nan','nan'),('nan','nan'),('nan','nan'),('nan','nan'))

else:

# Collate contours

self.eggCont = cv2.fitEllipse(np.concatenate(contour))

# Draw ellipse on blank image

eggEllipse = cv2.ellipse(np.zeros_like(im),self.eggCont,(1,1,1),-1)

# Debug

# np.zeros(im.shape,np.bool)

# plt.imshow(cv2.ellipse(origIm,eggCont,(255,0,0),2))

# imcopy[j] = cv2.bitwise_and(thresh,imcopy[j])

# pg.image(imcopy)

# plt.imshow(cv2.drawContours(imcopy[j],np.concatenate(contour),-1,(255,0,0),20))

# Use ellipse to get bounding box (both non rotated for im cropping and rotated for egg gui tweaking).

_,tmp,_ = cv2.findContours(eggEllipse,cv2.RETR_CCOMP,cv2.CHAIN_APPROX_SIMPLE)

# If nothing returned, then eggID unsuccesful so return NAs

if len(tmp)!=1:

#self.eggBBox=['nan','nan','nan','nan']

self.eggRotBBox = ['nan','nan','nan','nan','nan']

self.boxPoints = (('nan','nan'),('nan','nan'),('nan','nan'),('nan','nan'))

else:

# NonRotated BBox dimensions

#eggBBx,eggBBy,eggBBw,eggBBh = cv2.boundingRect(tmp[0])

#self.eggBBox = [eggBBx,eggBBy,eggBBw,eggBBh]

# Rotated BBox dimensions

(eggBBRotCentX, eggBBRotCentY), (eggBBRotWid, eggBBRotHei), eggBBRotAng = cv2.minAreaRect(tmp[0])

self.eggRotBBox = [eggBBRotCentX, eggBBRotCentY,eggBBRotWid, eggBBRotHei,eggBBRotAng]

# Get corners for rotated BB

self.boxPoints = cv2.boxPoints(cv2.minAreaRect(tmp[0]))

#return {'successful':successful, 'eggCont':eggCont, 'eggBBox':eggBBox, 'eggRotBBox':eggRotBBox, 'eggCentXY':eggCentXY, 'boxPoints':boxPoints}

#===============================================================================

# Segment and measure embryo spatial characteristics

#==============================================================================

def segmentEmbryo(self,n):

if self.species == 'rbalthica':

# Oli - Note - 7 kernel size upto 3/11. Increased to 9 to try and overcome lack of

# early stage segmentation.

blobs = self.auto_thresh(cv2.medianBlur(self.eggSeq[n],9),0.01)

cntMask = np.zeros((self.eggSeq[n].shape[0], self.eggSeq[n].shape[1]),dtype = np.uint8)

im2, conts, hierarchy = cv2.findContours(blobs,cv2.RETR_TREE,cv2.CHAIN_APPROX_SIMPLE)

for cnt in conts:

if cv2.arcLength(cnt, True) > 200:

cv2.drawContours(cntMask,[cnt],-1,(255,255,255),-1)

# Open to remove all small/thin elements, including egg (usually leaving blobs and embryo.)

s1 = cv2.getStructuringElement(cv2.MORPH_ELLIPSE, (12,12))

cntMask = cv2.morphologyEx(cntMask, cv2.MORPH_OPEN, s1)

# Get properties of thresholded blobs

# 0 = n of blobs, 1 = labelled matrix, 2 = stats (leftMostX, topMostY, Width, Height, Area), 3 = centroids (X and Y)

num, labels, stats, centroids = cv2.connectedComponentsWithStats(cntMask)

# Calculate extent - region area/bounding box area

extent = stats[:,4]/(stats[:,2].astype(float)*stats[:,3])

# Calculate aspect ratio (L/W)

asp = stats[:,2]/stats[:,3].astype(float)

for aspRat in range(len(asp)):

if asp[aspRat] < 1:

continue

else:

asp[aspRat] = 1/asp[aspRat]

# Format and combine extra descriptors

filtStats = stats.transpose()

filtStats = np.vstack([filtStats,extent,asp,range(len(stats))])

# Filter blobs

# Oli (16/11) changed min area from 5000 to 4000 to 3500 to 2000.

ind = filtStats[7,(filtStats[4,]>2000) & (filtStats[5,]>0.5) & (filtStats[3,]<labels.shape[0])]

if ind.shape[0] ==1:

ind = int(ind)

# Debug - visualise output

# self.seq[n] = cv2.bitwise_and(self.seq[n],self.seq[n],mask= np.array(labels == ind,dtype='uint8'))

im2, conts, hierarchy = cv2.findContours(np.array(labels == ind,dtype='uint8'),cv2.RETR_TREE,cv2.CHAIN_APPROX_SIMPLE)

for cnt in conts:

# cv2.arcLength(cnt, True) > 200:

if len(conts) ==1:

cv2.drawContours(cntMask,[cnt],-1,(255,255,255),-1)

embryoOutline = cnt

# Moments

M = cv2.moments(embryoOutline)

# Centroid

cx = int(M['m10']/M['m00'])

cy = int(M['m01']/M['m00'])

# Area

area = M['m00']

# Aspect ratio

x,y,w,h = cv2.boundingRect(embryoOutline)

aspect = float(w)/h

# Extent

rect_area = w*h

extent = float(area)/rect_area

# Solidity

hull = cv2.convexHull(embryoOutline)

hullArea = cv2.contourArea(hull)

solidity = float(area)/hullArea

else:

embryoOutline = np.NaN

cx = np.NaN

cy = np.NaN

area = np.NaN

x = np.NaN

y = np.NaN

w = np.NaN

h = np.NaN

solidity = np.NaN

hullArea = np.NaN

extent = np.NaN

aspect = np.NaN

else:

embryoOutline = np.NaN

cx = np.NaN

cy = np.NaN

area = np.NaN

x = np.NaN

y = np.NaN

w = np.NaN

h = np.NaN

solidity = np.NaN

hullArea = np.NaN

extent = np.NaN

aspect = np.NaN

#'Embryo':embryo,

elif self.species == 'ogammarellus':

# If not R.balthica, perform this segmentation approach...

# NOTE: This is a more crude approach - using the

# bounding box of the egg, rather than a complete embryo segmentation

# which for species such as O. gammarellus that completely fill the egg

# capsule is not biologically relevant.)

s1 = cv2.getStructuringElement(cv2.MORPH_ELLIPSE, (19,19))

mask = cv2.morphologyEx(self.auto_thresh(self.seq[n]), cv2.MORPH_CLOSE, s1)

s1 = cv2.getStructuringElement(cv2.MORPH_ELLIPSE, (3,3))

mask = cv2.morphologyEx(mask, cv2.MORPH_OPEN, s1)

cntMask = np.zeros((self.seq[n].shape[0], self.seq[n].shape[1]),dtype = np.uint8)

im2, conts, hierarchy = cv2.findContours(mask,cv2.RETR_TREE,cv2.CHAIN_APPROX_SIMPLE)

for cnt in conts:

if cv2.arcLength(cnt, True) > 200:

cv2.drawContours(cntMask,[cnt],-1,(255,255,255),-1)

# Get properties of thresholded blobs

# 0 = n of blobs, 1 = labelled matrix, 2 = stats (leftMostX, topMostY, Width, Height, Area), 3 = centroids (X and Y)

num, labels, stats, centroids = cv2.connectedComponentsWithStats(cntMask)

#plt.imshow(labels)

# Filter to big blob (gammarid)

# Filter blobs

#filtStats = stats[np.invert(stats[:,2] == self.seq[n].shape[1]),:]

if (stats.shape[0] is 2):

# Get index for filtering blob. Discounting the background i.e. size of im.

# ind = np.where(np.invert(stats[:,2] == self.seq[n].shape[1]))[0][0]

# maskArr = np.array(labels==ind)

# Uncomment to apply mask to original image

#im = cv2.bitwise_and(im,im, mask = maskArr.astype(np.uint8))

embryoOutline = cnt

# Get stats

# Moments

M = cv2.moments(embryoOutline)

# Centroid

cx = int(M['m10']/M['m00'])

cy = int(M['m01']/M['m00'])

# Area

area = M['m00']

# Aspect ratio

x,y,w,h = cv2.boundingRect(embryoOutline)

aspect = float(w)/h

# Extent - region area/bounding box area

extent = area/(w*h)

# Solidity

hull = cv2.convexHull(cnt)

hullArea = cv2.contourArea(hull)

solidity = float(area)/hullArea

else:

embryoOutline = np.NaN

cx = np.NaN

cy = np.NaN

area = np.NaN

x = np.NaN

y = np.NaN

w = np.NaN

h = np.NaN

solidity = np.NaN

hullArea = np.NaN

extent = np.NaN

aspect = np.NaN

return {'n':n,'outline':embryoOutline,'centX': cx,'centY': cy, 'ar': area,'aspect': aspect, 'extent': extent,'hullArea': hullArea,'solidity': solidity,'bboxMinCol': x,'bboxMinRow': y,'bboxWidth': w,'bboxHeight': h}

#==============================================================================#==============================================================================

# Run embryo segmentation

#==============================================================================

def runEmbryoSegmentation(self):

# Loop over the folders for an embryo and apply segmentation.

for f in range(len(self.embryoFolders)):

# Debug

#print f

totts = time.time()

self.currentFolder = self.embryoFolders[f]

self.shortenedPath = self.shortenedPaths[f]

print 'Starting: ', self.embryoFolders[f]

# If no egg dims in first row, do not continue with attempting segmentation.

if (self.results.loc[self.shortenedPath]['eggRotBBox'][0] == ['nan','nan','nan','nan','nan'])|(math.isnan(float(self.results.loc[self.shortenedPath]['eggRotBBox'][0][0])))| (self.results.loc[self.shortenedPath]['eggRotBBox'][0] == 'nan')| (self.results.loc[self.shortenedPath]['eggRotBBox'][0] == np.NaN):

self.results.loc[self.shortenedPath]['embryoOutline'] = np.NaN

self.results.loc[self.shortenedPath]['centroidX'] = np.NaN

self.results.loc[self.shortenedPath]['centroidY'] = np.NaN

self.results.loc[self.shortenedPath]['area'] = np.NaN

self.results.loc[self.shortenedPath]['aspect'] = np.NaN

self.results.loc[self.shortenedPath]['extent'] = np.NaN

self.results.loc[self.shortenedPath]['hullArea'] = np.NaN

self.results.loc[self.shortenedPath]['solidity'] = np.NaN

self.results.loc[self.shortenedPath]['bboxMincol'] = np.NaN

self.results.loc[self.shortenedPath]['bboxMinrow'] = np.NaN

self.results.loc[self.shortenedPath]['bboxWidth'] = np.NaN

self.results.loc[self.shortenedPath]['bboxHeight'] = np.NaN

self.results.loc[self.shortenedPath]['blockWise'] = np.NaN

print 'No egg for', self.results.loc[self.shortenedPath]['currentFolder'][0]

else:

self.seqImport(f)

# Mask seq using egg masks

#self.getSeqEggBB()

self.applyEggMaskAndCrop()

# Create a threading pool

innerPool = pathos.multiprocessing.ThreadingPool()

# Run segmentation

res = innerPool.map(self.segmentEmbryo, range(self.eggSeq.shape[0]))

# Save to dataframe

for g in range(len(res)):

self.results.loc[self.shortenedPath]['embryoOutline'][g] = res[g]['outline']

self.results.loc[self.shortenedPath]['centroidX'][g] = res[g]['centX']

self.results.loc[self.shortenedPath]['centroidY'][g] = res[g]['centY']

self.results.loc[self.shortenedPath]['area'][g] = res[g]['ar']

self.results.loc[self.shortenedPath]['aspect'][g] = res[g]['aspect']

self.results.loc[self.shortenedPath]['extent'][g] = res[g]['extent']

self.results.loc[self.shortenedPath]['hullArea'][g] = res[g]['hullArea']

self.results.loc[self.shortenedPath]['solidity'][g] = res[g]['solidity']

self.results.loc[self.shortenedPath]['bboxMincol'][g] = res[g]['bboxMinCol']

self.results.loc[self.shortenedPath]['bboxMinrow'][g] = res[g]['bboxMinRow']

self.results.loc[self.shortenedPath]['bboxWidth'][g] = res[g]['bboxWidth']

self.results.loc[self.shortenedPath]['bboxHeight'][g] = res[g]['bboxHeight']

# And create a cropped seq

#self.emSeq = self.seq[:,self.minY:self.maxY, self.minX:self.maxX]

res = innerPool.map(self.runNestedMeanWindowCalc, range(self.seq.shape[0]))

for g in range(len(res)):

self.results.loc[self.shortenedPath]['blockWise'][g] = res[g]

print self.embryo, ':', self.shortenedPath, 'analysed in {} s'.format(time.time()-totts)

# Save

self.results.to_pickle(self.resultsDir + "/" + self.embryo + '.pandas')

#==============================================================================

# Segment/analyse particular time points and add these to the results.

#==============================================================================

def segmentSpecificTimePoints(self):

print 'Loading results for: ' + self.embryo

# Load embryo

self.loadResults()

self.getEmbryoFolders(self.parentPath, self.embryo)

# Identify missing data

indicies = np.isnan(np.nanmean(self.results.ix[:,:,'area'].values.astype(np.float),axis=0))

inds = np.nonzero(indicies)[0]

# Loop over missing time points and perform analysis.

print str(len(inds)) + ' time points with missing data identified'

for t in range(len(inds)):

f = inds[t]

totts = time.time()

self.currentFolder = self.embryoFolders[f]

self.shortenedPath = self.shortenedPaths[f]

#self.getShortenedPath()

#self.shortenedPath = os.path.relpath(self.currentFolder, self.parentPath)

print 'Starting: ', self.embryoFolders[f]

# If no egg dims in first row, do not continue with attempting segmentation.

if (self.results.loc[self.shortenedPath]['eggRotBBox'][0] == ['nan','nan','nan','nan','nan'])|(math.isnan(float(self.results.loc[self.shortenedPath]['eggRotBBox'][0][0])))| (self.results.loc[self.shortenedPath]['eggRotBBox'][0] == 'nan')| (self.results.loc[self.shortenedPath]['eggRotBBox'][0] == np.NaN):

self.results.loc[self.shortenedPath]['embryoOutline'] = np.NaN

self.results.loc[self.shortenedPath]['centroidX'] = np.NaN

self.results.loc[self.shortenedPath]['centroidY'] = np.NaN

self.results.loc[self.shortenedPath]['area'] = np.NaN

self.results.loc[self.shortenedPath]['aspect'] = np.NaN

self.results.loc[self.shortenedPath]['extent'] = np.NaN

self.results.loc[self.shortenedPath]['hullArea'] = np.NaN

self.results.loc[self.shortenedPath]['solidity'] = np.NaN

self.results.loc[self.shortenedPath]['bboxMincol'] = np.NaN

self.results.loc[self.shortenedPath]['bboxMinrow'] = np.NaN

self.results.loc[self.shortenedPath]['bboxWidth'] = np.NaN

self.results.loc[self.shortenedPath]['bboxHeight'] = np.NaN

self.results.loc[self.shortenedPath]['blockWise'] = np.NaN

print 'No egg for', self.results.loc[self.shortenedPath]['currentFolder'][0]

else:

self.seqImport(f)

# Mask seq using egg masks

#self.getSeqEggBB()

self.applyEggMaskAndCrop()

# Create a threading pool

innerPool = pathos.multiprocessing.ThreadingPool()

# Run segmentation

res = innerPool.map(self.segmentEmbryo, range(self.eggSeq.shape[0]))

# Save to dataframe

for g in range(len(res)):

self.results.loc[self.shortenedPath]['embryoOutline'][g] = res[g]['outline']

self.results.loc[self.shortenedPath]['centroidX'][g] = res[g]['centX']

self.results.loc[self.shortenedPath]['centroidY'][g] = res[g]['centY']

self.results.loc[self.shortenedPath]['area'][g] = res[g]['ar']

self.results.loc[self.shortenedPath]['aspect'][g] = res[g]['aspect']

self.results.loc[self.shortenedPath]['extent'][g] = res[g]['extent']

self.results.loc[self.shortenedPath]['hullArea'][g] = res[g]['hullArea']

self.results.loc[self.shortenedPath]['solidity'][g] = res[g]['solidity']

self.results.loc[self.shortenedPath]['bboxMincol'][g] = res[g]['bboxMinCol']

self.results.loc[self.shortenedPath]['bboxMinrow'][g] = res[g]['bboxMinRow']

self.results.loc[self.shortenedPath]['bboxWidth'][g] = res[g]['bboxWidth']

self.results.loc[self.shortenedPath]['bboxHeight'][g] = res[g]['bboxHeight']

# And create a cropped seq

#self.emSeq = self.seq[:,self.minY:self.maxY, self.minX:self.maxX]

res = innerPool.map(self.runNestedMeanWindowCalc, range(self.seq.shape[0]))

for g in range(len(res)):

self.results.loc[self.shortenedPath]['blockWise'][g] = res[g]

print self.embryo, ':', self.shortenedPath, 'analysed in {} s'.format(time.time()-totts)

tmp = len(np.nonzero(np.isnan(np.nanmean(self.results.ix[:,:,'area'].values.astype(np.float),axis=0)))[0])

print str(tmp) + ' time points with missing data identified AFTER re-analysis.'

# Save

self.results.to_pickle(self.resultsDir + "/" + self.embryo + '.pandas')

print self.embryo + ' analysis complete'

#==============================================================================

# Parallel segmentation

#==============================================================================

def runParEmbryoSegmentation(self):

if self.species == 'rbalthica':

# Loop over the folders for an embryo and apply segmentation.

totts = time.time()

for f in range(len(self.embryoFolders)):

# Debug

print 'Starting: ', self.embryoFolders[f]

ts = time.time()

self.currentFolder = self.embryoFolders[f]

self.shortenedPath = self.shortenedPaths[f]

#self.getShortenedPath()

#self.shortenedPath = os.path.relpath(self.currentFolder, self.parentPath)

# If no egg dims in first row, do not continue with attempting segmentation.

if (self.results.loc[self.shortenedPath]['eggRotBBox'][0] == ['nan','nan','nan','nan','nan'])|(math.isnan(float(self.results.loc[self.shortenedPath]['eggRotBBox'][0][0])))|(self.results.loc[self.shortenedPath]['eggRotBBox'][0] == 'nan')| (self.results.loc[self.shortenedPath]['eggRotBBox'][0] == np.NaN):

self.results.loc[self.shortenedPath]['embryoOutline'] = np.NaN

self.results.loc[self.shortenedPath]['centroidX'] = np.NaN

self.results.loc[self.shortenedPath]['centroidY'] = np.NaN

self.results.loc[self.shortenedPath]['area'] = np.NaN

self.results.loc[self.shortenedPath]['aspect'] = np.NaN

self.results.loc[self.shortenedPath]['extent'] = np.NaN

self.results.loc[self.shortenedPath]['hullArea'] = np.NaN

self.results.loc[self.shortenedPath]['solidity'] = np.NaN

self.results.loc[self.shortenedPath]['bboxMincol'] = np.NaN

self.results.loc[self.shortenedPath]['bboxMinrow'] = np.NaN

self.results.loc[self.shortenedPath]['bboxWidth'] = np.NaN

self.results.loc[self.shortenedPath]['bboxHeight'] = np.NaN

self.results.loc[self.shortenedPath]['blockWise'] = np.NaN

print 'No egg for', self.results.loc[self.shortenedPath]['currentFolder'][0]

else:

self.seqImport(f)

self.applyEggMaskAndCrop()

print 'debug starting segmentation'

# Create a threading pool

innerPool = pathos.multiprocessing.ThreadingPool()

# Run segmentation

res = innerPool.map(self.segmentEmbryo, range(self.eggSeq.shape[0]))

# Save to dataframe

for g in range(len(res)):

self.results.loc[self.shortenedPath]['embryoOutline'][g] = res[g]['outline']

self.results.loc[self.shortenedPath]['centroidX'][g] = res[g]['centX']

self.results.loc[self.shortenedPath]['centroidY'][g] = res[g]['centY']

self.results.loc[self.shortenedPath]['area'][g] = res[g]['ar']

self.results.loc[self.shortenedPath]['aspect'][g] = res[g]['aspect']

self.results.loc[self.shortenedPath]['extent'][g] = res[g]['extent']

self.results.loc[self.shortenedPath]['hullArea'][g] = res[g]['hullArea']

self.results.loc[self.shortenedPath]['solidity'][g] = res[g]['solidity']

self.results.loc[self.shortenedPath]['bboxMincol'][g] = res[g]['bboxMinCol']

self.results.loc[self.shortenedPath]['bboxMinrow'][g] = res[g]['bboxMinRow']

self.results.loc[self.shortenedPath]['bboxWidth'][g] = res[g]['bboxWidth']

self.results.loc[self.shortenedPath]['bboxHeight'][g] = res[g]['bboxHeight']

# And create a cropped seq

#self.emSeq = self.seq[:,self.minY:self.maxY, self.minX:self.maxX]

res = innerPool.map(self.runNestedMeanWindowCalc, range(self.seq.shape[0]))

# Put data into Pandas DP

for g in range(len(res)):

self.results.loc[self.shortenedPath]['blockWise'][g] = res[g]

print self.embryo, ':', self.shortenedPath, 'analysed in {} s'.format(time.time()-ts)

print self.embryo, ':', ' results currently being saved.'

# Save

self.results.to_pickle(self.resultsDir + "/" + self.embryo + '.pandas')

print self.embryo,':',' results saved!'

print 'Analysis complete for', self.embryo, ' in {} s'.format(time.time()-totts)

# If Orchestia ..

elif self.species == 'ogammarellus':

# Loop over the folders for an embryo and apply segmentation.

for f in range(len(self.embryoFolders)):

print 'Starting: ', self.embryoFolders[f]

ts = time.time()

self.currentFolder = self.embryoFolders[f]

self.shortenedPath = self.shortenedPaths[f]

# Debug

print 'Analysing: ', self.embryoFolders[f]

ts = time.time()

self.currentFolder = self.embryoFolders[f]

self.shortenedPath = self.shortenedPaths[f]

self.seqImport(f)

#self.applyEggMaskAndCrop()

# Create a threading pool

innerPool = pathos.multiprocessing.ThreadingPool()

# Run segmentation

res = innerPool.map(self.segmentEmbryo, range(self.seq.shape[0]))

# Save to dataframe

for g in range(len(res)):

self.results.loc[self.shortenedPath]['embryoOutline'][g] = res[g]['outline']

self.results.loc[self.shortenedPath]['centroidX'][g] = res[g]['centX']

self.results.loc[self.shortenedPath]['centroidY'][g] = res[g]['centY']

self.results.loc[self.shortenedPath]['area'][g] = res[g]['ar']

self.results.loc[self.shortenedPath]['aspect'][g] = res[g]['aspect']

self.results.loc[self.shortenedPath]['extent'][g] = res[g]['extent']

self.results.loc[self.shortenedPath]['hullArea'][g] = res[g]['hullArea']

self.results.loc[self.shortenedPath]['solidity'][g] = res[g]['solidity']

self.results.loc[self.shortenedPath]['bboxMincol'][g] = res[g]['bboxMinCol']

self.results.loc[self.shortenedPath]['bboxMinrow'][g] = res[g]['bboxMinRow']

self.results.loc[self.shortenedPath]['bboxWidth'][g] = res[g]['bboxWidth']

self.results.loc[self.shortenedPath]['bboxHeight'][g] = res[g]['bboxHeight']

# And create a cropped seq

#self.emSeq = self.seq[:,self.minY:self.maxY, self.minX:self.maxX]

res = innerPool.map(self.runNestedMeanWindowCalc, range(self.seq.shape[0]))

# Put data into Pandas DP

for g in range(len(res)):

self.results.loc[self.shortenedPath]['blockWise'][g] = res[g]

print self.embryo, ':', self.shortenedPath, 'analysed in {} s'.format(time.time()-ts)

print self.embryo, ':', ' results currently being saved.'

# Save

self.results.to_pickle(self.resultsDir + "/" + self.embryo + '.pandas')

print self.embryo,':',' results saved!'

print 'Analysis complete for', self.embryo, ' in {} s'.format(time.time()-totts)

#==============================================================================

# def parSeg(self, n):

# # Segment embryo

# self.segmentEmbryo(n)

# return {'n':n, 'Embryo':self.embryo,'outline':self.embryoOutline,'centX': self.cx,'centY': self.cy, 'ar': self.area,'aspect': self.aspect, 'extent': self.extent,'hullArea': self.hullArea,'solidity': self.solidity,'bboxMinCol': self.x,'bboxMinRow': self.y,'bboxWidth': self.w,'bboxHeight': self.h}

#

#==============================================================================

# Save

# self.results.to_pickle(self.resultsDir + "/" + self.embryo + '.pandas')

#==============================================================================

# Mask outside of egg from seq stack

#==============================================================================

def applyEggMask(self):

# Take eggRotBBox from results, format appropriately and apply mask to self.seq

for g in range(self.seq.shape[0]):

# Apply egg mask.

formattedRotBBox = ((self.results[self.shortenedPath]['eggRotBBox'][g][0],self.results[self.shortenedPath]['eggRotBBox'][g][1]),

(self.results[self.shortenedPath]['eggRotBBox'][g][2],self.results[self.shortenedPath]['eggRotBBox'][g][3]),

(self.results[self.shortenedPath]['eggRotBBox'][g][4]))

out = cv2.ellipse(np.zeros_like(self.seq[g]),formattedRotBBox,(255,255,255),-1)

self.seq[g] = cv2.bitwise_and(self.seq[g], out)

#==============================================================================

# Mask outside of egg from seq stack

#==============================================================================

def applyEggMaskAndCrop(self):

# Reduce seq to egg BB and apply egg mask.

out = self.getSeqEggBB()

eggMinX, eggMinY, eggMaxX, eggMaxY = out['eggMinX'], out['eggMinY'], out['eggMaxX'], out['eggMaxY']

formattedRotBBox = ((self.results[self.shortenedPath]['eggRotBBox'][0][0]-eggMinX,self.results[self.shortenedPath]['eggRotBBox'][0][1]-eggMinY),

(self.results[self.shortenedPath]['eggRotBBox'][0][2],self.results[self.shortenedPath]['eggRotBBox'][0][3]),

(self.results[self.shortenedPath]['eggRotBBox'][0][4]))

self.eggSeq = self.seq[:,eggMinY:eggMaxY,eggMinX:eggMaxX]

for g in range(self.eggSeq.shape[0]):

out = cv2.ellipse(np.zeros_like(self.eggSeq[g]),formattedRotBBox,(255,255,255),-1)

self.eggSeq[g] = cv2.bitwise_and(self.eggSeq[g], out)

#==============================================================================

# Get egg ROI

#==============================================================================

def getSeqEggBB(self):

#self.results.ix[self.shortenedPath,:,'eggBoxPoints']

# Currently assumes egg ROI fixed during sequence..

eggMinX = int(np.array(self.results.ix[self.shortenedPath,:,'eggBoxPoints'][0])[:,0].min())

eggMaxX = int(np.array(self.results.ix[self.shortenedPath,:,'eggBoxPoints'][0])[:,0].max())

eggMinY = int(np.array(self.results.ix[self.shortenedPath,:,'eggBoxPoints'][0])[:,1].min())

eggMaxY = int(np.array(self.results.ix[self.shortenedPath,:,'eggBoxPoints'][0])[:,1].max())

# Check if min or max are outside im region, in which restrict..

if (eggMinX < 0) | (eggMinY < 0) | (eggMinX < 0) | (eggMinX < 0):

if (eggMinX < 0):

eggMinX = 0

if (eggMinY < 0):

eggMinY = 0

if (eggMaxX > self.seq.shape[2]):

eggMaxX = self.seq.shape[2]

if (eggMaxY > self.seq.shape[1]):

eggMaxY = self.seq.shape[1]

return {'eggMinX':eggMinX, 'eggMinY':eggMinY, 'eggMaxX':eggMaxX, 'eggMaxY':eggMaxY}

#cv2.boundingRect()

#==============================================================================

# Crop seq to embryo BB

#==============================================================================

def getEmbryoBB(self):

# If Radix balthica...

if self.species == 'rbalthica':

# Check for python nan

tmp=[]

for f in self.results.ix[self.shortenedPath,:,'bboxWidth'].values:

tmp.append(math.isnan(f))

if np.sum(tmp)>0|np.sum(self.results.ix[self.shortenedPath,:,'bboxWidth'] =='nan')|np.sum(self.results.ix[self.shortenedPath,:,'bboxWidth'] ==np.NaN) >0|np.sum(self.results.ix[self.shortenedPath,:,'eggBoxPoints']==np.NaN) >0:

maxX = np.NaN

maxY = np.NaN

minX = np.NaN

minY = np.NaN

else:

# Get egg BB to add

out = self.getSeqEggBB()

eggMinX, eggMinY, eggMaxX, eggMaxY = out['eggMinX'], out['eggMinY'], out['eggMaxX'], out['eggMaxY']

maxX = eggMinX + np.max(self.results.ix[self.shortenedPath,:,'bboxWidth'] + self.results.ix[self.shortenedPath,:,'bboxMincol'])

maxY = eggMinY + np.max(self.results.ix[self.shortenedPath,:,'bboxHeight'] + self.results.ix[self.shortenedPath,:,'bboxMinrow'])

minX = eggMinX + self.results.ix[self.shortenedPath,:,'bboxMincol'].min()

minY = eggMinY + self.results.ix[self.shortenedPath,:,'bboxMinrow'].min()

# If Orchestia gammarellus..

elif self.species == 'ogammarellus':

# Check for python nan

maxX = np.max(self.results.ix[self.shortenedPath,:,'bboxWidth'] + self.results.ix[self.shortenedPath,:,'bboxMincol'])

maxY = np.max(self.results.ix[self.shortenedPath,:,'bboxHeight'] + self.results.ix[self.shortenedPath,:,'bboxMinrow'])

minX = self.results.ix[self.shortenedPath,:,'bboxMincol'].min()

minY = self.results.ix[self.shortenedPath,:,'bboxMinrow'].min()

return {'minX':minX, 'minY':minY, 'maxX':maxX, 'maxY':maxY}

#==============================================================================

# Get mean window calculation at multiple resolutions.. nested.

#==============================================================================

def runNestedMeanWindowCalc(self,f):

out = self.getEmbryoBB()

minX, minY, maxX, maxY = out['minX'], out['minY'], out['maxX'], out['maxY']

if (minX != np.NaN) and (not math.isnan(minX)):

#im = self.seq[f,self.minY:self.maxY, self.minX:self.maxX]

# Get shape dims

imy = maxY - minY

imx = maxX - minX

# To be able to divide by 2,4,8,16 the embryoBB may need to be expanded slightly.

# Use modulo 16 to find out to what value the range must be expanded to make this possible.

expandY = (imy%16)

expandX = (imx%16)

skipY = False

if expandY !=0:

# Test whether subtraction is required and if so modify expandY

if ((imy-imy%16)%16)==0:

expandY = 16 - (imy%16)

# If either minX or minY ==1 or max im size

if ((minY-(expandY/2)) <= 0) | ((maxY+(expandY/2))>self.seq.shape[1]):

if ((minY-(expandY/2)) <= 0):

maxY = (maxY + expandY)

imy = maxY - minY

skipY = True

elif ((maxY+(expandY/2))>self.seq.shape[1]):

minY = (minY - expandY)

imy = maxY - minY

skipY = True

# If expansion/contraction does not reach img limits...

if not skipY:

if (not((minY-(expandY/2)) <= 0)) | (not((maxY+(expandY/2))>self.seq.shape[1])):

# Even

if expandY%2 ==0:

minY = int(minY - (expandY/2))

maxY = int(maxY + (expandY/2))

imy = maxY - minY

# Odd

else:

minY = int(minY - (math.ceil(expandY/2.)))

maxY = int(maxY + (math.floor(expandY/2.)))

imy = maxY - minY

skipX = False

if expandX !=0:

# Test whether subtraction is required and if so modify expandY

if ((imx-imx%16)%16)==0:

expandX = 16 - (imx%16)

# If either minX or minY ==1 or max im size

if ((minX-(expandX/2)) <= 0) | ((maxX+(expandX/2))>self.seq.shape[2]):

if ((minX-(expandX/2)) <= 0):

maxX = (maxX + expandX)

imx = maxX - minX

skipX = True

elif ((maxX+(expandX/2))>self.seq.shape[2]):

minX = (minX - expandX)

imx = maxX - minX

skipX = True

# If expansion/contraction does not reach img limits...

if not skipX:

if (not((minX-(expandX/2)) <= 0)) | (not((maxX+(expandX/2))>self.seq.shape[2])):

# Even

if expandX%2 ==0:

minX = int(minX - (expandX/2))

maxX = int(maxX + (expandX/2))

imx = maxX - minX

# Odd

else:

minX = int(minX - (math.ceil(expandX/2.)))

maxX = int(maxX + (math.floor(expandX/2.)))

imx = maxX - minX

# Debug

imx = imx

imy = imy

# Extract frame relevant to f

im = np.ascontiguousarray(self.seq[f,minY:maxY, minX:maxX])

minWinSize =60

# 60 Pixel window calculations

rangeY = maxY - minY

rangeX = maxX - minX

winNoY = rangeY/minWinSize

winNoX = rangeX/minWinSize

# List to collect results

nestMeanWindOut = []

# First get overall embryoBB mean

nestMeanWindOut.append(im.mean())

# Now get 2 x 2 means

out = skimage.util.view_as_blocks(im,block_shape = (imy/2,imx/2))

nestMeanWindOut.append(((out[0,0].mean(),out[0,1].mean()),(out[1,0].mean(),out[1,1].mean())))

# Now get 4 x 4 means

out = skimage.util.view_as_blocks(im,block_shape = (imy/4,imx/4))

nestMeanWindOut.append(((out[0,0].mean(),out[0,1].mean(), out[0,2].mean(),out[0,3].mean()),

(out[1,0].mean(),out[1,1].mean(),out[1,2].mean(),out[1,3].mean()),

(out[2,0].mean(),out[2,1].mean(),out[2,2].mean(),out[2,3].mean()),

(out[3,0].mean(),out[3,1].mean(),out[3,2].mean(),out[3,3].mean())))

# Now get 8 x 8 means

out = skimage.util.view_as_blocks(im,block_shape = (imy/8,imx/8))

nestMeanWindOut.append(((out[0,0].mean(),out[0,1].mean(), out[0,2].mean(),out[0,3].mean(),

out[0,4].mean(),out[0,5].mean(),out[0,6].mean(),out[0,7].mean()),

(out[1,0].mean(),out[1,1].mean(), out[1,2].mean(),out[1,3].mean(),

out[1,4].mean(),out[1,5].mean(),out[1,6].mean(),out[1,7].mean()),

(out[2,0].mean(),out[2,1].mean(), out[2,2].mean(),out[2,3].mean(),

out[2,4].mean(),out[2,5].mean(),out[2,6].mean(),out[2,7].mean()),

(out[3,0].mean(),out[3,1].mean(), out[3,2].mean(),out[3,3].mean(),

out[3,4].mean(),out[3,5].mean(),out[3,6].mean(),out[3,7].mean()),

(out[4,0].mean(),out[4,1].mean(), out[4,2].mean(),out[4,3].mean(),

out[4,4].mean(),out[4,5].mean(),out[4,6].mean(),out[4,7].mean()),

(out[5,0].mean(),out[5,1].mean(), out[5,2].mean(),out[5,3].mean(),

out[5,4].mean(),out[5,5].mean(),out[5,6].mean(),out[5,7].mean()),

(out[6,0].mean(),out[6,1].mean(), out[6,2].mean(),out[6,3].mean(),

out[6,4].mean(),out[6,5].mean(),out[6,6].mean(),out[6,7].mean()),

(out[7,0].mean(),out[7,1].mean(), out[7,2].mean(),out[7,3].mean(),

out[7,4].mean(),out[7,5].mean(),out[7,6].mean(),out[7,7].mean())))

# Now get 16 x 16 means

out = skimage.util.view_as_blocks(im,block_shape = (imy/16,imx/16))

nestMeanWindOut.append(((out[0,0].mean(),out[0,1].mean(), out[0,2].mean(),out[0,3].mean(),

out[0,4].mean(),out[0,5].mean(),out[0,6].mean(),out[0,7].mean(),

out[0,8].mean(),out[0,9].mean(), out[0,10].mean(),out[0,11].mean(),

out[0,12].mean(),out[0,13].mean(),out[0,14].mean(),out[0,15].mean()),

(out[1,0].mean(),out[1,1].mean(), out[1,2].mean(),out[1,3].mean(),

out[1,4].mean(),out[1,5].mean(),out[1,6].mean(),out[1,7].mean(),

out[1,8].mean(),out[1,9].mean(), out[1,10].mean(),out[1,11].mean(),

out[1,12].mean(),out[1,13].mean(),out[1,14].mean(),out[1,15].mean()),

(out[2,0].mean(),out[2,1].mean(), out[2,2].mean(),out[2,3].mean(),

out[2,4].mean(),out[2,5].mean(),out[2,6].mean(),out[2,7].mean(),

out[2,8].mean(),out[2,9].mean(), out[2,10].mean(),out[2,11].mean(),

out[2,12].mean(),out[2,13].mean(),out[2,14].mean(),out[2,15].mean()),

(out[3,0].mean(),out[3,1].mean(), out[3,2].mean(),out[3,3].mean(),

out[3,4].mean(),out[3,5].mean(),out[3,6].mean(),out[3,7].mean(),

out[3,8].mean(),out[3,9].mean(), out[3,10].mean(),out[3,11].mean(),

out[3,12].mean(),out[3,13].mean(),out[3,14].mean(),out[3,15].mean()),

(out[4,0].mean(),out[4,1].mean(), out[4,2].mean(),out[4,3].mean(),

out[4,4].mean(),out[4,5].mean(),out[4,6].mean(),out[4,7].mean(),

out[4,8].mean(),out[4,9].mean(), out[4,10].mean(),out[4,11].mean(),

out[4,12].mean(),out[4,13].mean(),out[4,14].mean(),out[4,15].mean()),

(out[5,0].mean(),out[5,1].mean(), out[5,2].mean(),out[5,3].mean(),

out[5,4].mean(),out[5,5].mean(),out[5,6].mean(),out[5,7].mean(),

out[5,8].mean(),out[5,9].mean(), out[5,10].mean(),out[5,11].mean(),

out[5,12].mean(),out[5,13].mean(),out[5,14].mean(),out[5,15].mean()),

(out[6,0].mean(),out[6,1].mean(), out[6,2].mean(),out[6,3].mean(),

out[6,4].mean(),out[6,5].mean(),out[6,6].mean(),out[6,7].mean(),

out[6,8].mean(),out[6,9].mean(), out[6,10].mean(),out[6,11].mean(),

out[6,12].mean(),out[6,13].mean(),out[6,14].mean(),out[6,15].mean()),

(out[7,0].mean(),out[7,1].mean(), out[7,2].mean(),out[7,3].mean(),

out[7,4].mean(),out[7,5].mean(),out[7,6].mean(),out[7,7].mean(),

out[7,8].mean(),out[7,9].mean(), out[7,10].mean(),out[7,11].mean(),

out[7,12].mean(),out[7,13].mean(),out[7,14].mean(),out[7,15].mean()),

(out[8,0].mean(),out[8,1].mean(), out[8,2].mean(),out[8,3].mean(),

out[8,4].mean(),out[8,5].mean(),out[8,6].mean(),out[8,7].mean(),

out[8,8].mean(),out[8,9].mean(), out[8,10].mean(),out[8,11].mean(),

out[8,12].mean(),out[8,13].mean(),out[8,14].mean(),out[8,15].mean()),

(out[9,0].mean(),out[9,1].mean(), out[9,2].mean(),out[9,3].mean(),

out[9,4].mean(),out[9,5].mean(),out[9,6].mean(),out[9,7].mean(),

out[9,8].mean(),out[9,9].mean(), out[9,10].mean(),out[9,11].mean(),

out[9,12].mean(),out[9,13].mean(),out[9,14].mean(),out[9,15].mean()),

(out[10,0].mean(),out[10,1].mean(), out[10,2].mean(),out[10,3].mean(),

out[10,4].mean(),out[10,5].mean(),out[10,6].mean(),out[10,7].mean(),

out[10,8].mean(),out[10,9].mean(), out[10,10].mean(),out[10,11].mean(),

out[10,12].mean(),out[10,13].mean(),out[10,14].mean(),out[10,15].mean()),

(out[11,0].mean(),out[11,1].mean(), out[11,2].mean(),out[11,3].mean(),

out[11,4].mean(),out[11,5].mean(),out[11,6].mean(),out[11,7].mean(),

out[11,8].mean(),out[11,9].mean(), out[11,10].mean(),out[11,11].mean(),

out[11,12].mean(),out[11,13].mean(),out[11,14].mean(),out[11,15].mean()),

(out[12,0].mean(),out[12,1].mean(), out[12,2].mean(),out[12,3].mean(),

out[12,4].mean(),out[12,5].mean(),out[12,6].mean(),out[12,7].mean(),

out[12,8].mean(),out[12,9].mean(), out[12,10].mean(),out[12,11].mean(),

out[12,12].mean(),out[12,13].mean(),out[12,14].mean(),out[12,15].mean()),

(out[13,0].mean(),out[13,1].mean(), out[13,2].mean(),out[13,3].mean(),

out[13,4].mean(),out[13,5].mean(),out[13,6].mean(),out[13,7].mean(),

out[13,8].mean(),out[13,9].mean(), out[13,10].mean(),out[13,11].mean(),

out[13,12].mean(),out[13,13].mean(),out[13,14].mean(),out[13,15].mean()),

(out[14,0].mean(),out[14,1].mean(), out[14,2].mean(),out[14,3].mean(),

out[14,4].mean(),out[14,5].mean(),out[14,6].mean(),out[14,7].mean(),

out[14,8].mean(),out[14,9].mean(), out[14,10].mean(),out[14,11].mean(),

out[14,12].mean(),out[14,13].mean(),out[14,14].mean(),out[14,15].mean()),

(out[15,0].mean(),out[15,1].mean(), out[15,2].mean(),out[15,3].mean(),

out[15,4].mean(),out[15,5].mean(),out[15,6].mean(),out[15,7].mean(),

out[15,8].mean(),out[15,9].mean(), out[15,10].mean(),out[15,11].mean(),

out[15,12].mean(),out[15,13].mean(),out[15,14].mean(),out[15,15].mean())))

else:

nestMeanWindOut = np.NaN

return nestMeanWindOut

#%%

#==============================================================================

# Image segmentation methods

#==============================================================================

#%%

# Adaptive form of thresholding.

@staticmethod

def auto_thresh(image,sigma = 0.1):

v = np.mean(image[image!=0])

lower = int(max(0, (1.0 - sigma) * v))

upper = int(min(255, (1.0 + sigma) * v))

ret2,thresh = cv2.threshold(image,lower, upper,cv2.THRESH_BINARY+cv2.THRESH_OTSU)

# ret2,thresh = cv2.threshold(blur,0,255,cv2.THRESH_BINARY+cv2.THRESH_OTSU)

# Debug

# plt.imshow(thresh)

return thresh

#%%

@staticmethod

def auto_canny(image, sigma=0.33):

# compute the median of the single channel pixel intensities

v = np.median(image[image!=0])

# apply automatic Canny edge detection using the computed median

lower = int(max(0, (1.0 - sigma) * v))

upper = int(min(255, (1.0 + sigma) * v))

edged = cv2.Canny(image, lower, upper)

# return the edged image

return edged

#%%

@staticmethod

def auto_varSeg(image, sigma=0.33):

# compute the median of the single channel pixel intensities

v = np.mean(image[image!=0])

# apply automatic Canny edge detection using the computed median

lower = int(max(0, (1.0 - sigma) * v))

upper = int(min(255, (1.0 + sigma) * v))

edged = cv2.Canny(image, lower, upper)

# return the edged image

return edged

**dataAnalysis.py**

import cv2

import numpy as np

import matplotlib.pyplot as plt

import numpy.ma as ma

import pyqtgraph as pg

import pandas as pd

import glob

import os

import re

from skimage.segmentation import clear_border

from skimage.morphology import disk

from PyQt5.Qt import *

import sys

#from imageAnalysis import imageAnalysis

import eggUI

#import viewOutput

import time

import pathos

import json

#import tables

import scipy

import scipy.fftpack

import pylab

from scipy import pi

import scipy.signal as signal

import peakutils

import xarray as xr

import math

from rpy2.robjects.packages import importr

import rpy2.robjects as robjects

from matplotlib.backends.backend_pdf import PdfPages

import statsmodels.api as sm

import statsmodels.formula.api as smf

from sklearn import datasets, linear_model

from sklearn.metrics import mean_squared_error, r2_score

class dataAnalysis(object):

#==============================================================================

# Produce summary reports for each embryo and save to savePath.

# Note: savePath must exist i.e. create the folder beforehand..

#==============================================================================

def generateSummaryReports(self,savePath):

for e in range(len(self.embryoLabels)):

self.embryo = self.embryoLabels[e]

self.loadXRResults()

if self.results is not 'NoData':

data = self.results['TimeSpecificSummaryData'].to_pandas()

f, ax = plt.subplots(4, sharex=True)

ax[0].set_yscale('log')

# Size

for i in range(13,24):

ax[0].fill_between(np.arange(0,len(data.ix[:,i].values)),data.ix[:,i].values, alpha = 0.3)

ax[0].set_title('Enery at different frequencies')

ax[1].plot(data.ix[:,0].values)

ax[1].fill_between(np.arange(0,len(data.ix[:,1].values)),data.ix[:,1].values,data.ix[:,2].values, alpha = 0.3)

ax[1].set_title('Min:Max and mean area')

ax[2].plot(data.ix[:,3].values)

ax[2].fill_between(np.arange(0,len(data.ix[:,1].values)),data.ix[:,4].values,data.ix[:,5].values, alpha = 0.3)

ax[2].set_title('Min:Max and mean BB')

ax[3].plot(data.ix[:,7].values)

ax[3].set_yscale('log')

ax[3].fill_between(np.arange(0,len(data.ix[:,1].values)),data.ix[:,8].values,data.ix[:,9].values, alpha = 0.3)

ax[3].set_title('Min:Max and mean distance')

plt.tight_layout(pad=0.4, w_pad=0.5, h_pad=1.0)

pdf_pages = PdfPages(savePath + self.embryoLabels[e] + '_phenomeSummary.pdf')

pdf_pages.savefig(f)

pdf_pages.close()

plt.plot()

plt.close()

#==============================================================================

# Measure heart rate for all embryos

#==============================================================================

def measureHeartRateForAllEmbryos(self,savePath,filtMin=False,filtMax=False,histCount=False,segRegfiltXVal=False,segRegfiltYVal=False,minXVal=False,peakThresh=False):

if self.species == 'rbalthica':

# If user has not specified these 'tweakable paramters' in the HR ID

# and model fitting use these values

if filtMin == False:

filtMin = 1

if filtMax == False:

filtMax = 2

if histCount == False:

histCount = 6

if segRegfiltXVal == False:

segRegfiltXVal = 5

if segRegfiltYVal == False:

segRegfiltYVal = 6

if minXVal == False:

minXVal = 60

if peakThresh == False:

peakThresh = 0.4

for e in range(len(self.embryoLabels)):

self.embryo = self.embryoLabels[e]

self.loadXRResults()

self.measureHeartRate_radix(savePath,filtMin,filtMax,histCount,segRegfiltXVal,segRegfiltYVal,minXVal,peakThresh)

np.save(savePath + '/hrdata.npy', self.heartRate_data)

if self.species == 'ogammarellus':

# If user has not specified these 'tweakable paramters' in the HR ID

# and model fitting use these values

if filtMin == False:

filtMin = 0.5

if filtMax == False:

filtMax = 4

if histCount == False:

histCount = 6

if minXVal == False:

minXVal = False

if peakThresh == False:

peakThresh = 0.2

print str(savePath) + '_' + str(filtMin) + '_' +str(filtMax) + '_' + str(histCount) + '_' + str(peakThresh)

for e in range(len(self.embryoLabels)):

self.embryo = self.embryoLabels[e]

self.loadXRResults()

self.measureHeartRate_orchestia(savePath,filtMin,filtMax,histCount,peakThresh)

np.save(savePath + '/hrdata.npy', self.heartRate_data)

#==============================================================================

# Modify heart rate modelling for specific embryo

#==============================================================================

def measureHeartRateForSpecificEmbryos(self,embryo,savePath,filtMin=False,filtMax=False,histCount=False,segRegfiltXVal=False,segRegfiltYVal=False,minXVal=False,peakThresh=False):

if self.species == 'rbalthica':

# If user has not specified these 'tweakable paramters' in the HR ID

# and model fitting use these values

if filtMin == False:

filtMin = 1

if filtMax == False:

filtMax = 2

if histCount == False:

histCount = 6

if segRegfiltXVal == False:

segRegfiltXVal = 5

if segRegfiltYVal == False:

segRegfiltYVal = 6

if minXVal == False:

minXVal = 60

if peakThresh == False:

peakThresh = 0.4

if isinstance(embryo, str):

self.heartRate_data = np.load(savePath + '/hrdata.npy')

self.heartRate_data = self.heartRate_data[()]

self.embryo = embryo

self.loadXRResults()

self.measureHeartRate_radix(savePath,filtMin,filtMax,histCount,segRegfiltXVal,segRegfiltYVal,minXVal,peakThresh)

np.save(savePath + '/hrdata.npy', self.heartRate_data)

if isinstance(embryo, list):

self.heartRate_data = np.load(savePath + '/hrdata.npy')

self.heartRate_data = self.heartRate_data[()]

for e in range(len(embryo)):

self.embryo = embryo[e]

self.loadXRResults()

self.measureHeartRate_radix(savePath,filtMin,filtMax,histCount,segRegfiltXVal,segRegfiltYVal,minXVal,peakThresh)

np.save(savePath + '/hrdata.npy', self.heartRate_data)

elif self.species == 'ogammarellus':

if filtMin == None:

filtMin = 0.5

if filtMax == None:

filtMax = 4

if histCount == None:

histCount = 6

if minXVal == None:

minXVal = False

if peakThresh == None:

peakThresh = 0.2

if isinstance(embryo, str):

self.heartRate_data = np.load(savePath + '/hrdata.npy')

self.heartRate_data = self.heartRate_data[()]

self.embryo = embryo

self.loadXRResults()

self.measureHearRate_orchestia(self,savePath,filtMin,filtMax,histCount,peakThresh)

np.save(savePath + '/hrdata.npy', self.heartRate_data)

if isinstance(embryo, list):

self.heartRate_data = np.load(savePath + '/hrdata.npy')

self.heartRate_data = self.heartRate_data[()]

for e in range(len(embryo)):

self.embryo = embryo[e]

self.loadXRResults()

self.measureHearRate_orchestia(self,savePath,filtMin,filtMax,histCount,peakThresh)

np.save(savePath + '/hrdata.npy', self.heartRate_data)

#==============================================================================

# Load heart rate data

#==============================================================================

def loadHeartRateData(self,savePath):

self.heartRate_data = np.load(savePath + '/hrdata.npy')

self.heartRate_data = self.heartRate_data[()]

#==============================================================================

# Umbrella function for measuring heart rate - Radix balthica

#==============================================================================

def measureHeartRate_orchestia(self,savePath,filtMin,filtMax,histCount,peakThresh):

if not hasattr(self,'heartRate_data'):

self.heartRate_data = dict()

if self.results is 'NoData':

print 'No data for ' + str(self.embryo)

freqres = self.results['FreqOutput_8x8'].values

gs, fs = np.meshgrid(range(8),range(8))

gs = gs.flatten()

fs = fs.flatten()

HRs=[]

# Loop over time points

for t in range(self.results['FreqOutput_8x8'].values.shape[0]):

HRFreqs=[]

# Loop over blockwise signals

for b in range(len(gs)):

powerSpect = freqres[t,fs[b],gs[b],1,:]

if (powerSpect.max() == 0.0) or np.isnan(powerSpect[0]):

HRFreqs.append(np.NaN)

else:

sig = pd.rolling_mean(self.results['BlockWise_8x8'].loc[t,:,fs[b],gs[b]].values, window=3)

sig = pd.DataFrame(sig).interpolate(limit_direction = 'both').values.ravel()

# Interpolate to fill missing data

frameRate = sig.shape[0]/((self.results['SizePos'].to_pandas().ix[t,sig.shape[0]-1,'elapsedTime'] - self.results['SizePos'].to_pandas().ix[t,0,'elapsedTime'])/1000)

sampFreqs, powerSpect = signal.welch(sig,frameRate, scaling='spectrum')

baselineRemoved = np.log(powerSpect) - peakutils.baseline(np.log(powerSpect)- min(np.log(powerSpect)))

# Now ID peaks.anan

indexes = peakutils.indexes(baselineRemoved[0:200], peakThresh, min_dist=0)

outPeakFreqs = sampFreqs[indexes]

inds = np.where(((outPeakFreqs < filtMax) & (outPeakFreqs > filtMin)))

filtFreqs = outPeakFreqs[inds]

filtPower = baselineRemoved[inds]

# If more than one freq peak identified within the range 0.5-3 Hz

if inds[0].shape[0] > 1:

#print str(b)

# Identify freq with most power

maxInd = np.argmax(filtPower)

HRFreq = filtFreqs[maxInd]

HRFreqs.append(HRFreq)

elif inds[0].shape[0] == 1:

HRFreq = outPeakFreqs[inds[0][0]]

HRFreqs.append(HRFreq)

else:

HRFreqs.append(np.NaN)

else:

HRFreqs.append(np.NaN)

HRFreqs = np.array(HRFreqs)

# Remove NaNs

count, freqs = np.histogram(HRFreqs[np.invert(np.isnan(HRFreqs))],bins=50)

# Identify whether a sufficiently dominant frequency to reliably ID HR.

inds = np.where(count > histCount)

#plt.hist(HRFreqs[~np.isnan(HRFreqs)],20)

if len(inds[0]) is not 0:

# Get max ind

maxInds = inds[-1]

HR = np.mean(freqs[maxInds])

print HR

HRs.append(HR)

else:

HRs.append(np.NaN)

# Attempt to fit a linear model to data from 24 h post HR detection..

try:

# Use for fitting model

reducedHRs = HRs[23:]

# Fit a linear model to Orchestia heart function ontogeny

# Format data

pdData = np.array([np.arange(len(reducedHRs))[~np.isnan(reducedHRs)], np.array(reducedHRs)[~np.isnan(reducedHRs)]]).T

df = pd.DataFrame(pdData,columns=['Ind','HR'])

mod = smf.ols(formula='HR ~ Ind', data=df)

res = mod.fit()

print(res.summary())

# If any data points have residuals > 0.5, remove these.

if np.any(np.sqrt(res.resid*res.resid) > 0.5):

df = df.drop(df.ix[np.sqrt(res.resid*res.resid) > 0.5,:].index)

mod = smf.ols(formula='HR ~ Ind', data=df)

res = mod.fit()

# Get predicted HR values

# predictedHRs = res.predict(np.arange(24))

predictedHRs = self.res.predict(pd.DataFrame(data = np.arange(24), columns = ['Ind']))

# Get intercept and gradient

plt.figure()

p = mod.fit().params

print 'Intercept: ' + str(p[0]) + '; Slope: ' + str(p[1])

plt.plot(df.ix[:,'Ind'],df.ix[:,'HR'],'o')

plt.plot(df.ix[:,'Ind'], p[0] + p[1] * df.ix[:,'Ind'],color='black')

plt.ylim(0,df.ix[:,'HR'].max()*1.2)

zip(np.arange(0, len(self.results['dateTime'].values[23:])),self.results['dateTime'].values,self.results['Metadata'].to_pandas().ix[23:,0,'currentFolder'],HRs[23:],predictedHRs)

self.heartRate_data[str(self.embryo)] = {'Embryo': self.embryo,'PredHR':predictedHRs, 'Intercept': str(p[0]), 'Slope':str(p[1]),

'modPars_filtMin':filtMin,'modPars_filtMax':filtMax,'modPars_histCount':histCount,'modPars_peakThresh':peakThresh}

except:

print '*** Heart rate not succesfully modelled for ' + str(self.embryo) + ' ***'

#==============================================================================

# Umbrella function for measuring heart rate - Radix balthica

#==============================================================================

def measureHeartRate_radix(self,savePath,filtMin=1,filtMax=2,histCount=6,segRegfiltXVal=5,segRegfiltYVal=6,minXVal=False,peakThresh=0.4):

""" Attempt to extract and model heart rate from 8x8 blockwise frequency data.

Note that some user guidance is recommended and if the heart rate cannot be succesfully

modelled try changing some of these optional arguments:

# Provide a folder for saving output plots and statistics.

# The minimum acceptable HR (Hz)

filtMin=1

# The maximum acceptable HR (Hz)

filtMax=2

# The number of signals in which a frequency must occur as being dominant to be considered.

histCount=6

# The extent to which outlying values are filtered in the X dimension prior to fitting a segmented regression.

segRegfiltXVal=4

# The extent to which outlying values are filtered in the Y dimension prior to fitting a segmented regression.

segRegfiltYVal=6

# The threshold for identifying peaks in the frequency output

peakThresh=0.4

"""

#if 'heartRate_data' not in locals():

if not hasattr(self,'heartRate_data'):

self.heartRate_data = dict()

if self.results is 'NoData':

print 'No data for ' + str(self.embryo)

else:

self.freqRes = self.results['FreqOutput_8x8'].values

hrvals = []

gs, fs = np.meshgrid(range(8),range(8))

gs = gs.flatten()

fs = fs.flatten()

for i in range(self.freqRes.shape[0]):

hrvals.append(self.modelHR_radix(gs,fs,i,filtMin,filtMax,histCount,minXVal,peakThresh))

self.test = hrvals

#try:

self.predHR, self.predBP, self.BP, self.S1,self.S2,self.startTime,self.endTime,self.firstHR,self.lastHR,self.HRData,self.outputSummary = self.segReg_radix(hrvals,segRegfiltXVal,segRegfiltYVal)

print 'making it to here'

# Show fit

fig, ax = plt.subplots(1,1)

ax.plot(self.predHR)

ax.plot(self.HRData,'o')

#ax.plot(sortedTimes, sortedPredicted)

#ax.plot(times, np.array(HRData)[~np.isnan(HRData)], 'o')

plt.title(str(self.embryo))

plt.show()

plt.xlabel('Time point')

plt.ylabel('Frequency (Hz)')

plt.ylim(0,(np.nanmax(np.array((self.predHR,self.HRData)))*1.2))

fig.subplots_adjust(bottom=0.3)

fig.text(.1,.1,str(self.outputSummary[0] + '\n' + self.outputSummary[1] + '\n' + self.outputSummary[2]))

fig.canvas.manager.window.raise_()

pdf_pages = PdfPages(savePath + self.embryo + '_heartModelSummary.pdf')

pdf_pages.savefig(fig)

pdf_pages.close()

plt.plot()

print 'plotting is ok'

#time.sleep(0.2)

# Add data to a dict - new key for each embryo

self.heartRate_data[str(self.embryo)] = {'Embryo': self.embryo,'PredHR':self.predHR,

'PredBP':self.predBP, 'BP': self.BP, 'S1': self.S1,'S2':self.S2,'startTime':self.startTime,

'endTime':self.endTime,'firstHR':self.firstHR,'lastHR':self.lastHR,'HRData':self.HRData,

'modPars_filtMin':filtMin,'modPars_filtMax':filtMax,'modPars_histCount':histCount,'modPars_segRegfiltXVal':segRegfiltXVal,

'modPars_segRegfiltYVal':segRegfiltYVal,'modPars_minXVal':minXVal,'modPars_peakThresh':peakThresh}

#except:

# print '*** Heart rate not succesfully modelled for ' + str(self.embryo) + ' ***'

#==============================================================================

# Model HR for Radix balthica

#==============================================================================

# Extract peaks from the freq output that may be HR.

def modelHR_radix(self,gs,fs, t,filtMin, filtMax, histCount, minXVal,peakThresh):

""" Called from measureHeartRate_radix().

"""

HRFreqs=[]

# If no minVal (time point) provided, or the t value is above the minimum value

# attempt to ID the heart rate.

if type(minXVal) is not bool and minXVal<= t or not minXVal:

if not math.isnan(self.freqRes[t,0,0,1,0]):

for b in range(len(gs)):

powerSpect = self.freqRes[t,fs[b],gs[b],1,:]

if (powerSpect.max() == 0.0):

HRFreqs.append(np.NaN)

else:

sampFreqs = self.freqRes[t,fs[b],gs[b],0,:]

try:

baselineRemoved = np.log(powerSpect) - peakutils.baseline(np.log(powerSpect)- min(np.log(powerSpect)))

# Now ID peaks.anan

indexes = peakutils.indexes(baselineRemoved, peakThresh, min_dist=1)

outPeakFreqs = sampFreqs[indexes]

# And get magnitudes (may be useful for downstream filtering).

outMagnitudes = baselineRemoved[indexes]

# Identify index(or indices)

# Gam

#inds = np.where(((outPeakFreqs < 5) & (outPeakFreqs > 0.5)))

# Rad

#inds = np.where(((outPeakFreqs < 2) & (outPeakFreqs > 1)))

inds = np.where(((outPeakFreqs < filtMax) & (outPeakFreqs > filtMin)))

filtFreqs = outPeakFreqs[inds]

filtPower = baselineRemoved[inds]

# If more than one freq peak identified within the range 0.5-3 Hz

if inds[0].shape[0] > 1:

#print str(b)

# Identify freq with most power

maxInd = np.argmax(filtPower)

HRFreq = filtFreqs[maxInd]

HRFreqs.append(HRFreq)

elif inds[0].shape[0] == 1:

HRFreq = outPeakFreqs[inds[0][0]]

HRFreqs.append(HRFreq)

else:

HRFreqs.append(np.NaN)

except:

HRFreqs.append(np.NaN)

else:

HRFreqs.append(np.NaN)

HRFreqs = np.array(HRFreqs)

# Remove NaNs

count, freqs = np.histogram(HRFreqs[np.invert(np.isnan(HRFreqs))],bins=50)

# Identify whether a sufficiently dominant frequency to reliably ID HR.

inds = np.where(count > histCount)

if len(inds[0]) is not 0:

# Get max ind

maxInds = inds[-1]

HR = np.mean(freqs[maxInds])

else:

HR = np.NaN

else:

HR = np.NaN

else:

# If current time iteration is below the min value (time) assign NaN.

HR = np.NaN

return HR

#==============================================================================

# HR - fits a segmented regression using R.

# Used to model HR for R. balthica

#==============================================================================

def segReg_radix(self,HRData,filtXVal,filtYVal):

# Fit segmented regressionpN in R to the HR data. Note various filtering done here..

base = importr('base')

r = robjects.r

seg = importr('segmented')

# Filter data to remove lone x axis

# ID where values exist

filt = ~np.isnan(HRData)

# Use histogram to ID outlier

count, bins = np.histogram(np.array(range(0,len(HRData)))[filt])

# Filter to remove outlying x values

filtInd = bins[:-1][(count > 0) & (count < filtXVal)]

filtInds = np.nonzero([(count > 0) & (count < filtXVal)])[1]

# If any time points to filter

HRData = np.array(HRData)

if filtInd.shape[0] is not 0:

#HRData[filtInd.astype(np.int)] = np.NaN

# Add filter to remove any values within particular bin requiring filtering..

for i in range(len(filtInds)):

for t in range(len(HRData)):

if (t >= bins[filtInds][i]) & (t <= bins[filtInds[i]+1]):

# Debug

# print 'X filtered'

HRData[t] = np.NaN

# Filter to remove outlying y values

Yax, bins = np.histogram(HRData[~np.isnan(HRData)])

filtInd = Yax < filtYVal

filtInds = np.nonzero([Yax < filtYVal])[1]

if filtInds.shape[0] is not 0:

#Yax[1][1:][Yax[0] < 2]

for i in range(len(filtInds)):

for t in range(len(HRData)):

if (HRData[t] >= bins[filtInds][i]) & (HRData[t] <= bins[filtInds[i]+1]):

#print 'success'

HRData[t] = np.NaN

# Format/convert data

HR = robjects.FloatVector(HRData)

Time = robjects.FloatVector(range(0,len(HRData)))

robjects.globalenv["HR"] = HR

robjects.globalenv["Time"] = Time

# Run linear model

lmHR = r.lm("HR ~ Time")

#print(base.summary(lmHR))

# Model for seg.z

formula = robjects.Formula("~Time")

# Also use mean of data containing values to get starting psi

startingPSI = np.int(np.median(np.arange(0,len(HRData))[~np.isnan(HRData)]))

# Run segmented model and print results

segModHR = seg.segmented(lmHR, seg_Z=formula, psi=startingPSI, model=True)

#print(seg.summary_segmented(segModHR, short = True))

# print(seg.print.segmented(segModHR))

# Plotting segmented in Python

resultsDict = dict(zip(segModHR.names, list(segModHR)))

breakPoint = resultsDict['psi'][1]

# Get fitted values

predicted = np.array(seg.predict_segmented(segModHR))

times = np.arange(0,len(HRData))[~np.isnan(HRData)]

#plt.plot(times, np.array(HRData)[~np.isnan(HRData)], 'o')

# Get slopes

slopes = seg.slope(segModHR)

slopes = np.array(slopes[0])

slopeOne = slopes[0,0]

slopeTwo = slopes[1,0]

#print(seg.summary_segmented(segModHR))

self.out = seg.summary_segmented(segModHR)

self.tmp = segModHR.items

# Get fitted values for all times (predicted values above skip missing data)

# Create an R dataframe with x-values to predict from

d = {'Time': robjects.FloatVector(np.arange(times.min(),times.max()))}

dataf = robjects.DataFrame(d)

# Predict values - all times (although when plotting in Matplotlib the breakpoint will appear slightly flattened)

predictedVals = r.predict(segModHR, newdata = dataf)

#plt.plot(np.arange(times.min(),times.max()),np.array(predictedVals))

# Get fitted value for breakpoint - this must be used in matplotlib plotting for break to appear appropriately

d = {'Time': robjects.FloatVector([breakPoint])}

breakpointTime = robjects.DataFrame(d)

pyBreakpointTime = np.array(breakpointTime)[0][0]

# Predict values

predictedBreakPointVal = np.array(r.predict(segModHR, newdata = breakpointTime))

#plt.plot(np.append(np.arange(times.min(),times.max()),breakPoint),np.append(predictedVals,predictedBreakPointVal), 'o')

# Sort to show segmented regression including appropriate breakpoint.

timeSort = np.append(np.arange(times.min(),times.max()),breakPoint)

sortedTimes = timeSort[np.argsort(timeSort)]

predicted = np.append(predictedVals,predictedBreakPointVal)

sortedPredicted = predicted[np.argsort(timeSort)]

# Pad predicted values with NaNs to shift appropriately.

startTime = np.min(np.where((~np.isnan(HRData))))

endTime = np.max(np.where((~np.isnan(HRData))))

#endHR = np.argmax(~np.isnan(HRData[::-1]))

predictedHRArray = np.zeros(len(HRData))

predictedHRArray[:] = np.NaN

predictedHRArray[times.min():times.max()] = predictedVals

# Get first predicted rate

firstHR = predictedHRArray[np.where(~np.isnan(predictedHRArray))[0][0]]

# Get last predicted rate

lastHR = predictedHRArray[np.where(~np.isnan(predictedHRArray))[0][-1]]

# Get rate at breakpoint

#breakpointHR = predictedHRArray[np.int(breakPoint)]

print 'Heart rate modelling for: ', str(self.embryo)

print 'Breakpoint HR: ', str(predictedBreakPointVal[0]), ', Slope 1: ', str(slopeOne), ', Slope 2: ', str(slopeTwo)

print 'Heart rate detected: ', str(startTime), 'Breakpoint occured: ', str(pyBreakpointTime), 'Heart rate no longer detected: ', str(endTime)

print 'Start heart rate: ', str(firstHR), ', End heart rate: ', str(lastHR)

outputSummary = [str('Heart rate modelling for: '+ str(self.embryo)),

str('Breakpoint HR: '+ str(predictedBreakPointVal[0])+ ' Slope 1: '+ str(slopeOne)+ ' Slope 2: '+ str(slopeTwo)),

str('Start heart rate: '+ str(firstHR)+ ' End heart rate: '+ str(lastHR))]

return list(predictedHRArray), list(predictedBreakPointVal), pyBreakpointTime, slopeOne, slopeTwo, startTime, endTime, firstHR, lastHR, list(HRData),outputSummary

def identifyLethalEndPoints(self, savePath, developmentalStage):

"""

Identify lethal end points. Currently optimised and tested for three

developmental stages of Radix balthica, employing different strategies

for each (drops in frequency energy, peaks in size indicative of a

failure in osmotic control and a combination of both).

"""

# If developmentalStage is late hippo identify drops in frequency energy

if developmentalStage is 'latehippo':

self.lethalEndPoint_data = dict()

for e in range(len(self.embryoLabels)):

self.embryo = self.embryoLabels[e]

self.loadXRResults()

# Extract required data from TimeSpecificSummaryData component of XArray dataset

totalLower = np.nansum(self.results['TimeSpecificSummaryData'].to_pandas().ix[:72,13:17],axis=1)

avHigher = np.nanmean(self.results['TimeSpecificSummaryData'].to_pandas().ix[:72,18:],axis=1)

# Normalise - this seems to work well.

data = pd.DataFrame(totalLower/avHigher).interpolate(limit_direction = 'both').values.ravel()

data = pd.DataFrame(data).interpolate(limit_direction = 'both').values.ravel()

# Identify where energy levels fall below threshold - likely to require diff

# thresholds for diff stages etc - first filter.

inds = np.where(np.log(data) < 3)

# If more than one drop identified - take just the first (falling below)

if len(inds[0]) > 1:

lethalIndex = np.min(inds[0])

lethalTime = self.results['TimeSpecificSummaryData'].to_pandas().index[lethalIndex]

self.lethalEndPoint_data[str(self.embryo)] = {'Embryo': self.embryo,'Dev stage': str(developmentalStage),'LethalTime': lethalTime,'LethalIndex': lethalIndex}

else:

# Identify where energy levels fall below threshold - likely to require diff

# thresholds for diff stages etc - second filter.

inds = np.where(np.log(data) < 3.3)

if len(inds[0]) > 1:

lethalIndex = np.min(inds[0])

lethalTime = self.results['TimeSpecificSummaryData'].to_pandas().index[lethalIndex]

self.lethalEndPoint_data[str(self.embryo)] = {'Embryo': self.embryo,'Dev stage': str(developmentalStage),'LethalTime': lethalTime,'LethalIndex': lethalIndex}

else:

lethalIndex = np.NaN

self.lethalEndPoint_data[str(self.embryo)] = {'Embryo': self.embryo,'Dev stage': str(developmentalStage),'LethalTime': np.NaN,'LethalIndex': lethalIndex}

np.save(savePath + '/lethalEndPoints.npy', self.lethalEndPoint_data)

# If developmentalStage is mid hippo first attempt to identify drops in frequency energy

# And failing this try peaks in size indicative of a failure in osmotic control.

if developmentalStage is 'midhippo':

self.lethalEndPoint_data = dict()

for e in range(len(self.embryoLabels)):

self.embryo = self.embryoLabels[e]

self.loadXRResults()

# Extract required data from TimeSpecificSummaryData component of XArray dataset

totalLower = np.nansum(self.results['TimeSpecificSummaryData'].to_pandas().ix[:,13:17],axis=1)

avHigher = np.nanmean(self.results['TimeSpecificSummaryData'].to_pandas().ix[:,18:],axis=1)

# Normalise - this seems to work well.

#data = pd.DataFrame(totalLower/avHigher).interpolate(limit_direction = 'both').values.ravel()

#data = pd.DataFrame(data).interpolate(limit_direction = 'both').values.ravel()

# Identify where energy levels fall below threshold - likely to require diff

# thresholds for diff stages etc - first filter.

inds = np.where(np.log(totalLower/avHigher) < 3)

# If more than one drop identified - take just the first (falling below)

if len(inds[0]) > 1:

lethalIndex = np.min(inds[0])

lethalTime = self.results['TimeSpecificSummaryData'].to_pandas().index[lethalIndex]

self.lethalEndPoint_data[str(self.embryo)] = {'Embryo': self.embryo,'Dev stage': str(developmentalStage),'LethalTime': lethalTime,'LethalIndex': lethalIndex}

#print str(lethalIndex)

else:

self.lethalEndPoint_data[str(self.embryo)] = {'Embryo': self.embryo,'Dev stage': str(developmentalStage),'LethalTime': np.NaN,'LethalIndex': np.NaN}

# If no lethal end point has been found also check for peaks in embryo size

# indicative of a loss of osmotic control

for e in range(len(self.embryoLabels)):

self.embryo = self.embryoLabels[e]

self.loadXRResults()

if self.lethalEndPoint_data[str(self.embryo)]['LethalTime'] is np.NaN:

data = pd.rolling_mean(self.results['TimeSpecificSummaryData'].to_pandas().ix[:,0],window=6)

if np.count_nonzero(np.isnan(data))/np.float(len(data)) > 0.7:

self.lethalEndPoint_data[str(self.embryo)] = {'Embryo': self.embryo,'Dev stage': str(developmentalStage),'LethalTime': np.NaN,'LethalIndex': np.NaN}

else:

# Interpolate to fill missing data

data = pd.DataFrame(data).interpolate(limit_direction = 'both').values.ravel()

#print np.count_nonzero(np.isnan(data))

# Identify peaks/loss of embryo osmotic control

indexes = peakutils.indexes(data-peakutils.baseline(data), thres=0.99, min_dist=1)

if len(indexes) == 1:

# Check that the peak is sufficiently distant from the baseline..

# Assess proportionate distance of peak from baseline.

baseline = peakutils.baseline(data)

propDist = np.abs(baseline-data[indexes]).min()/np.mean(baseline)

#print propDist

if propDist < 0.2:

self.lethalEndPoint_data[str(self.embryo)] = {'Embryo': self.embryo,'Dev stage': str(developmentalStage),'LethalTime': np.NaN,'LethalIndex': np.NaN}

else:

lethalTime = self.results['TimeSpecificSummaryData'].to_pandas().index[indexes[0]]

lethalIndex = indexes[0]

self.lethalEndPoint_data[str(self.embryo)] = {'Embryo': self.embryo,'Dev stage': str(developmentalStage),'LethalTime': lethalTime,'LethalIndex': lethalIndex}

#print str(lethalIndex)

#print 'Lethal end point identified via peak in area'

if len(indexes) > 1:

baseline = peakutils.baseline(data)

index = np.argmax(data[indexes])

# Check that the peak is sufficiently distant from the baseline..

# Assess proportionate distance of peak from baseline.

propDist = np.abs(baseline-data[indexes][index]).min()/np.mean(baseline)

print propDist

if propDist < 0.2:

#print 'No peak found'

self.lethalEndPoint_data[str(self.embryo)] = {'Embryo': self.embryo,'Dev stage': str(developmentalStage),'LethalTime': np.NaN,'LethalIndex': np.NaN}

else:

#mode[e] = 'ar'

lethalTime = self.results['TimeSpecificSummaryData'].to_pandas().index[index]

lethalIndex =index

self.lethalEndPoint_data[str(self.embryo)] = {'Embryo': self.embryo,'Dev stage': str(developmentalStage),'LethalTime': lethalTime,'LethalIndex': lethalIndex}

#print str(lethalIndex)

#print 'Lethal end point identified via peak in area'

if len(indexes) < 1:

self.lethalEndPoint_data[str(self.embryo)] = {'Embryo': self.embryo,'Dev stage': str(developmentalStage),'LethalTime': np.NaN,'LethalIndex': np.NaN}

#print 'Cant be sure'

# If developmentalStage is trochophore identify drops in frequency energy

if developmentalStage is 'trochophore':

self.lethalEndPoint_data = dict()

for e in range(len(self.embryoLabels)):

self.embryo = self.embryoLabels[e]

self.loadXRResults()

data = pd.rolling_mean(self.results['TimeSpecificSummaryData'].to_pandas().ix[:,0],window=2)

if np.count_nonzero(np.isnan(data))/np.float(len(data)) > 0.7:

print 'Not enough data for ' + str(self.embryo)

self.lethalEndPoint_data[str(self.embryo)] = {'Embryo': self.embryo,'Dev stage': str(developmentalStage),'LethalTime': np.NaN,'LethalIndex': np.NaN}

else:

# Interpolate to fill missing data

data = pd.DataFrame(data).interpolate(limit_direction = 'both').values.ravel()

# Identify peaks/loss of embryo osmotic control

indexes = peakutils.indexes(data-peakutils.baseline(data), thres=0.9, min_dist=1)

if len(indexes) == 1:

# Check that the peak is sufficiently distant from the baseline..

# Assess proportionate distance of peak from baseline.

baseline = peakutils.baseline(data)

propDist = np.abs(baseline-data[indexes]).min()/np.mean(baseline)

if propDist < 0.1:

self.lethalEndPoint_data[str(self.embryo)] = {'Embryo': self.embryo,'Dev stage': str(developmentalStage),'LethalTime': np.NaN,'LethalIndex': np.NaN}

else:

#lethalIndex = indexes[0]-1

lethalIndex = indexes[0]-1

lethalTime = self.results['TimeSpecificSummaryData'].to_pandas().index[lethalIndex]

self.lethalEndPoint_data[str(self.embryo)] = {'Embryo': self.embryo,'Dev stage': str(developmentalStage),'LethalTime': lethalTime,'LethalIndex': lethalIndex}

if len(indexes) > 1:

baseline = peakutils.baseline(data)

index = np.argmax(data[indexes])

# Check that the peak is sufficiently distant from the baseline..

# Assess proportionate distance of peak from baseline.

propDist = np.abs(baseline-data[indexes][index]).min()/np.mean(baseline)

if propDist < 0.1:

self.lethalEndPoint_data[str(self.embryo)] = {'Embryo': self.embryo,'Dev stage': str(developmentalStage),'LethalTime': np.NaN,'LethalIndex': np.NaN}

else:

lethalIndex = indexes[index]-1

#lethalIndex = indexes[index]-1

lethalTime = self.results['TimeSpecificSummaryData'].to_pandas().index[lethalIndex]

self.lethalEndPoint_data[str(self.embryo)] = {'Embryo': self.embryo,'Dev stage': str(developmentalStage),'LethalTime': lethalTime,'LethalIndex': lethalIndex}

if len(indexes) < 1:

self.lethalEndPoint_data[str(self.embryo)] = {'Embryo': self.embryo,'Dev stage': str(developmentalStage),'LethalTime': np.NaN,'LethalIndex': np.NaN}

# Visualise and save output

for keys,values in sorted(self.lethalEndPoint_data.items()):

print(keys)

print(values)

np.save(savePath + '/lethalEndPoints.npy', self.lethalEndPoint_data)

**dataIntegration.py**

import cv2

import numpy as np

import matplotlib.pyplot as plt

import pandas as pd

import time

import pathos

import scipy

import scipy.fftpack

import xarray as xr

from scipy import stats

import scipy.signal as signal

import math

import warnings

import shutil

import glob

import numpy as np

class dataIntegration(object):

"""

This class generates summary stats from XArray Datasets and adds these to

the DataSet file. Two types of stats are generated:

- global (statistics relevant to the entire period of the experiment

e.g. growth rates)

- timeSpecific (e.g. specific to a particular time point). The length of

timeSpecifc stats is equal to the duration of the experiment, with missing

values where either no data was present or stats cannot be calculated for

some reason.

"""

#==============================================================================

#%%

#==============================================================================

# Apply calculatePhenomeMeasures to all embryos

#==============================================================================

def savePhenomeMeasuresForAllEmbryos(self, savePath,ignoreMeta = False):

# Note can add ignoreMeta = True if metadata does not need to be copied to new dataset folder.

# This can be useful if the results folder and file structure is abnormal.

if not ignoreMeta:

shutil.copy2(str(glob.glob(self.parentPath + "*.npy")[0]), savePath + "phenomeMetadata.npy")

print self.embryoLabels

for e in range(len(self.embryoLabels)):

self.calculatePhenomeMeasures(e, savePath)

#==============================================================================

# Integrate data to i)Produce phenome measures, ii)Save as XArray dataset and iii)Produce summary reports

#==============================================================================

def calculatePhenomeMeasures(self, embryo, savePath):

warnings.filterwarnings('ignore','All-NaN slice encountered')

warnings.filterwarnings('ignore','Mean of empty slice')

# Load results

ts = time.time()

self.embryo = self.embryoLabels[embryo]

self.loadResults()

print 'Loading ' + self.embryoLabels[embryo]

# If no/little data/tracking...

if np.isnan(np.nanmean(self.results.ix[:,:,'area'], axis=0).astype(np.float64)).all():

print self.embryoLabels[embryo] + ' contains no embryo data.'

print 'This is most likely to have occured due to it not being located succesfully.'

print 'Consequently no results file will be produced, but a .log file will be to serve as a record.'

with open(savePath + self.embryoLabels[embryo] + 'dataset.HDF5_log', 'w') as f:

f.write('No data for ' + self.embryoLabels[embryo] + ".")

savePath + self.embryoLabels[embryo] + 'dataset.HDF5_log'

print self.embryoLabels[embryo] + "dataset.HDF5_log saved"

elif np.sum(~np.isnan(np.nanmean(self.results.ix[:,:,'area'], axis=0).astype(np.float64))) < 5:

print self.embryoLabels[embryo] + ' contains little embryo data (less than 5 time points).'

print 'This is most likely to have occured due to it not being located succesfully.'

print 'Consequently no results file will be produced, but a .log file will be to serve as a record.'

with open(savePath + self.embryoLabels[embryo] + 'dataset.HDF5_log', 'w') as f:

f.write('Less than 5 time points containing data for ' + self.embryoLabels[embryo] + ".")

savePath + self.embryoLabels[embryo] + 'dataset.HDF5_log'

print self.embryoLabels[embryo] + "dataset.HDF5_log saved"

else:

### Extract BLOCKWISE data and store as XArray

blockWiseVals = self.results.ix[:,:,'blockWise'].values

blockWiseArrayOne = np.zeros((blockWiseVals.shape[1],blockWiseVals.shape[0]))

blockWiseArrayTwo = np.zeros((blockWiseVals.shape[1],blockWiseVals.shape[0],2,2))

blockWiseArrayThree = np.zeros((blockWiseVals.shape[1],blockWiseVals.shape[0],4,4))

blockWiseArrayFour = np.zeros((blockWiseVals.shape[1],blockWiseVals.shape[0],8,8))

blockWiseArrayFive = np.zeros((blockWiseVals.shape[1],blockWiseVals.shape[0],16,16))

blockWiseArrayFive = np.ascontiguousarray(blockWiseArrayFive)

for t in np.arange(blockWiseVals.shape[1]):

for f in range(blockWiseVals.shape[0]):

if (type(blockWiseVals[f,t]) is list):

# Res 1x1

blockWiseArrayOne[t,f] = blockWiseVals[f,t][0]

# Res 2x2 : 16x16

blockWiseArrayTwo[t,f,:,:] = np.array(blockWiseVals[f,t][1])

blockWiseArrayThree[t,f,:,:] = np.array(blockWiseVals[f,t][2])

blockWiseArrayFour[t,f,:,:] = np.array(blockWiseVals[f,t][3])

blockWiseArrayFive[t,f,:,:] = np.array(blockWiseVals[f,t][4])

else:

blockWiseArrayOne[t,f] = np.NaN

blockWiseArrayTwo[t,f,:,:] = np.NaN

blockWiseArrayThree[t,f,:,:] = np.NaN

blockWiseArrayFour[t,f,:,:] = np.NaN

blockWiseArrayFive[t,f,:,:] = np.NaN

self.blockWiseArrayOneXR = xr.DataArray(blockWiseArrayOne)

self.blockWiseArrayTwoXR = xr.DataArray(blockWiseArrayTwo)

self.blockWiseArrayThreeXR = xr.DataArray(blockWiseArrayThree)

self.blockWiseArrayFourXR = xr.DataArray(blockWiseArrayFour)

self.blockWiseArrayFiveXR = xr.DataArray(blockWiseArrayFive)

### Generate FREQ data from Blockwise data and output as XArray.

# Create output arrays and info necessary to make them

frameRate = self.results.shape[1]/((np.float(self.results.ix[0,self.results.shape[1]-1,'elapsedTime'])-np.float(self.results.ix[0,0,'elapsedTime']))/1000)

sampFreqs, powerSpect = signal.welch(self.blockWiseArrayOneXR.values[0,:],frameRate, scaling='spectrum', nfft=self.blockWiseArrayOneXR.shape[1])

# Numpy arrays for other resolutions

self.freqOutputOne = np.zeros((self.results.shape[0],2,len(powerSpect)))

self.freqOutputTwo = np.zeros((self.results.shape[0],2,2,2,len(powerSpect)))

self.freqOutputThree = np.zeros((self.results.shape[0],4,4,2,len(powerSpect)))

self.freqOutputFour = np.zeros((self.results.shape[0],8,8,2,len(powerSpect)))

self.freqOutputFive = np.zeros((self.results.shape[0],16,16,2,len(powerSpect)))

# Create a threading pool - limited to 12 as the default (on MacPro) cripples the computer (> 60GB RAM used).

innerPool = pathos.multiprocessing.ThreadingPool(6)

# Run freq analysis

res = innerPool.map(self.parFreqAnalysis, range(self.freqOutputOne.shape[0]))

# Unpack results

for t in xrange(self.freqOutputOne.shape[0]):

self.freqOutputOne[t,:,:] = res[t]['F1'][:,:]

self.freqOutputTwo[t,:,:,:,:] = res[t]['F2'][:,:,:,:]

self.freqOutputThree[t,:,:,:,:] = res[t]['F3'][:,:,:,:]

self.freqOutputFour[t,:,:,:,:] = res[t]['F4'][:,:,:,:]

self.freqOutputFive[t,:,:,:,:] = res[t]['F5'][:,:,:,:]

# Store as XArrays

self.freqOutputOneXR = xr.DataArray(self.freqOutputOne)

self.freqOutputTwoXR = xr.DataArray(self.freqOutputTwo)

self.freqOutputThreeXR = xr.DataArray(self.freqOutputThree)

self.freqOutputFourXR = xr.DataArray(self.freqOutputFour)

self.freqOutputFiveXR = xr.DataArray(self.freqOutputFive)

### Extract METADATA

res = self.results

meta = res.drop(['dateTime', 'elapsedTime', 'area', 'centroidX', 'centroidY',

'solidity', 'aspect', 'extent', 'hullArea', 'bboxMincol',

'bboxMinrow', 'bboxWidth', 'bboxHeight', 'embryoOutline',

'eggRotBBox', 'eggBoxPoints', 'blockWise'], axis=2)

# Extract 'size & pos' data

sizePos = self.results.ix[:,:,7:18].astype('float')

sizePosArray = xr.DataArray(sizePos)

# Extract ROTATED EGG BBOX

eggRotBB = self.results.ix[:,:,'eggRotBBox'].values

eggRotBBArray = np.zeros((self.results.shape[1],self.results.shape[0],5))

for t in range(self.results.shape[0]):

for f in range(self.results.shape[1]):

if type is list:

if (not math.isnan(float(eggRotBB[f,t][0]))):

eggRotBBArray[f,t,:] = eggRotBB[f,t]

else:

eggRotBBArray[f,t,:] = np.NaN

eggRotBBArray = eggRotBBArray.swapaxes(0,1)

eggRotBBXR = xr.DataArray(eggRotBBArray,{'Items':self.results.items,'Frame':self.results.major_axis,'EggRotBBox':['X','Y','W','H','A']})

# Extract SIZE AND POS data

sizePos = self.results.ix[:,:,7:18].astype('float')

sizePosArray = xr.DataArray(sizePos)

# Generate SUMMARY STATS

timeSpStats, globalStats = self.generateSummaryStats()

# NOW COMBINE and SAVE

dateTime = self.results.ix[:,0,'dateTime']

testDataSet = xr.Dataset({'Metadata':(['dateTime','frame','meta'],meta),

'SizePos':(['dateTime','frame','sizePos'],sizePosArray),

'EggBB':(['dateTime','frame','point','dim'],self.eggBBArray),

'EmbryoOutline':(['dateTime','frame','dim','coord'],self.embryoOutlineXR),

'EggRotBBox':(['dateTime','frame','eggRotBBox'],eggRotBBXR),

'BlockWise_1x1':(['dateTime','frame'],self.blockWiseArrayOneXR),

'BlockWise_2x2':(['dateTime','frame','X_2x2','Y_2x2'],self.blockWiseArrayTwoXR),

'BlockWise_4x4':(['dateTime','frame','X_4x4','Y_4x4'],self.blockWiseArrayThreeXR),

'BlockWise_8x8':(['dateTime','frame','X_8x8','Y_8x8'],self.blockWiseArrayFourXR),

'BlockWise_16x16':(['dateTime','frame','X_16x16','Y_16x16'],self.blockWiseArrayFiveXR),

'FreqOutput_1x1':(['dateTime','freq/Power','freqs'],self.freqOutputOneXR),

'FreqOutput_2x2':(['dateTime','X_2x2','Y_2x2','freqPower','freqs'],self.freqOutputTwoXR),

'FreqOutput_4x4':(['dateTime','X_4x4','Y_4x4','freqPower','freqs'],self.freqOutputThreeXR),

'FreqOutput_8x8':(['dateTime','X_8x8','Y_8x8','freqPower','freqs'],self.freqOutputFourXR),

'FreqOutput_16x16':(['dateTime','X_16x16','Y_16x16','freqPower','freqs'],self.freqOutputFiveXR),

'TimeSpecificSummaryData':(['dateTime','timeSpecificMeasure'],timeSpStats.T),

'GlobalSummaryData':(['globalMeasure', 'value'],xr.DataArray(pd.DataFrame(data = globalStats.values(), index= globalStats.keys())))},

coords={'dateTime':dateTime,

'frame':(list(np.arange(0,self.results.shape[1]))),

'meta':(meta.minor_axis.values),

'sizePos':(sizePosArray.coords[('dim_2')].values),

'dim':['X','Y'],

'point':['0','1','2','3'],

'freqs':(list(np.arange(0,self.freqOutputFive.shape[4]))),

'timeSpecificMeasure':['meanArea', 'minArea', 'maxArea','meanMinBB','meanMaxBB',

'minMinBB','maxMaxBB','meanDistance','minDistance','maxDistance',

'totalDistance','meanEggLength', 'meanEggWidth','Freq0-0.1Hz',

'Freq0.1-0.3Hz','Freq0.3-0.5Hz','Freq0.5-0.7Hz',

'Freq0.7-0.9Hz','Freq0.9-1.2Hz','Freq1.2-1.6Hz','Freq1.6-1.8Hz',

'Freq1.8-2.2Hz','Freq2.2-3.0Hz','Freq3.0-4.0Hz','Freq4.0-5.0Hz',

'Freq5.0Hz-'],

'globalMeasure':['MeanArGrowthRateSlope','MeanArGrowthRateIntercept','MeanArGrowthR',

'MeanArGrowthRatePval', 'MeanArGrowthSE','MinArGrowthRateSlope',

'MinArGrowthRateIntercept','MinArGrowthR','MinArGrowthRatePval',

'MinArGrowthSE','MaxArGrowthRateSlope','MaxArGrowthRateIntercept',

'MaxArGrowthR','MaxArGrowthRatePval','MaxArGrowthSE','MeanMinLenGrowthRateSlope',

'MeanMinLenGrowthRateIntercept','MeanMinLenGrowthR','MeanMinLenGrowthRatePval',

'MeanMinLenGrowthSE','MinMinLenGrowthRateSlope','MinMinLenGrowthRateIntercept',

'MinMinLenGrowthR','MinMinLenGrowthRatePval','MinMinLenGrowthSE',

'MaxMaxLenGrowthRateSlope','MaxMaxLenGrowthRateIntercept',

'MaxMaxLenGrowthR','MaxMaxLenGrowthRatePval','MaxMaxLenGrowthSE']})

testDataSet.to_netcdf(savePath + self.embryoLabels[embryo] + 'dataset.HDF5')

print 'Saving ' + self.embryoLabels[embryo] + ' XArray Datset'

print 'Phenome measures generated for ' + self.embryoLabels[embryo] + ' in {} s'.format(time.time()-ts)

#%%

#==============================================================================

# Generate summary stats (called from calculatePhenomeMeasures)

#==============================================================================

def generateSummaryStats(self):

# Remove points containing no data..

minDistance = []

maxDistance = []

meanDistance = []

totalDistance = []

meanMinBB = []

meanMaxBB = []

minMinBB = []

maxMaxBB = []

mins = []

maxs = []

eggLength = []

eggWidth = []

meanEggLength = []

meanEggWidth = []

# EMBRYO OUTLINE

outlineVals = self.results.ix[:,:,'embryoOutline'].values

arrLen = 10000

lengths=[]

outlineArray = np.zeros((self.results.shape[1],self.results.shape[0],2,arrLen))

for t in range(self.results.shape[0]):

for f in range(self.results.shape[1]):

if (type(outlineVals[f,t]) is np.ndarray):

length = len(outlineVals[f,t][:,0,0])

lengths.append(length)

if length < arrLen:

outlineArray[f,t,0,0:length] = outlineVals[f,t][:,0,0]

outlineArray[f,t,1,0:length] = outlineVals[f,t][:,0,1]

else:

print 'Too many pixel coords for array'

else:

outlineArray[f,t,:,:] = np.NaN

# Reshape (time, frames..)

outlineArray = outlineArray.swapaxes(0,1)

# Catch instances when no embryo outlines are present

if (len(lengths) > 0):

# Crop to the maximum number of coordinates

outlineArray = outlineArray[:,:,:,0:max(lengths)]

else:

outlineArray = outlineArray[:,:,:,0:1]

outlineArray[:,:,:,:] = np.NaN

# For some reason (prob simple mistake) cannot create a labelled XArray with these pixe data, however the default (non-labelled) creation below works fine.

#embryoOutlineXR = xr.DataArray(outlineArray, {'Items':self.results.items,'Frame':self.results.major_axis,'embryoOutline':['X','Y'],'pixCoords':coord})

self.embryoOutlineXR = xr.DataArray(outlineArray)

eggBB = self.results.ix[:,:,'eggBoxPoints'].values

# EGG BOX POINTS

eggArray = np.zeros((eggBB.shape[1],eggBB.shape[0],4,2))

for t in range(eggBB.shape[1]):

for f in range(eggBB.shape[0]):

if (type(eggBB[f,t]) != list):

eggArray[t,f,:,:] = eggBB[f,t]

else:

eggArray[t,f,:,:] = np.NaN

# Generate XArray

self.eggBBArray = xr.DataArray(eggArray,{'Items':self.results.items,'Frame':self.results.major_axis,'Dim':['X','Y'],'Point':['0','1','2','3']})

# Use a meshgrid to prevent the need for two loops to cycle over frames and timepoints.

frames, times = np.meshgrid(range(self.results.shape[1]),range(self.results.shape[0]))

times = times.flatten()

frames = frames.flatten()

self.tmp = []

# Initiate tmpTime

tmpTime = 0

for t in xrange(len(times)):

# Calculations not requiring looping over individual frames within this loop

# Loop over times and if a new one is reached, proceed.

if t is 0:

# Calculate movement stats

meanDisTmp, minDisTmp, maxDisTmp, totDisTmp = self.movementStats(times[t])

meanDistance.append(meanDisTmp)

minDistance.append(minDisTmp)

maxDistance.append(maxDisTmp)

totalDistance.append(totDisTmp)

# Rotated bounding box dimensions

mins = []

maxs = []

minDim, maxDim = self.calculateRotBBox(outlineArray,times[t], frames[t])

mins.append(minDim)

maxs.append(maxDim)

# Egg length and width

eggMin, eggMax = self.calculateEggRotBBox(eggArray,times[t], frames[t])

eggWidth.append(eggMin)

eggLength.append(eggMax)

if times[t] != tmpTime:

tmpTime = times[t]

# Calculate movement stats

meanDisTmp, minDisTmp, maxDisTmp, totDisTmp = self.movementStats(times[t])

meanDistance.append(meanDisTmp)

minDistance.append(minDisTmp)

maxDistance.append(maxDisTmp)

totalDistance.append(totDisTmp)

self.tmp.append(times[t])

# Rotated bounding box dimensions - if moved onto a new time point, save the previous time data and start afresh.

meanMinBB.append(np.nanmean(mins))

meanMaxBB.append(np.nanmean(maxs))

minMinBB.append(np.nanmin(mins))

maxMaxBB.append(np.nanmax(maxs))

mins = []

maxs = []

minDim, maxDim = self.calculateRotBBox(outlineArray,times[t], frames[t])

mins.append(minDim)

maxs.append(maxDim)

# Egg length and width - if moved onto a new time point, save the previous time data and start afresh.

meanEggWidth.append(np.nanmean(eggWidth))

meanEggLength.append(np.nanmean(eggLength))

eggWidth = []

eggLength = []

eggMin, eggMax = self.calculateEggRotBBox(eggArray,times[t], frames[t])

eggWidth.append(eggMin)

eggLength.append(eggMax)

# If at the end of the last time point

if t == len(times)-1:

# Add the last bounding box data to the BB stats.

minDim, maxDim = self.calculateRotBBox(outlineArray,times[t], frames[t])

mins.append(minDim)

maxs.append(maxDim)

meanMinBB.append(np.nanmean(mins))

meanMaxBB.append(np.nanmean(maxs))

minMinBB.append(np.nanmin(mins))

maxMaxBB.append(np.nanmax(maxs))

else:

# If not within a new timepoint continue calculating rotated bounding box dimensions and appending to list

minDim, maxDim = self.calculateRotBBox(outlineArray,times[t], frames[t])

mins.append(minDim)

maxs.append(maxDim)

eggMin, eggMax = self.calculateEggRotBBox(eggArray,times[t], frames[t])

eggWidth.append(eggMin)

eggLength.append(eggMax)

# Calculate growth

# Area

meanArGrowth, minArGrowth, maxArGrowth, meanArea, minArea, maxArea = self.calculateAreaGrowth()

meanArGrowthSlope, meanArGrowthIntercept, meanArGrowthR, meanArGrowthPval,meanArGrowthSE = meanArGrowth

minArGrowthSlope, minArGrowthIntercept, minArGrowthR, minArGrowthPval, minArGrowthSE = minArGrowth

maxArGrowthSlope, maxArGrowthIntercept, maxArGrowthR, maxArGrowthPval, maxArGrowthSE = maxArGrowth

# Length

meanMinLm, meanMaxLm, minMinLm, maxMaxLm = self.calculateLengthGrowth(meanMinBB,meanMaxBB,minMinBB,maxMaxBB)

meanMinLenGrowthSlope, meanMinLenGrowthIntercept, meanMinLenGrowthR, meanMinLenGrowthPval,meanMinLenGrowthSE = meanMinLm

meanMaxLenGrowthSlope, meanMaxLenGrowthIntercept, meanMaxLenGrowthR, meanMaxLenGrowthPval,meanMaxLenGrowthSE = meanMaxLm

minMinLenGrowthSlope, minMinLenGrowthIntercept, minMinLenGrowthR, minMinLenGrowthPval,minMinLenGrowthSE = minMinLm

maxMaxLenGrowthSlope, maxMaxLenGrowthIntercept, maxMaxLenGrowthR, maxMaxLenGrowthPval,maxMaxLenGrowthSE = maxMaxLm

# First find where is some data

ind = np.argmax(~np.isnan(self.freqOutputFour[:,4,4,1,0]))

bins = np.array([0,0.1,0.3,0.5,0.7,0.9,1.2,1.6,1.8,2.2,3.0,4.0,5.0,np.nanmax(self.freqOutputFour[:,:,:,0,:])])

# Extract data for the first time point for which there is osme

tmpData = self.freqOutputFour[ind,4,4,0,:]

# Now use the bins specified above to create a list of labels applicable to each data point/frequency to

# inidcate to which bin it belongs

binLabs = np.digitize(tmpData,bins)

# Create some lists for saving output

freqList_count = np.max(binLabs)

freqList = [[] for i in range(0, freqList_count)]

# Loop over the bins, calculate their energy and save to a list

for i in range(len(bins)):

# Create a filter to use to isolate the frequencies of relevance to the bin

tmp = (binLabs==np.arange(1,len(binLabs))[i])

if len(tmp) > 0:

freqList[i].append(np.nansum(self.freqOutputFour[:,:,:,1,tmp],axis=(1,2,3)))

# Debug

self.freqList = freqList

# Compile SummaryData output

timeSpecificData = pd.DataFrame(data = [meanArea, minArea, maxArea,

meanMinBB,meanMaxBB,minMinBB,maxMaxBB,

meanDistance,minDistance,maxDistance,

totalDistance,meanEggLength, meanEggWidth,

list(np.squeeze(freqList[0])),list(np.squeeze(freqList[1])),

list(np.squeeze(freqList[2])),list(np.squeeze(freqList[3])),

list(np.squeeze(freqList[4])),list(np.squeeze(freqList[5])),

list(np.squeeze(freqList[6])),list(np.squeeze(freqList[7])),

list(np.squeeze(freqList[8])),list(np.squeeze(freqList[9])),

list(np.squeeze(freqList[10])),list(np.squeeze(freqList[11])),

list(np.squeeze(freqList[12])),list(np.squeeze(freqList[13]))],

index = ['meanArea', 'minArea', 'maxArea',

'meanMinBB','meanMaxBB','minMinBB',

'maxMaxBB','meanDistance','minDistance',

'maxDistance','totalDistance',

'meanEggLength','meanEggWidth',

"FreqEnergy"+str(bins[0])+ ":"+ str(bins[1]),

"FreqEnergy"+str(bins[1])+ ":"+ str(bins[2]),

"FreqEnergy"+str(bins[2])+ ":"+ str(bins[3]),

"FreqEnergy"+str(bins[3])+ ":"+ str(bins[4]),

"FreqEnergy"+str(bins[4])+ ":"+ str(bins[5]),

"FreqEnergy"+str(bins[5])+ ":"+ str(bins[6]),

"FreqEnergy"+str(bins[6])+ ":"+ str(bins[7]),

"FreqEnergy"+str(bins[7])+ ":"+ str(bins[8]),

"FreqEnergy"+str(bins[8])+ ":"+ str(bins[9]),

"FreqEnergy"+str(bins[9])+ ":"+ str(bins[10]),

"FreqEnergy"+str(bins[10])+ ":"+ str(bins[11]),

"FreqEnergy"+str(bins[11])+ ":"+ str(bins[12]),

"FreqEnergy"+str(bins[12])+ ":"+ str(bins[13]),

"FreqEnergy"+str(bins[13])])

# Debug

self.timeSpData = timeSpecificData

# Combine two last freq outputs to get relevant sums..

timeSpecificData.ix[25,:] = timeSpecificData.ix[25,:] + timeSpecificData.ix[26,:]

timeSpecificData = timeSpecificData.drop(timeSpecificData.index[len(timeSpecificData.index)-1])

#timesp = timesp.drop('Freq5.0Hz-', axis=1)

# Add global data

globalData = {'MeanArGrowthRateSlope': meanArGrowthSlope, 'MeanArGrowthRateIntercept': meanArGrowthIntercept, 'MeanArGrowthR': meanArGrowthR, 'MeanArGrowthRatePval': meanArGrowthPval, 'MeanArGrowthSE': meanArGrowthSE,

'MinArGrowthRateSlope': minArGrowthSlope, 'MinArGrowthRateIntercept': minArGrowthIntercept, 'MinArGrowthR': minArGrowthR, 'MinArGrowthRatePval': minArGrowthPval, 'MinArGrowthSE': minArGrowthSE,

'MaxArGrowthRateSlope': maxArGrowthSlope, 'MaxArGrowthRateIntercept': maxArGrowthIntercept, 'MaxArGrowthR': maxArGrowthR, 'MaxArGrowthRatePval': maxArGrowthPval, 'MaxArGrowthSE': maxArGrowthSE,

'MeanMinLenGrowthRateSlope': meanMinLenGrowthSlope, 'MeanMinLenGrowthRateIntercept': meanMinLenGrowthIntercept, 'MeanMinLenGrowthR': meanMinLenGrowthR, 'MeanMinLenGrowthRatePval': meanMinLenGrowthPval, 'MeanMinLenGrowthSE': meanMinLenGrowthSE,

'MinMinLenGrowthRateSlope': minMinLenGrowthSlope, 'MinMinLenGrowthRateIntercept': minMinLenGrowthIntercept, 'MinMinLenGrowthR': minMinLenGrowthR, 'MinMinLenGrowthRatePval': minMinLenGrowthPval, 'MinMinLenGrowthSE': minMinLenGrowthSE,

'MaxMaxLenGrowthRateSlope': maxMaxLenGrowthSlope, 'MaxMaxLenGrowthRateIntercept': maxMaxLenGrowthIntercept, 'MaxMaxLenGrowthR': maxMaxLenGrowthR, 'MaxMaxLenGrowthRatePval': maxMaxLenGrowthPval, 'MaxMaxLenGrowthSE': maxMaxLenGrowthSE}

return timeSpecificData, globalData

#%%

#==============================================================================

# Quantify energy within different frequency bands in parallel

# (called from calculatePhenomeMeasures) generate freq data from blockwise data.

#==============================================================================

def parFreqAnalysis(self,t):

# Get necessary info..

frameRate = self.results.shape[1]/((np.float(self.results.ix[0,self.results.shape[1]-1,'elapsedTime'])-np.float(self.results.ix[0,0,'elapsedTime']))/1000)

sampFreqs, powerSpect = signal.welch(self.blockWiseArrayOneXR.values[t,:],frameRate, scaling='spectrum', nfft=self.blockWiseArrayOneXR.shape[1])

# Arrays for output

ef1 = np.zeros((2,len(powerSpect)))

ef2 = np.zeros((2,2,2,len(powerSpect)))

ef3 = np.zeros((4,4,2,len(powerSpect)))

ef4 = np.zeros((8,8,2,len(powerSpect)))

ef5 = np.zeros((16,16,2,len(powerSpect)))

if not np.isnan(self.blockWiseArrayOneXR.values[t,0]):

# Create and run meshgrids to prevent need for nested loops..

X2,Y2 = np.meshgrid(range(2),range(2))

X2 = X2.flatten()

Y2 = Y2.flatten()

X3,Y3 = np.meshgrid(range(4),range(4))

X3 = X3.flatten()

Y3 = Y3.flatten()

X4,Y4 = np.meshgrid(range(8),range(8))

X4 = X4.flatten()

Y4 = Y4.flatten()

X5,Y5 = np.meshgrid(range(16),range(16))

X5 = X5.flatten()

Y5 = Y5.flatten()

# Run analysis

# 1x1

ef1[0,:], ef1[1,:] = signal.welch(self.blockWiseArrayOneXR.values[t,:],frameRate, scaling='spectrum', nfft=self.results.shape[1])

# 2x2

for b in xrange(4):

tmp = self.blockWiseArrayTwoXR.values

ef2[X2[b],Y2[b],0,:], ef2[X2[b],Y2[b],1,:] = signal.welch(tmp[t,:,X2[b],Y2[b]],frameRate, scaling='spectrum', nfft=self.results.shape[1])

# 4x4

for b in xrange(16):

tmp = self.blockWiseArrayThreeXR.values

ef3[X3[b],Y3[b],0,:], ef3[X3[b],Y3[b],1,:] = signal.welch(tmp[t,:,X3[b],Y3[b]],frameRate, scaling='spectrum', nfft=self.results.shape[1])

# 8x8

for b in xrange(64):

tmp = self.blockWiseArrayFourXR.values

ef4[X4[b],Y4[b],0,:], ef4[X4[b],Y4[b],1,:] = signal.welch(tmp[t,:,X4[b],Y4[b]],frameRate, scaling='spectrum', nfft=self.results.shape[1])

# 16x16

for b in xrange(256):

tmp = self.blockWiseArrayFiveXR.values

ef5[X5[b],Y5[b],0,:], ef5[X5[b],Y5[b],1,:] = signal.welch(tmp[t,:,X5[b],Y5[b]],frameRate, scaling='spectrum', nfft=self.results.shape[1])

else:

# If no blockwise signal assign NaN

ef1[:,:] = np.NaN

ef2[:,:,:,:] = np.NaN

ef3[:,:,:,:] = np.NaN

ef4[:,:,:,:] = np.NaN

ef5[:,:,:,:] = np.NaN

# Return numpy arrays in a dict for unpacking.

return {'F1':ef1,'F2':ef2,'F3':ef3,'F4':ef4,'F5':ef5}

#==============================================================================

# LENGTH GROWTH - Calculate mean, min and max slope stats (slope, intercept and pvalue)

#==============================================================================

def calculateLengthGrowth(self,meanMinBB,meanMaxBB,minMinBB,maxMaxBB):

# Mask to remove nans Note: A rolling window is used here in developing the mask

# (and below) to remove outlying (temporally) data.

mask = ~pd.Series(np.array(meanMinBB)).rolling(window=3).mean().isnull()

if np.sum(~pd.Series(np.array(meanMinBB)).rolling(window=3).mean().isnull()) == 0:

return float('nan'), float('nan'), float('nan'), float('nan')

# Date time is formatted appropriately

filteredDateTimes = self.results.ix[:,0,'dateTime'].values[mask]

DHMS = pd.to_datetime(filteredDateTimes)-pd.to_datetime(filteredDateTimes[0])

fromExptStartMINS = np.array((DHMS/np.timedelta64(1,'m')).astype(np.float))

# Generate growth data (using mask to remove nans, otherwise output for an embryo is NaN)

meanMinLenLm = scipy.stats.linregress(x=fromExptStartMINS, y = np.array(meanMinBB)[mask]*self.scale)

meanMaxLenLm = scipy.stats.linregress(x=fromExptStartMINS, y = np.array(meanMaxBB)[mask]*self.scale)

minMinLenLm = scipy.stats.linregress(x=fromExptStartMINS, y = np.array(minMinBB)[mask]*self.scale)

maxMaxLenLm = scipy.stats.linregress(x=fromExptStartMINS, y = np.array(maxMaxBB)[mask]*self.scale)

# Debug

# plt.plot(fromExptStartMINS, np.array(self.meanMinBB)[mask]*self.scale)

# plt.plot(fromExptStartMINS, meanMinLenLm.intercept + meanMinLenLm.slope*fromExptStartMINS, 'r', label='fitted line', color = 'black')

return meanMinLenLm, meanMaxLenLm, minMinLenLm, maxMaxLenLm

#==============================================================================

# AREA GROWTH - Calculate mean, min and max slope stats (slope, intercept and pvalue)

#==============================================================================

def calculateAreaGrowth(self):

if self.mode is not 'xarray':

# If too much missing data do not proceed

if np.nansum(np.nanmean(self.results.ix[:,:,'area'], axis=0)) ==0:

#np.sum(~pd.Series(np.nanmean(self.results.ix[:,:,'area'],axis=0)).rolling(window =3).mean().isnull()) ==0:

return (float('nan'), float('nan'), float('nan'), float('nan'), float('nan')), (float('nan'), float('nan'),float('nan'), float('nan'), float('nan')), (float('nan'), float('nan'), float('nan'), float('nan'), float('nan')), [float('nan')]*self.results.shape[0], [float('nan')]*self.results.shape[0], [float('nan')]*self.results.shape[0]

else:

# Date time is formatted appropriately

filteredDateTimes = self.results.ix[:,0,'dateTime'].values

DHMS = pd.to_datetime(filteredDateTimes)-pd.to_datetime(filteredDateTimes[0])

fromExptStartMINS = np.array((DHMS/np.timedelta64(1,'h')).astype(np.float))

# Raw data - note conversion with metadata scale

meanArea = np.nanmean(self.results.ix[:,:,'area'].values.astype(np.float), axis = 0)*(self.scale*self.scale)

minArea = np.nanmin(self.results.ix[:,:,'area'].values.astype(np.float), axis = 0)*(self.scale*self.scale)

maxArea = np.nanmax(self.results.ix[:,:,'area'].values.astype(np.float), axis = 0)*(self.scale*self.scale)

# Generate growth data, including a scale conversion

noDataMask = ~np.isnan(meanArea)

meanLm = scipy.stats.linregress(x=fromExptStartMINS[noDataMask], y = np.log(meanArea)[noDataMask])

minLm = scipy.stats.linregress(x=fromExptStartMINS[noDataMask], y = np.log(minArea)[noDataMask])

maxLm = scipy.stats.linregress(x=fromExptStartMINS[noDataMask], y = np.log(maxArea)[noDataMask])

# Debug

# Plot data

# plt.figure()

# plt.plot(fromExptStartMINS, np.log(np.array(meanArea).astype(float)), color = 'black')

# plt.plot(fromExptStartMINS,np.log(np.array(minArea).astype(float)), color = 'blue')

# plt.plot(fromExptStartMINS,np.log(np.array(maxArea).astype(float)), color = 'orange')

# Add model to plot

# plt.plot(fromExptStartMINS, meanLm.intercept + meanLm.slope*fromExptStartMINS, 'r', label='fitted line', color = 'black')

# plt.plot(fromExptStartMINS, minLm.intercept + minLm.slope*fromExptStartMINS, 'r', label='fitted line', color = 'blue')

# plt.plot(fromExptStartMINS, maxLm.intercept + maxLm.slope*fromExptStartMINS, 'r', label='fitted line', color = 'orange')

# Output growth model stats and raw data

if self.mode is 'xarray':

if np.nansum(np.nanmean(self.results['SizePos'].loc[:,:,'area'], axis=0)) ==0:

#np.sum(~pd.Series(np.nanmean(self.results.ix[:,:,'area'],axis=0)).rolling(window =3).mean().isnull()) ==0:

return (float('nan'), float('nan'), float('nan'), float('nan'), float('nan')), (float('nan'), float('nan'),float('nan'), float('nan'), float('nan')), (float('nan'), float('nan'), float('nan'), float('nan'), float('nan')), [float('nan')]*self.results.shape[0], [float('nan')]*self.results.shape[0], [float('nan')]*self.results.shape[0]

else:

#print 'here'

### Incorporate a mask to remove very high solidities, likely the entire egg - i.e. issues with segmentation?

solidityMask = np.nanmean(self.results['SizePos'].loc[:,:,'solidity'].values,axis=1) < 0.99

meanArea = np.nanmean(self.results['SizePos'].loc[:,:,'area'].values, axis=1)

meanArea[~solidityMask] = np.NaN

meanArea = meanArea*(self.scale*self.scale)

minArea = np.nanmean(self.results['SizePos'].loc[:,:,'area'].values, axis=1)

minArea[~solidityMask] = np.NaN

minArea = minArea*(self.scale*self.scale)

maxArea = np.nanmax(self.results['SizePos'].loc[:,:,'area'].values, axis=1)

maxArea[~solidityMask] = np.NaN

maxArea = maxArea*(self.scale*self.scale)

# Date time is formatted appropriately

filteredDateTimes = self.results['dateTime'].values

DHMS = pd.to_datetime(filteredDateTimes)-pd.to_datetime(filteredDateTimes[0])

fromExptStartMINS = np.array((DHMS/np.timedelta64(1,'h')).astype(np.float))

# Generate growth data, including a scale conversion

noDataMask = ~np.isnan(meanArea)

meanLm = scipy.stats.linregress(x=fromExptStartMINS[noDataMask], y = np.log(meanArea)[noDataMask])

minLm = scipy.stats.linregress(x=fromExptStartMINS[noDataMask], y = np.log(minArea)[noDataMask])

maxLm = scipy.stats.linregress(x=fromExptStartMINS[noDataMask], y = np.log(maxArea)[noDataMask])

# Debug

# Plot data

plt.figure()

plt.plot(fromExptStartMINS, np.log(np.array(meanArea).astype(float)), color = 'black')

plt.plot(fromExptStartMINS,np.log(np.array(minArea).astype(float)), color = 'blue')

plt.plot(fromExptStartMINS,np.log(np.array(maxArea).astype(float)), color = 'orange')

# Add model to plot

plt.plot(fromExptStartMINS, meanLm.intercept + meanLm.slope*fromExptStartMINS, 'r', label='fitted line', color = 'black')

plt.plot(fromExptStartMINS, minLm.intercept + minLm.slope*fromExptStartMINS, 'r', label='fitted line', color = 'blue')

plt.plot(fromExptStartMINS, maxLm.intercept + maxLm.slope*fromExptStartMINS, 'r', label='fitted line', color = 'orange')

plt.title(str(self.embryo))

# Output growth model stats and raw data

return (meanLm.slope, meanLm.intercept, meanLm.rvalue, meanLm.pvalue, meanLm.stderr), (minLm.slope, minLm.intercept, minLm.rvalue, minLm.pvalue, minLm.stderr), (maxLm.slope, maxLm.intercept, maxLm.rvalue, maxLm.pvalue, maxLm.stderr), meanArea, minArea, maxArea

#==============================================================================

# Determine rotated bounding box of embryo (to get min and max dimension)

#==============================================================================

def calculateRotBBox(self,coords,timesT,framesT):

if (np.mean(coords[timesT,framesT,0,:]) > 0):

# Get rotated bounding box

(_,_),(w,h),_ = cv2.minAreaRect(np.array((coords[timesT,framesT,0,coords[timesT,framesT,0,:]>0],coords[timesT,framesT,1,coords[timesT,framesT,1,:]>0])).astype(np.int).T)

minDim = np.min((w,h))

maxDim = np.max((w,h))

else:

minDim = np.NaN

maxDim = np.NaN

return minDim, maxDim

#==============================================================================

# Determine rotated bounding box of egg (to get min and max dimension)

#==============================================================================

def calculateEggRotBBox(self,eggBB,timesT,framesT):

if (np.mean(eggBB[timesT,framesT,0,:]) > 0):

# Get rotated bounding box

(_,_),(w,h),_ = cv2.minAreaRect(np.array(eggBB[timesT,framesT,:,:].astype(np.int)))

minDim = np.min((w,h))

maxDim = np.max((w,h))

else:

minDim = np.NaN

maxDim = np.NaN

return minDim, maxDim

#==============================================================================

# MOVEMENT

#==============================================================================

def movementStats(self,t):

# If not more than 10 data points missing for the time point then proceed with movement stat calculation.

movementDataX = self.results.loc[:,:,'centroidX'].values.astype(np.float)

movementDataY = self.results.loc[:,:,'centroidY'].values.astype(np.float)

if (np.sum(np.isnan(movementDataX[:,t])) <10):

distances = np.linalg.norm(np.array((movementDataX[:,t],movementDataY[:,t])).T[:-1] - np.array((movementDataX[:,t],movementDataY[:,t])).T[1:], axis=1)

meanDist = np.nanmean((distances*self.scale))

minDist = np.nanmin((distances*self.scale))

maxDist = np.nanmax((distances*self.scale))

totalDist = np.nansum((distances*self.scale))

else:

# If too much missing data then assign NaN

meanDist = np.NaN

minDist = np.NaN

maxDist = np.NaN

totalDist = np.NaN

return meanDist, minDist, maxDist, totalDist

**dataAnalysis.py**

import cv2

import numpy as np

import matplotlib.pyplot as plt

import numpy.ma as ma

import pyqtgraph as pg

import pandas as pd

import glob

import os

import re

from skimage.segmentation import clear_border

from skimage.morphology import disk

from PyQt5.Qt import *

import sys

#from imageAnalysis import imageAnalysis

import eggUI

#import viewOutput

import time

import pathos

import json

#import tables

import scipy

import scipy.fftpack

import pylab

from scipy import pi

import scipy.signal as signal

import peakutils

import xarray as xr

import math

from rpy2.robjects.packages import importr

import rpy2.robjects as robjects

from matplotlib.backends.backend_pdf import PdfPages

import statsmodels.api as sm

import statsmodels.formula.api as smf

from sklearn import datasets, linear_model

from sklearn.metrics import mean_squared_error, r2_score

class dataAnalysis(object):

#==============================================================================

# Produce summary reports for each embryo and save to savePath.

# Note: savePath must exist i.e. create the folder beforehand..

#==============================================================================

def generateSummaryReports(self,savePath):

for e in range(len(self.embryoLabels)):

self.embryo = self.embryoLabels[e]

self.loadXRResults()

if self.results is not 'NoData':

data = self.results['TimeSpecificSummaryData'].to_pandas()

f, ax = plt.subplots(4, sharex=True)

ax[0].set_yscale('log')

# Size

for i in range(13,24):

ax[0].fill_between(np.arange(0,len(data.ix[:,i].values)),data.ix[:,i].values, alpha = 0.3)

ax[0].set_title('Enery at different frequencies')

ax[1].plot(data.ix[:,0].values)

ax[1].fill_between(np.arange(0,len(data.ix[:,1].values)),data.ix[:,1].values,data.ix[:,2].values, alpha = 0.3)

ax[1].set_title('Min:Max and mean area')

ax[2].plot(data.ix[:,3].values)

ax[2].fill_between(np.arange(0,len(data.ix[:,1].values)),data.ix[:,4].values,data.ix[:,5].values, alpha = 0.3)

ax[2].set_title('Min:Max and mean BB')

ax[3].plot(data.ix[:,7].values)

ax[3].set_yscale('log')

ax[3].fill_between(np.arange(0,len(data.ix[:,1].values)),data.ix[:,8].values,data.ix[:,9].values, alpha = 0.3)

ax[3].set_title('Min:Max and mean distance')

plt.tight_layout(pad=0.4, w_pad=0.5, h_pad=1.0)

pdf_pages = PdfPages(savePath + self.embryoLabels[e] + '_phenomeSummary.pdf')

pdf_pages.savefig(f)

pdf_pages.close()

plt.plot()

plt.close()

#==============================================================================

# Measure heart rate for all embryos

#==============================================================================

def measureHeartRateForAllEmbryos(self,savePath,filtMin=False,filtMax=False,histCount=False,segRegfiltXVal=False,segRegfiltYVal=False,minXVal=False,peakThresh=False):

if self.species == 'rbalthica':

# If user has not specified these 'tweakable paramters' in the HR ID

# and model fitting use these values

if filtMin == False:

filtMin = 1

if filtMax == False:

filtMax = 2

if histCount == False:

histCount = 6

if segRegfiltXVal == False:

segRegfiltXVal = 5

if segRegfiltYVal == False:

segRegfiltYVal = 6

if minXVal == False:

minXVal = 60

if peakThresh == False:

peakThresh = 0.4

for e in range(len(self.embryoLabels)):

self.embryo = self.embryoLabels[e]

self.loadXRResults()

self.measureHeartRate_radix(savePath,filtMin,filtMax,histCount,segRegfiltXVal,segRegfiltYVal,minXVal,peakThresh)

np.save(savePath + '/hrdata.npy', self.heartRate_data)

if self.species == 'ogammarellus':

# If user has not specified these 'tweakable paramters' in the HR ID

# and model fitting use these values

if filtMin == False:

filtMin = 0.5

if filtMax == False:

filtMax = 4

if histCount == False:

histCount = 6

if minXVal == False:

minXVal = False

if peakThresh == False:

peakThresh = 0.2

print str(savePath) + '_' + str(filtMin) + '_' +str(filtMax) + '_' + str(histCount) + '_' + str(peakThresh)

for e in range(len(self.embryoLabels)):

self.embryo = self.embryoLabels[e]

self.loadXRResults()

self.measureHeartRate_orchestia(savePath,filtMin,filtMax,histCount,peakThresh)

np.save(savePath + '/hrdata.npy', self.heartRate_data)

#==============================================================================

# Modify heart rate modelling for specific embryo

#==============================================================================

def measureHeartRateForSpecificEmbryos(self,embryo,savePath,filtMin=False,filtMax=False,histCount=False,segRegfiltXVal=False,segRegfiltYVal=False,minXVal=False,peakThresh=False):

if self.species == 'rbalthica':

# If user has not specified these 'tweakable paramters' in the HR ID

# and model fitting use these values

if filtMin == False:

filtMin = 1

if filtMax == False:

filtMax = 2

if histCount == False:

histCount = 6

if segRegfiltXVal == False:

segRegfiltXVal = 5

if segRegfiltYVal == False:

segRegfiltYVal = 6

if minXVal == False:

minXVal = 60

if peakThresh == False:

peakThresh = 0.4

if isinstance(embryo, str):

self.heartRate_data = np.load(savePath + '/hrdata.npy')

self.heartRate_data = self.heartRate_data[()]

self.embryo = embryo

self.loadXRResults()

self.measureHeartRate_radix(savePath,filtMin,filtMax,histCount,segRegfiltXVal,segRegfiltYVal,minXVal,peakThresh)

np.save(savePath + '/hrdata.npy', self.heartRate_data)

if isinstance(embryo, list):

self.heartRate_data = np.load(savePath + '/hrdata.npy')

self.heartRate_data = self.heartRate_data[()]

for e in range(len(embryo)):

self.embryo = embryo[e]

self.loadXRResults()

self.measureHeartRate_radix(savePath,filtMin,filtMax,histCount,segRegfiltXVal,segRegfiltYVal,minXVal,peakThresh)

np.save(savePath + '/hrdata.npy', self.heartRate_data)

elif self.species == 'ogammarellus':

if filtMin == None:

filtMin = 0.5

if filtMax == None:

filtMax = 4

if histCount == None:

histCount = 6

if minXVal == None:

minXVal = False

if peakThresh == None:

peakThresh = 0.2

if isinstance(embryo, str):

self.heartRate_data = np.load(savePath + '/hrdata.npy')

self.heartRate_data = self.heartRate_data[()]

self.embryo = embryo

self.loadXRResults()

self.measureHearRate_orchestia(self,savePath,filtMin,filtMax,histCount,peakThresh)

np.save(savePath + '/hrdata.npy', self.heartRate_data)

if isinstance(embryo, list):

self.heartRate_data = np.load(savePath + '/hrdata.npy')

self.heartRate_data = self.heartRate_data[()]

for e in range(len(embryo)):

self.embryo = embryo[e]

self.loadXRResults()

self.measureHearRate_orchestia(self,savePath,filtMin,filtMax,histCount,peakThresh)

np.save(savePath + '/hrdata.npy', self.heartRate_data)

#==============================================================================

# Load heart rate data

#==============================================================================

def loadHeartRateData(self,savePath):

self.heartRate_data = np.load(savePath + '/hrdata.npy')

self.heartRate_data = self.heartRate_data[()]

#==============================================================================

# Umbrella function for measuring heart rate - Radix balthica

#==============================================================================

def measureHeartRate_orchestia(self,savePath,filtMin,filtMax,histCount,peakThresh):

if not hasattr(self,'heartRate_data'):

self.heartRate_data = dict()

if self.results is 'NoData':

print 'No data for ' + str(self.embryo)

freqres = self.results['FreqOutput_8x8'].values

gs, fs = np.meshgrid(range(8),range(8))

gs = gs.flatten()

fs = fs.flatten()

HRs=[]

# Loop over time points

for t in range(self.results['FreqOutput_8x8'].values.shape[0]):

HRFreqs=[]

# Loop over blockwise signals

for b in range(len(gs)):

powerSpect = freqres[t,fs[b],gs[b],1,:]

if (powerSpect.max() == 0.0) or np.isnan(powerSpect[0]):

HRFreqs.append(np.NaN)

else:

sig = pd.rolling_mean(self.results['BlockWise_8x8'].loc[t,:,fs[b],gs[b]].values, window=3)

sig = pd.DataFrame(sig).interpolate(limit_direction = 'both').values.ravel()

# Interpolate to fill missing data

frameRate = sig.shape[0]/((self.results['SizePos'].to_pandas().ix[t,sig.shape[0]-1,'elapsedTime'] - self.results['SizePos'].to_pandas().ix[t,0,'elapsedTime'])/1000)

sampFreqs, powerSpect = signal.welch(sig,frameRate, scaling='spectrum')

baselineRemoved = np.log(powerSpect) - peakutils.baseline(np.log(powerSpect)- min(np.log(powerSpect)))

# Now ID peaks.anan

indexes = peakutils.indexes(baselineRemoved[0:200], peakThresh, min_dist=0)

outPeakFreqs = sampFreqs[indexes]

inds = np.where(((outPeakFreqs < filtMax) & (outPeakFreqs > filtMin)))

filtFreqs = outPeakFreqs[inds]

filtPower = baselineRemoved[inds]

# If more than one freq peak identified within the range 0.5-3 Hz

if inds[0].shape[0] > 1:

#print str(b)

# Identify freq with most power

maxInd = np.argmax(filtPower)

HRFreq = filtFreqs[maxInd]

HRFreqs.append(HRFreq)

elif inds[0].shape[0] == 1:

HRFreq = outPeakFreqs[inds[0][0]]

HRFreqs.append(HRFreq)

else:

HRFreqs.append(np.NaN)

else:

HRFreqs.append(np.NaN)

HRFreqs = np.array(HRFreqs)

# Remove NaNs

count, freqs = np.histogram(HRFreqs[np.invert(np.isnan(HRFreqs))],bins=50)

# Identify whether a sufficiently dominant frequency to reliably ID HR.

inds = np.where(count > histCount)

#plt.hist(HRFreqs[~np.isnan(HRFreqs)],20)

if len(inds[0]) is not 0:

# Get max ind

maxInds = inds[-1]

HR = np.mean(freqs[maxInds])

print HR

HRs.append(HR)

else:

HRs.append(np.NaN)

# Attempt to fit a linear model to data from 24 h post HR detection..

try:

# Use for fitting model

reducedHRs = HRs[23:]

# Fit a linear model to Orchestia heart function ontogeny

# Format data

pdData = np.array([np.arange(len(reducedHRs))[~np.isnan(reducedHRs)], np.array(reducedHRs)[~np.isnan(reducedHRs)]]).T

df = pd.DataFrame(pdData,columns=['Ind','HR'])

mod = smf.ols(formula='HR ~ Ind', data=df)

res = mod.fit()

print(res.summary())

# If any data points have residuals > 0.5, remove these.

if np.any(np.sqrt(res.resid*res.resid) > 0.5):

df = df.drop(df.ix[np.sqrt(res.resid*res.resid) > 0.5,:].index)

mod = smf.ols(formula='HR ~ Ind', data=df)

res = mod.fit()

# Get predicted HR values

# predictedHRs = res.predict(np.arange(24))

predictedHRs = self.res.predict(pd.DataFrame(data = np.arange(24), columns = ['Ind']))

# Get intercept and gradient

plt.figure()

p = mod.fit().params

print 'Intercept: ' + str(p[0]) + '; Slope: ' + str(p[1])

plt.plot(df.ix[:,'Ind'],df.ix[:,'HR'],'o')

plt.plot(df.ix[:,'Ind'], p[0] + p[1] * df.ix[:,'Ind'],color='black')

plt.ylim(0,df.ix[:,'HR'].max()*1.2)

zip(np.arange(0, len(self.results['dateTime'].values[23:])),self.results['dateTime'].values,self.results['Metadata'].to_pandas().ix[23:,0,'currentFolder'],HRs[23:],predictedHRs)

self.heartRate_data[str(self.embryo)] = {'Embryo': self.embryo,'PredHR':predictedHRs, 'Intercept': str(p[0]), 'Slope':str(p[1]),

'modPars_filtMin':filtMin,'modPars_filtMax':filtMax,'modPars_histCount':histCount,'modPars_peakThresh':peakThresh}

except:

print '*** Heart rate not succesfully modelled for ' + str(self.embryo) + ' ***'

#==============================================================================

# Umbrella function for measuring heart rate - Radix balthica

#==============================================================================

def measureHeartRate_radix(self,savePath,filtMin=1,filtMax=2,histCount=6,segRegfiltXVal=5,segRegfiltYVal=6,minXVal=False,peakThresh=0.4):

""" Attempt to extract and model heart rate from 8x8 blockwise frequency data.

Note that some user guidance is recommended and if the heart rate cannot be succesfully

modelled try changing some of these optional arguments:

# Provide a folder for saving output plots and statistics.

# The minimum acceptable HR (Hz)

filtMin=1

# The maximum acceptable HR (Hz)

filtMax=2

# The number of signals in which a frequency must occur as being dominant to be considered.

histCount=6

# The extent to which outlying values are filtered in the X dimension prior to fitting a segmented regression.

segRegfiltXVal=4

# The extent to which outlying values are filtered in the Y dimension prior to fitting a segmented regression.

segRegfiltYVal=6

# The threshold for identifying peaks in the frequency output

peakThresh=0.4

"""

#if 'heartRate_data' not in locals():

if not hasattr(self,'heartRate_data'):

self.heartRate_data = dict()

if self.results is 'NoData':

print 'No data for ' + str(self.embryo)

else:

self.freqRes = self.results['FreqOutput_8x8'].values

hrvals = []

gs, fs = np.meshgrid(range(8),range(8))

gs = gs.flatten()

fs = fs.flatten()

for i in range(self.freqRes.shape[0]):

hrvals.append(self.modelHR_radix(gs,fs,i,filtMin,filtMax,histCount,minXVal,peakThresh))

self.test = hrvals

#try:

self.predHR, self.predBP, self.BP, self.S1,self.S2,self.startTime,self.endTime,self.firstHR,self.lastHR,self.HRData,self.outputSummary = self.segReg_radix(hrvals,segRegfiltXVal,segRegfiltYVal)

print 'making it to here'

# Show fit

fig, ax = plt.subplots(1,1)

ax.plot(self.predHR)

ax.plot(self.HRData,'o')

#ax.plot(sortedTimes, sortedPredicted)

#ax.plot(times, np.array(HRData)[~np.isnan(HRData)], 'o')

plt.title(str(self.embryo))

plt.show()

plt.xlabel('Time point')

plt.ylabel('Frequency (Hz)')

plt.ylim(0,(np.nanmax(np.array((self.predHR,self.HRData)))*1.2))

fig.subplots_adjust(bottom=0.3)

fig.text(.1,.1,str(self.outputSummary[0] + '\n' + self.outputSummary[1] + '\n' + self.outputSummary[2]))

fig.canvas.manager.window.raise_()

pdf_pages = PdfPages(savePath + self.embryo + '_heartModelSummary.pdf')

pdf_pages.savefig(fig)

pdf_pages.close()

plt.plot()

print 'plotting is ok'

#time.sleep(0.2)

# Add data to a dict - new key for each embryo

self.heartRate_data[str(self.embryo)] = {'Embryo': self.embryo,'PredHR':self.predHR,

'PredBP':self.predBP, 'BP': self.BP, 'S1': self.S1,'S2':self.S2,'startTime':self.startTime,

'endTime':self.endTime,'firstHR':self.firstHR,'lastHR':self.lastHR,'HRData':self.HRData,

'modPars_filtMin':filtMin,'modPars_filtMax':filtMax,'modPars_histCount':histCount,'modPars_segRegfiltXVal':segRegfiltXVal,

'modPars_segRegfiltYVal':segRegfiltYVal,'modPars_minXVal':minXVal,'modPars_peakThresh':peakThresh}

#except:

# print '*** Heart rate not succesfully modelled for ' + str(self.embryo) + ' ***'

#==============================================================================

# Model HR for Radix balthica

#==============================================================================

# Extract peaks from the freq output that may be HR.

def modelHR_radix(self,gs,fs, t,filtMin, filtMax, histCount, minXVal,peakThresh):

""" Called from measureHeartRate_radix().

"""

HRFreqs=[]

# If no minVal (time point) provided, or the t value is above the minimum value

# attempt to ID the heart rate.

if type(minXVal) is not bool and minXVal<= t or not minXVal:

if not math.isnan(self.freqRes[t,0,0,1,0]):

for b in range(len(gs)):

powerSpect = self.freqRes[t,fs[b],gs[b],1,:]

if (powerSpect.max() == 0.0):

HRFreqs.append(np.NaN)

else:

sampFreqs = self.freqRes[t,fs[b],gs[b],0,:]

try:

baselineRemoved = np.log(powerSpect) - peakutils.baseline(np.log(powerSpect)- min(np.log(powerSpect)))

# Now ID peaks.anan

indexes = peakutils.indexes(baselineRemoved, peakThresh, min_dist=1)

outPeakFreqs = sampFreqs[indexes]

# And get magnitudes (may be useful for downstream filtering).

outMagnitudes = baselineRemoved[indexes]

# Identify index(or indices)

# Gam

#inds = np.where(((outPeakFreqs < 5) & (outPeakFreqs > 0.5)))

# Rad

#inds = np.where(((outPeakFreqs < 2) & (outPeakFreqs > 1)))

inds = np.where(((outPeakFreqs < filtMax) & (outPeakFreqs > filtMin)))

filtFreqs = outPeakFreqs[inds]

filtPower = baselineRemoved[inds]

# If more than one freq peak identified within the range 0.5-3 Hz

if inds[0].shape[0] > 1:

#print str(b)

# Identify freq with most power

maxInd = np.argmax(filtPower)

HRFreq = filtFreqs[maxInd]

HRFreqs.append(HRFreq)

elif inds[0].shape[0] == 1:

HRFreq = outPeakFreqs[inds[0][0]]

HRFreqs.append(HRFreq)

else:

HRFreqs.append(np.NaN)

except:

HRFreqs.append(np.NaN)

else:

HRFreqs.append(np.NaN)

HRFreqs = np.array(HRFreqs)

# Remove NaNs

count, freqs = np.histogram(HRFreqs[np.invert(np.isnan(HRFreqs))],bins=50)

# Identify whether a sufficiently dominant frequency to reliably ID HR.

inds = np.where(count > histCount)

if len(inds[0]) is not 0:

# Get max ind

maxInds = inds[-1]

HR = np.mean(freqs[maxInds])

else:

HR = np.NaN

else:

HR = np.NaN

else:

# If current time iteration is below the min value (time) assign NaN.

HR = np.NaN

return HR

#==============================================================================

# HR - fits a segmented regression using R.

# Used to model HR for R. balthica

#==============================================================================

def segReg_radix(self,HRData,filtXVal,filtYVal):

# Fit segmented regressionpN in R to the HR data. Note various filtering done here..

base = importr('base')

r = robjects.r

seg = importr('segmented')

# Filter data to remove lone x axis

# ID where values exist

filt = ~np.isnan(HRData)

# Use histogram to ID outlier

count, bins = np.histogram(np.array(range(0,len(HRData)))[filt])

# Filter to remove outlying x values

filtInd = bins[:-1][(count > 0) & (count < filtXVal)]

filtInds = np.nonzero([(count > 0) & (count < filtXVal)])[1]

# If any time points to filter

HRData = np.array(HRData)

if filtInd.shape[0] is not 0:

#HRData[filtInd.astype(np.int)] = np.NaN

# Add filter to remove any values within particular bin requiring filtering..

for i in range(len(filtInds)):

for t in range(len(HRData)):

if (t >= bins[filtInds][i]) & (t <= bins[filtInds[i]+1]):

# Debug

# print 'X filtered'

HRData[t] = np.NaN

# Filter to remove outlying y values

Yax, bins = np.histogram(HRData[~np.isnan(HRData)])

filtInd = Yax < filtYVal

filtInds = np.nonzero([Yax < filtYVal])[1]

if filtInds.shape[0] is not 0:

#Yax[1][1:][Yax[0] < 2]

for i in range(len(filtInds)):

for t in range(len(HRData)):

if (HRData[t] >= bins[filtInds][i]) & (HRData[t] <= bins[filtInds[i]+1]):

#print 'success'

HRData[t] = np.NaN

# Format/convert data

HR = robjects.FloatVector(HRData)

Time = robjects.FloatVector(range(0,len(HRData)))

robjects.globalenv["HR"] = HR

robjects.globalenv["Time"] = Time

# Run linear model

lmHR = r.lm("HR ~ Time")

#print(base.summary(lmHR))

# Model for seg.z

formula = robjects.Formula("~Time")

# Also use mean of data containing values to get starting psi

startingPSI = np.int(np.median(np.arange(0,len(HRData))[~np.isnan(HRData)]))

# Run segmented model and print results

segModHR = seg.segmented(lmHR, seg_Z=formula, psi=startingPSI, model=True)

#print(seg.summary_segmented(segModHR, short = True))

# print(seg.print.segmented(segModHR))

# Plotting segmented in Python

resultsDict = dict(zip(segModHR.names, list(segModHR)))

breakPoint = resultsDict['psi'][1]

# Get fitted values

predicted = np.array(seg.predict_segmented(segModHR))

times = np.arange(0,len(HRData))[~np.isnan(HRData)]

#plt.plot(times, np.array(HRData)[~np.isnan(HRData)], 'o')

# Get slopes

slopes = seg.slope(segModHR)

slopes = np.array(slopes[0])

slopeOne = slopes[0,0]

slopeTwo = slopes[1,0]

#print(seg.summary_segmented(segModHR))

self.out = seg.summary_segmented(segModHR)

self.tmp = segModHR.items

# Get fitted values for all times (predicted values above skip missing data)

# Create an R dataframe with x-values to predict from

d = {'Time': robjects.FloatVector(np.arange(times.min(),times.max()))}

dataf = robjects.DataFrame(d)

# Predict values - all times (although when plotting in Matplotlib the breakpoint will appear slightly flattened)

predictedVals = r.predict(segModHR, newdata = dataf)

#plt.plot(np.arange(times.min(),times.max()),np.array(predictedVals))

# Get fitted value for breakpoint - this must be used in matplotlib plotting for break to appear appropriately

d = {'Time': robjects.FloatVector([breakPoint])}

breakpointTime = robjects.DataFrame(d)

pyBreakpointTime = np.array(breakpointTime)[0][0]

# Predict values

predictedBreakPointVal = np.array(r.predict(segModHR, newdata = breakpointTime))

#plt.plot(np.append(np.arange(times.min(),times.max()),breakPoint),np.append(predictedVals,predictedBreakPointVal), 'o')

# Sort to show segmented regression including appropriate breakpoint.

timeSort = np.append(np.arange(times.min(),times.max()),breakPoint)

sortedTimes = timeSort[np.argsort(timeSort)]

predicted = np.append(predictedVals,predictedBreakPointVal)

sortedPredicted = predicted[np.argsort(timeSort)]

# Pad predicted values with NaNs to shift appropriately.

startTime = np.min(np.where((~np.isnan(HRData))))

endTime = np.max(np.where((~np.isnan(HRData))))

#endHR = np.argmax(~np.isnan(HRData[::-1]))

predictedHRArray = np.zeros(len(HRData))

predictedHRArray[:] = np.NaN

predictedHRArray[times.min():times.max()] = predictedVals

# Get first predicted rate

firstHR = predictedHRArray[np.where(~np.isnan(predictedHRArray))[0][0]]

# Get last predicted rate

lastHR = predictedHRArray[np.where(~np.isnan(predictedHRArray))[0][-1]]

# Get rate at breakpoint

#breakpointHR = predictedHRArray[np.int(breakPoint)]

print 'Heart rate modelling for: ', str(self.embryo)

print 'Breakpoint HR: ', str(predictedBreakPointVal[0]), ', Slope 1: ', str(slopeOne), ', Slope 2: ', str(slopeTwo)

print 'Heart rate detected: ', str(startTime), 'Breakpoint occured: ', str(pyBreakpointTime), 'Heart rate no longer detected: ', str(endTime)

print 'Start heart rate: ', str(firstHR), ', End heart rate: ', str(lastHR)

outputSummary = [str('Heart rate modelling for: '+ str(self.embryo)),

str('Breakpoint HR: '+ str(predictedBreakPointVal[0])+ ' Slope 1: '+ str(slopeOne)+ ' Slope 2: '+ str(slopeTwo)),

str('Start heart rate: '+ str(firstHR)+ ' End heart rate: '+ str(lastHR))]

return list(predictedHRArray), list(predictedBreakPointVal), pyBreakpointTime, slopeOne, slopeTwo, startTime, endTime, firstHR, lastHR, list(HRData),outputSummary

def identifyLethalEndPoints(self, savePath, developmentalStage):

"""

Identify lethal end points. Currently optimised and tested for three

developmental stages of Radix balthica, employing different strategies

for each (drops in frequency energy, peaks in size indicative of a

failure in osmotic control and a combination of both).

"""

# If developmentalStage is late hippo identify drops in frequency energy

if developmentalStage is 'latehippo':

self.lethalEndPoint_data = dict()

for e in range(len(self.embryoLabels)):

self.embryo = self.embryoLabels[e]

self.loadXRResults()

# Extract required data from TimeSpecificSummaryData component of XArray dataset

totalLower = np.nansum(self.results['TimeSpecificSummaryData'].to_pandas().ix[:72,13:17],axis=1)

avHigher = np.nanmean(self.results['TimeSpecificSummaryData'].to_pandas().ix[:72,18:],axis=1)

# Normalise - this seems to work well.

data = pd.DataFrame(totalLower/avHigher).interpolate(limit_direction = 'both').values.ravel()

data = pd.DataFrame(data).interpolate(limit_direction = 'both').values.ravel()

# Identify where energy levels fall below threshold - likely to require diff

# thresholds for diff stages etc - first filter.

inds = np.where(np.log(data) < 3)

# If more than one drop identified - take just the first (falling below)

if len(inds[0]) > 1:

lethalIndex = np.min(inds[0])

lethalTime = self.results['TimeSpecificSummaryData'].to_pandas().index[lethalIndex]

self.lethalEndPoint_data[str(self.embryo)] = {'Embryo': self.embryo,'Dev stage': str(developmentalStage),'LethalTime': lethalTime,'LethalIndex': lethalIndex}

else:

# Identify where energy levels fall below threshold - likely to require diff

# thresholds for diff stages etc - second filter.

inds = np.where(np.log(data) < 3.3)

if len(inds[0]) > 1:

lethalIndex = np.min(inds[0])

lethalTime = self.results['TimeSpecificSummaryData'].to_pandas().index[lethalIndex]

self.lethalEndPoint_data[str(self.embryo)] = {'Embryo': self.embryo,'Dev stage': str(developmentalStage),'LethalTime': lethalTime,'LethalIndex': lethalIndex}

else:

lethalIndex = np.NaN

self.lethalEndPoint_data[str(self.embryo)] = {'Embryo': self.embryo,'Dev stage': str(developmentalStage),'LethalTime': np.NaN,'LethalIndex': lethalIndex}

np.save(savePath + '/lethalEndPoints.npy', self.lethalEndPoint_data)

# If developmentalStage is mid hippo first attempt to identify drops in frequency energy

# And failing this try peaks in size indicative of a failure in osmotic control.

if developmentalStage is 'midhippo':

self.lethalEndPoint_data = dict()

for e in range(len(self.embryoLabels)):

self.embryo = self.embryoLabels[e]

self.loadXRResults()

# Extract required data from TimeSpecificSummaryData component of XArray dataset

totalLower = np.nansum(self.results['TimeSpecificSummaryData'].to_pandas().ix[:,13:17],axis=1)

avHigher = np.nanmean(self.results['TimeSpecificSummaryData'].to_pandas().ix[:,18:],axis=1)

# Normalise - this seems to work well.

#data = pd.DataFrame(totalLower/avHigher).interpolate(limit_direction = 'both').values.ravel()

#data = pd.DataFrame(data).interpolate(limit_direction = 'both').values.ravel()

# Identify where energy levels fall below threshold - likely to require diff

# thresholds for diff stages etc - first filter.

inds = np.where(np.log(totalLower/avHigher) < 3)

# If more than one drop identified - take just the first (falling below)

if len(inds[0]) > 1:

lethalIndex = np.min(inds[0])

lethalTime = self.results['TimeSpecificSummaryData'].to_pandas().index[lethalIndex]

self.lethalEndPoint_data[str(self.embryo)] = {'Embryo': self.embryo,'Dev stage': str(developmentalStage),'LethalTime': lethalTime,'LethalIndex': lethalIndex}

#print str(lethalIndex)

else:

self.lethalEndPoint_data[str(self.embryo)] = {'Embryo': self.embryo,'Dev stage': str(developmentalStage),'LethalTime': np.NaN,'LethalIndex': np.NaN}

# If no lethal end point has been found also check for peaks in embryo size

# indicative of a loss of osmotic control

for e in range(len(self.embryoLabels)):

self.embryo = self.embryoLabels[e]

self.loadXRResults()

if self.lethalEndPoint_data[str(self.embryo)]['LethalTime'] is np.NaN:

data = pd.rolling_mean(self.results['TimeSpecificSummaryData'].to_pandas().ix[:,0],window=6)

if np.count_nonzero(np.isnan(data))/np.float(len(data)) > 0.7:

self.lethalEndPoint_data[str(self.embryo)] = {'Embryo': self.embryo,'Dev stage': str(developmentalStage),'LethalTime': np.NaN,'LethalIndex': np.NaN}

else:

# Interpolate to fill missing data

data = pd.DataFrame(data).interpolate(limit_direction = 'both').values.ravel()

#print np.count_nonzero(np.isnan(data))

# Identify peaks/loss of embryo osmotic control

indexes = peakutils.indexes(data-peakutils.baseline(data), thres=0.99, min_dist=1)

if len(indexes) == 1:

# Check that the peak is sufficiently distant from the baseline..

# Assess proportionate distance of peak from baseline.

baseline = peakutils.baseline(data)

propDist = np.abs(baseline-data[indexes]).min()/np.mean(baseline)

#print propDist

if propDist < 0.2:

self.lethalEndPoint_data[str(self.embryo)] = {'Embryo': self.embryo,'Dev stage': str(developmentalStage),'LethalTime': np.NaN,'LethalIndex': np.NaN}

else:

lethalTime = self.results['TimeSpecificSummaryData'].to_pandas().index[indexes[0]]

lethalIndex = indexes[0]

self.lethalEndPoint_data[str(self.embryo)] = {'Embryo': self.embryo,'Dev stage': str(developmentalStage),'LethalTime': lethalTime,'LethalIndex': lethalIndex}

#print str(lethalIndex)

#print 'Lethal end point identified via peak in area'

if len(indexes) > 1:

baseline = peakutils.baseline(data)

index = np.argmax(data[indexes])

# Check that the peak is sufficiently distant from the baseline..

# Assess proportionate distance of peak from baseline.

propDist = np.abs(baseline-data[indexes][index]).min()/np.mean(baseline)

print propDist

if propDist < 0.2:

#print 'No peak found'

self.lethalEndPoint_data[str(self.embryo)] = {'Embryo': self.embryo,'Dev stage': str(developmentalStage),'LethalTime': np.NaN,'LethalIndex': np.NaN}

else:

#mode[e] = 'ar'

lethalTime = self.results['TimeSpecificSummaryData'].to_pandas().index[index]

lethalIndex =index

self.lethalEndPoint_data[str(self.embryo)] = {'Embryo': self.embryo,'Dev stage': str(developmentalStage),'LethalTime': lethalTime,'LethalIndex': lethalIndex}

#print str(lethalIndex)

#print 'Lethal end point identified via peak in area'

if len(indexes) < 1:

self.lethalEndPoint_data[str(self.embryo)] = {'Embryo': self.embryo,'Dev stage': str(developmentalStage),'LethalTime': np.NaN,'LethalIndex': np.NaN}

#print 'Cant be sure'

# If developmentalStage is trochophore identify drops in frequency energy

if developmentalStage is 'trochophore':

self.lethalEndPoint_data = dict()

for e in range(len(self.embryoLabels)):

self.embryo = self.embryoLabels[e]

self.loadXRResults()

data = pd.rolling_mean(self.results['TimeSpecificSummaryData'].to_pandas().ix[:,0],window=2)

if np.count_nonzero(np.isnan(data))/np.float(len(data)) > 0.7:

print 'Not enough data for ' + str(self.embryo)

self.lethalEndPoint_data[str(self.embryo)] = {'Embryo': self.embryo,'Dev stage': str(developmentalStage),'LethalTime': np.NaN,'LethalIndex': np.NaN}

else:

# Interpolate to fill missing data

data = pd.DataFrame(data).interpolate(limit_direction = 'both').values.ravel()

# Identify peaks/loss of embryo osmotic control

indexes = peakutils.indexes(data-peakutils.baseline(data), thres=0.9, min_dist=1)

if len(indexes) == 1:

# Check that the peak is sufficiently distant from the baseline..

# Assess proportionate distance of peak from baseline.

baseline = peakutils.baseline(data)

propDist = np.abs(baseline-data[indexes]).min()/np.mean(baseline)

if propDist < 0.1:

self.lethalEndPoint_data[str(self.embryo)] = {'Embryo': self.embryo,'Dev stage': str(developmentalStage),'LethalTime': np.NaN,'LethalIndex': np.NaN}

else:

#lethalIndex = indexes[0]-1

lethalIndex = indexes[0]-1

lethalTime = self.results['TimeSpecificSummaryData'].to_pandas().index[lethalIndex]

self.lethalEndPoint_data[str(self.embryo)] = {'Embryo': self.embryo,'Dev stage': str(developmentalStage),'LethalTime': lethalTime,'LethalIndex': lethalIndex}

if len(indexes) > 1:

baseline = peakutils.baseline(data)

index = np.argmax(data[indexes])

# Check that the peak is sufficiently distant from the baseline..

# Assess proportionate distance of peak from baseline.

propDist = np.abs(baseline-data[indexes][index]).min()/np.mean(baseline)

if propDist < 0.1:

self.lethalEndPoint_data[str(self.embryo)] = {'Embryo': self.embryo,'Dev stage': str(developmentalStage),'LethalTime': np.NaN,'LethalIndex': np.NaN}

else:

lethalIndex = indexes[index]-1

#lethalIndex = indexes[index]-1

lethalTime = self.results['TimeSpecificSummaryData'].to_pandas().index[lethalIndex]

self.lethalEndPoint_data[str(self.embryo)] = {'Embryo': self.embryo,'Dev stage': str(developmentalStage),'LethalTime': lethalTime,'LethalIndex': lethalIndex}

if len(indexes) < 1:

self.lethalEndPoint_data[str(self.embryo)] = {'Embryo': self.embryo,'Dev stage': str(developmentalStage),'LethalTime': np.NaN,'LethalIndex': np.NaN}

# Visualise and save output

for keys,values in sorted(self.lethalEndPoint_data.items()):

print(keys)

print(values)

np.save(savePath + '/lethalEndPoints.npy', self.lethalEndPoint_data)

**eggUI.py**

from pyqtgraph.Qt import QtCore, QtGui

import numpy as np

from scipy.spatial import distance as dist

import glob

import re

import os

from PyQt5 import QtGui

from PyQt5.QtCore import *

from PyQt5.QtGui import *

import sys

import cv2

import pandas as pd

from PyQt5.Qt import *

import pyqtgraph as pg

#from PyQt4.Qt import *

#%%

class eggUI(QDialog):

'''

createOpenCVEggROI : take eggID defined ROIs and visualise

'''

sliderUpdate = QtCore.pyqtSignal()

embryoUpdate = QtCore.pyqtSignal()

keyPressed = QtCore.pyqtSignal()

def __init__(self, parent=None):

super(eggUI, self).__init__(parent)

# Make QDialog

self.diag = QtGui.QDialog()

global parentPath, vidTime

self.diag.setWindowTitle('Identify eggs')

self.diag.imv = pg.ImageView()

self.btn_save = QPushButton('Save', self)

#==============================================================================

#

#==============================================================================

def showUI(self,ims,eggRotBBox, eggBoxPoints, embryoLabels, eggInt):

self.eggInt = eggInt

self.embryoLabels = embryoLabels

self.diag.setWindowTitle('Identify eggs')

# Make ImageView

self.diag.imv = pg.ImageView()

self.diag.resize(1000,600)

# Make ROI

self.importOpenCVROIs(eggRotBBox, eggBoxPoints)

if (eggRotBBox[0][0][0] != 'nan'):

self.createOpenCVEggROI()

self.diag.imv.addItem(self.roi)

# Remove buttons from ImageView widget

self.diag.imv.ui.roiBtn.hide()

self.diag.imv.ui.menuBtn.hide()

# Make tableview

self.diag.table = QtGui.QTableWidget()

self.diag.table.setShowGrid(True)

self.diag.table.setHorizontalHeaderLabels(['Embryo', 'Sorted'])

# Sets different alignment data just on the first column

self.diag.table.setRowCount(int(len(self.embryoLabels)))

self.diag.table.setColumnCount(2)

# Highlight first row

self.diag.table.selectRow(0)

# Make layout

checkLayout = QGridLayout()

# Deal with stretching for approrpraite formatting.

checkLayout.setColumnStretch(0, 3)

checkLayout.setColumnStretch(1, 1)

checkLayout.setRowStretch(0, 1)

checkLayout.setRowStretch(1, 3)

# Add to layout

checkLayout.addWidget(self.diag.imv,0,0,2,2)

checkLayout.addWidget(self.diag.table,1,5)

# Apply layout

self.diag.setLayout(checkLayout)

# Make buttons

self.cpROI_btn = QtGui.QPushButton('&Copy ROI')

self.cpROI_btn.setMinimumHeight(40);

self.useCpROI_btn = QtGui.QPushButton('&Use Copied ROI')

self.useCpROI_btn.setMinimumHeight(40);

self.noEgg_btn = QtGui.QPushButton('&No Egg')

self.noEgg_btn.setMinimumHeight(40);

self.approveROI_btn = QtGui.QPushButton('&Approve ROIs')

self.approveROI_btn.setMinimumHeight(40);

self.exit_btn = QtGui.QPushButton('Exit')

self.exit_btn.setMinimumHeight(40);

# Make button layout

self.btnLayout = QGridLayout()

self.btnLayout.addWidget(self.cpROI_btn,0,0)

self.btnLayout.addWidget(self.useCpROI_btn,0,1)

self.btnLayout.addWidget(self.noEgg_btn,1,1)

self.btnLayout.addWidget(self.approveROI_btn,1,0)

# Exit button not implemented, just use window x (topRight).

# self.btnLayout.addWidget(self.exit_btn,2,1)

# Add button layout to GridLayout.

checkLayout.addLayout(self.btnLayout,0,5)

# Format images for pyqtgraph and put in ImageView

# self.formatSequence(ims)

self.imImport()

self.diag.imv.setImage(self.compSeq)

# Add the ROI to ImageItem

self.diag.show()

# Call function to add data

self.dataForTable()

# Function for modifying the table when ROI is approved.

self.approveROI_btn.clicked.connect(self.updateTable)

# Copy current ROI

self.cpROI_btn.clicked.connect(self.cpROI)

# Apply copied ROI

self.useCpROI_btn.clicked.connect(self.applyCopiedROI)

# Assign nan to frames not containing egg

self.noEgg_btn.clicked.connect(self.recordNoEgg)

# Exit - prompt user to confirm

#self.exit_btn.clicked.connect(self.closeEvent)

# Connect changes in timeline so correct ROI is created and displayed.

self.diag.imv.timeLine.sigPositionChanged.connect(self.updateOpenCVEggROICurrEmbryo)

#self.diag.keyPressEvent(self.keyPressEvent)

#==============================================================================

# Generate data for populating the embryo/approveROI table.

#==============================================================================

def dataForTable(self):

self.tableData = {'Embryo':list(self.embryoLabels),

'ROI approved':['No'] * len(list(self.embryoLabels))}

self.tableCols = [QtGui.QColor(0,0,100,120)]* len(list(self.embryoLabels))

# Enter data onto Table

horHeaders = []

for n, key in enumerate(sorted(self.tableData.keys())):

horHeaders.append(key)

for m, item in enumerate(self.tableData[key]):

newitem = QtGui.QTableWidgetItem(item)

newitem.setBackground(QtGui.QColor(0,0,100,120))

self.diag.table.setItem(m, n, newitem)

# Add Header

self.diag.table.setHorizontalHeaderLabels(horHeaders)

# Adjust size of Table

self.diag.table.resizeRowsToContents()

# self.diag.table.resizeColumnsToContents()

#==============================================================================

# Update table when approve ROI button clicked.

#==============================================================================

def updateTable(self):

self.tableData['ROI approved'][self.diag.table.currentRow()] = 'Approved'

self.tableCols[self.diag.table.currentRow()] = QtGui.QColor(0,100,0,120)

horHeaders = []

for n, key in enumerate(sorted(self.tableData.keys())):

horHeaders.append(key)

for m, item in enumerate(self.tableData[key]):

newitem = QtGui.QTableWidgetItem(item)

self.diag.table.setItem(m, n, newitem)

newitem.setBackground(self.tableCols[m])

#Add Header

self.diag.table.setHorizontalHeaderLabels(horHeaders)

#Adjust size of Table

self.diag.table.resizeRowsToContents()

#==============================================================================

# Update the user interface

#==============================================================================

def updateUI(self,ims,eggRotBBox, eggBoxPoints):

self.imImport()

self.diag.imv.setImage(self.compSeq)

self.importOpenCVROIs(eggRotBBox, eggBoxPoints)

self.getSeqValsAndCurrROI()

self.updateOpenCVEggROINewEmbryo()

# Add the ROI to ImageItem

#self.diag.imv.addItem(self.roi)

#==============================================================================

# Deal with data from the dataHandling class

#==============================================================================

def formatSequence(self,ims):

# Format seq appropriately for pyqtgraph ROIs

self.tSeqd = np.zeros_like(ims)

for l in range(len(self.tSeqd)):

self.tSeqd[l] = ims[l].T

#==============================================================================

# Get folders for a particular embryo

#==============================================================================

def getEmbryoFolders(self, parentPath, embryo):

self.parentPath = parentPath

self.embryo = embryo

self.embryoFolders = glob.glob(parentPath + "*/" + embryo +"/")

self.embryoFolders.sort(key=os.path.getctime)

#==============================================================================

# Get image

#==============================================================================

def imImport(self):

for f in range(len(self.eggUIimPaths)):

im = cv2.imread(self.eggUIimPaths[f],cv2.IMREAD_ANYDEPTH)

ran = (im.max()-im.min())/255.

out = (im/ran)

out = out-out.min()

self.compSeq[int(f)] = out.astype(np.uint8)

self.compSeq[f] = self.compSeq[f].T

#==============================================================================

# Update image iteratively when slider moved

#==============================================================================

#==============================================================================

# def updateImage(self):

# self.getSeqValsAndCurrROI()

# #self.UI.compSeq[e*len(self.eggIDIms):(e*len(self.eggIDIms)+len(self.eggIDIms))] = self.seq

# #self.UI.comp(self.imImport(self.diag.imv.currentIndex()))

# im = cv2.imread(self.eggUIimPaths[self.diag.imv.currentIndex],cv2.IMREAD_ANYDEPTH)

# ran = (im.max()-im.min())/255.

# out = (im/ran)

# out = out-out.min()

# self.compSeq[self.diag.imv.currentIndex] = out.astype(np.uint8)

# self.diag.imv.setImage(self.compSeq.T)

# self.diag.imv.show()

# #========

#==============================================================================

#==============================================================================

# ROI functions

#==============================================================================

#==============================================================================

# Import OpenCV determined ROIs from dataHandling instance. Called from showUI and updateUI.

#==============================================================================

def importOpenCVROIs(self,eggRotBBox, eggBoxPoints):

self.eggRotBBox = eggRotBBox

self.eggBoxPoints = eggBoxPoints

self.originalEggRotBBox = eggRotBBox.copy()

self.originalEggBoxPoints = eggBoxPoints.copy()

#==============================================================================

# Get index values for ROI data.

#==============================================================================

def getSeqValsAndCurrROI(self):

# Calculate the indices for current frame

if self.eggInt != 1234:

self.divVal = self.diag.imv.currentIndex/float(len(self.eggRotBBox[1]))

self.intDivVal = int(self.divVal)

self.withinSeqVal = int((self.divVal - self.intDivVal)*len(self.eggRotBBox[self.intDivVal]))

self.currROI_eggRotBBox = self.eggRotBBox[self.intDivVal,self.withinSeqVal]

self.currROI_eggBoxPoints = self.eggBoxPoints[self.intDivVal,self.withinSeqVal]

else:

self.divVal = self.diag.imv.currentIndex

self.intDivVal = int(self.divVal)

self.currROI_eggRotBBox = self.eggRotBBox[0,self.intDivVal]

self.currROI_eggBoxPoints = self.eggBoxPoints[0,self.intDivVal]

#==============================================================================

# Generate a pyqtgraph ROI, using data from OpenCV.

#==============================================================================

def createOpenCVEggROI(self):

# Get relevant sequence position and ROI.

self.getSeqValsAndCurrROI()

if (self.currROI_eggRotBBox[0] != 'nan'):

# 0 or 90 degree angles seem very buggy. Shift to 1 and 89 as a bodge fix.

if self.currROI_eggRotBBox[4] == -90:

#self.currROI_eggRotBBox[4] = -89

# Get rotated bounding box points

ySorted = self.currROI_eggBoxPoints[np.argsort(self.currROI_eggBoxPoints[:, 1]), :]

# Get bottom most, and top most sorted corner points

bottomMost = ySorted[:2, :]

topMost = ySorted[2:, :]

# Get bottom most

bottomMost = bottomMost[np.argsort(bottomMost[:, 1]), :]

(bl, br) = bottomMost

# Use bottom-left coordinate as anchor to calculate the Euclidean distance between the

# The point with the largest distance will be our bottom-right point

D = dist.cdist(bl[np.newaxis], topMost, "euclidean")[0]

(tl, tr) = topMost[np.argsort(D)[::-1], :]

self.roi = pg.ROI([bl[0], bl[1]], [self.currROI_eggRotBBox[2], self.currROI_eggRotBBox[3]])

elif self.currROI_eggRotBBox[4] == -0:

#self.currROI_eggRotBBox[4] = -1

ySorted = self.currROI_eggBoxPoints[np.argsort(self.currROI_eggBoxPoints[:, 1]), :]

# Get bottom most, and top most sorted corner points

bottomMost = ySorted[:2, :]

topMost = ySorted[2:, :]

# Get bottom most

bottomMost = bottomMost[np.argsort(bottomMost[:, 1]), :]

(bl, br) = bottomMost

# Use bottom-left coordinate as anchor to calculate the Euclidean distance between the

# The point with the largest distance will be our bottom-right point

D = dist.cdist(bl[np.newaxis], topMost, "euclidean")[0]

(tl, tr) = topMost[np.argsort(D)[::-1], :]

self.roi = pg.ROI([bl[0], bl[1]], [self.currROI_eggRotBBox[2], self.currROI_eggRotBBox[3]])

elif self.currROI_eggRotBBox[4] == -180:

#self.currROI_eggRotBBox[4] = -179

ySorted = self.currROI_eggBoxPoints[np.argsort(self.currROI_eggBoxPoints[:, 1]), :]

# Get bottom most, and top most sorted corner points

bottomMost = ySorted[:2, :]

topMost = ySorted[2:, :]

# Get bottom most

bottomMost = bottomMost[np.argsort(bottomMost[:, 1]), :]

(bl, br) = bottomMost

# Use bottom-left coordinate as anchor to calculate the Euclidean distance between the

# The point with the largest distance will be our bottom-right point

D = dist.cdist(bl[np.newaxis], topMost, "euclidean")[0]

(tl, tr) = topMost[np.argsort(D)[::-1], :]

self.roi = pg.ROI([bl[0], bl[1]], [self.currROI_eggRotBBox[2], self.currROI_eggRotBBox[3]])

else:

# Get rotated bounding box points

ySorted = self.currROI_eggBoxPoints[np.argsort(self.currROI_eggBoxPoints[:, 1]), :]

# Get bottom most, and top most sorted corner points

bottomMost = ySorted[:2, :]

topMost = ySorted[2:, :]

# Get bottom most

bottomMost = bottomMost[np.argsort(bottomMost[:, 1]), :]

(bl, br) = bottomMost

# Use bottom-left coordinate as anchor to calculate the Euclidean distance between the

# The point with the largest distance will be our bottom-right point

D = dist.cdist(bl[np.newaxis], topMost, "euclidean")[0]

(tl, tr) = topMost[np.argsort(D)[::-1], :]

# Make ROI - note non 0,or 90 degree angles, require different of the X size

# Rectangular ROI used to enable more easy handling of corner handles for tracking user chagnges.

if (self.currROI_eggRotBBox[4] == -90.0) | (self.currROI_eggRotBBox[4] == -0.0)| (self.currROI_eggRotBBox[4] == 0.0):

self.roi = pg.ROI([bl[0], bl[1]], [self.currROI_eggRotBBox[2], self.currROI_eggRotBBox[3]])

# roi = pg.EllipseROI([bottomMost[0][0], bottomMost[0][1]], [eggRotBBox[vidTime][2], eggRotBBox[vidTime][3]])

# Debug

# print 'no angle'

else:

# Random angle ROIs

self.roi = pg.ROI([bottomMost[0][0], bottomMost[0][1]], [-self.currROI_eggRotBBox[2], self.currROI_eggRotBBox[3]])

self.roi.setAngle(self.currROI_eggRotBBox[4], update=True)

# roi = pg.EllipseROI([bottomMost[0][0], bottomMost[0][1]], [-eggRotBBox[vidTime][2], eggRotBBox[vidTime][3]])

# Add handles

self.roi.addRotateHandle([1, 0],[0.5,0.5])

self.roi.addRotateHandle([0, 1], [0.5,0.5])

self.roi.addScaleHandle([1, 1], [0, 0])

self.roi.addScaleHandle([0, 0], [1, 1])

self.roi.setPen('y',width=3)

self.roi.removable

self.roi.invertible = 'True'

# Make var for dealing with modifications to roi

self.updatedEggROI=[]

self.roi.sigRegionChangeFinished.connect(self.updateROI)

#else:

#==============================================================================

# Update the ROI for current embryo.

#==============================================================================

def updateOpenCVEggROICurrEmbryo(self):

# Remove previous

if (hasattr(self, 'roi')):

self.diag.imv.removeItem(self.roi)

# Get relevant video position and ROI.

self.getSeqValsAndCurrROI()

# 0 or 90 degree angles seem very buggy. Shift to 1 and 89 as a bodge fix.

if self.currROI_eggRotBBox[4] == -90:

#self.currROI_eggRotBBox[4] = -89

# Get rotated bounding box points

ySorted = self.currROI_eggBoxPoints[np.argsort(self.currROI_eggBoxPoints[:, 1]), :]

# Get bottom most, and top most sorted corner points

bottomMost = ySorted[:2, :]

topMost = ySorted[2:, :]

# Get bottom most

bottomMost = bottomMost[np.argsort(bottomMost[:, 1]), :]

(bl, br) = bottomMost

# Use bottom-left coordinate as anchor to calculate the Euclidean distance between the

# The point with the largest distance will be our bottom-right point

D = dist.cdist(bl[np.newaxis], topMost, "euclidean")[0]

(tl, tr) = topMost[np.argsort(D)[::-1], :]

self.roi = pg.ROI([bl[0], bl[1]], [self.currROI_eggRotBBox[2], self.currROI_eggRotBBox[3]])

elif self.currROI_eggRotBBox[4] == -0:

#self.currROI_eggRotBBox[4] = -1

ySorted = self.currROI_eggBoxPoints[np.argsort(self.currROI_eggBoxPoints[:, 1]), :]

# Get bottom most, and top most sorted corner points

bottomMost = ySorted[:2, :]

topMost = ySorted[2:, :]

# Get bottom most

bottomMost = bottomMost[np.argsort(bottomMost[:, 1]), :]

(bl, br) = bottomMost

# Use bottom-left coordinate as anchor to calculate the Euclidean distance between the

# The point with the largest distance will be our bottom-right point

D = dist.cdist(bl[np.newaxis], topMost, "euclidean")[0]

(tl, tr) = topMost[np.argsort(D)[::-1], :]

self.roi = pg.ROI([bl[0], bl[1]], [self.currROI_eggRotBBox[2], self.currROI_eggRotBBox[3]])

elif self.currROI_eggRotBBox[4] == -180:

#self.currROI_eggRotBBox[4] = -179

ySorted = self.currROI_eggBoxPoints[np.argsort(self.currROI_eggBoxPoints[:, 1]), :]

# Get bottom most, and top most sorted corner points

bottomMost = ySorted[:2, :]

topMost = ySorted[2:, :]

# Get bottom most

bottomMost = bottomMost[np.argsort(bottomMost[:, 1]), :]

(bl, br) = bottomMost

# Use bottom-left coordinate as anchor to calculate the Euclidean distance between the

# The point with the largest distance will be our bottom-right point

D = dist.cdist(bl[np.newaxis], topMost, "euclidean")[0]

(tl, tr) = topMost[np.argsort(D)[::-1], :]

self.roi = pg.ROI([bl[0], bl[1]], [self.currROI_eggRotBBox[2], self.currROI_eggRotBBox[3]])

else:

# Get rotated bounding box points

ySorted = self.currROI_eggBoxPoints[np.argsort(self.currROI_eggBoxPoints[:, 1]), :]

# Get bottom most, and top most sorted corner points

bottomMost = ySorted[:2, :]

topMost = ySorted[2:, :]

# Get bottom most

bottomMost = bottomMost[np.argsort(bottomMost[:, 1]), :]

(bl, br) = bottomMost

# Use bottom-left coordinate as anchor to calculate the Euclidean distance between the

# The point with the largest distance will be our bottom-right point

D = dist.cdist(bl[np.newaxis], topMost, "euclidean")[0]

(tl, tr) = topMost[np.argsort(D)[::-1], :]

# Make ROI - note non 0,or 90 degree angles, require different of the X size

# Rectangular ROI used to enable more easy handling of corner handles for tracking user chagnges.

if (self.currROI_eggRotBBox[4] == -90.0) | (self.currROI_eggRotBBox[4] == -0.0)| (self.currROI_eggRotBBox[4] == 0.0):

self.roi = pg.ROI([bl[0], bl[1]], [self.currROI_eggRotBBox[2], self.currROI_eggRotBBox[3]])

# roi = pg.EllipseROI([bottomMost[0][0], bottomMost[0][1]], [eggRotBBox[vidTime][2], eggRotBBox[vidTime][3]])

# Debug

# print 'no angle'

else:

# Random angle ROIs

self.roi = pg.ROI([bottomMost[0][0], bottomMost[0][1]], [-self.currROI_eggRotBBox[2], self.currROI_eggRotBBox[3]])

self.roi.setAngle(self.currROI_eggRotBBox[4], update=True)

# roi = pg.EllipseROI([bottomMost[0][0], bottomMost[0][1]], [-eggRotBBox[vidTime][2], eggRotBBox[vidTime][3]])

# roi = pg.EllipseROI([bottomMost[0][0], bottomMost[0][1]], [-eggRotBBox[vidTime][2], eggRotBBox[vidTime][3]])

# Add handles

self.roi.addRotateHandle([1, 0],[0.5,0.5])

self.roi.addRotateHandle([0, 1], [0.5,0.5])

self.roi.addScaleHandle([1, 1], [0, 0])

self.roi.addScaleHandle([0, 0], [1, 1])

self.roi.setPen('y',width=3)

self.roi.removable

self.roi.invertible = 'True'

# Make var for dealing with modifications to roi

self.updatedEggROI=[]

### Still to do...

self.diag.imv.addItem(self.roi)

self.roi.sigRegionChangeFinished.connect(self.updateROI)

#==============================================================================

# Update ROI for new embryo.

#==============================================================================

def updateOpenCVEggROINewEmbryo(self):

# Remove old ROI

if (hasattr(self, 'roi')):

self.diag.imv.removeItem(self.roi)

# Get relevant video position and ROI

self.getSeqValsAndCurrROI()

# 0 or 90 degree angles seem very buggy. Shift to 1 and 89 as a bodge fix.

if self.currROI_eggRotBBox[4] == -90:

#self.currROI_eggRotBBox[4] = -89

# Get rotated bounding box points

ySorted = self.currROI_eggBoxPoints[np.argsort(self.currROI_eggBoxPoints[:, 1]), :]

# Get bottom most, and top most sorted corner points

bottomMost = ySorted[:2, :]

topMost = ySorted[2:, :]

# Get bottom most

bottomMost = bottomMost[np.argsort(bottomMost[:, 1]), :]

(bl, br) = bottomMost

# Use bottom-left coordinate as anchor to calculate the Euclidean distance between the

# The point with the largest distance will be our bottom-right point

D = dist.cdist(bl[np.newaxis], topMost, "euclidean")[0]

(tl, tr) = topMost[np.argsort(D)[::-1], :]

self.roi = pg.ROI([bl[0], bl[1]], [self.currROI_eggRotBBox[2], self.currROI_eggRotBBox[3]])

elif self.currROI_eggRotBBox[4] == -0:

#self.currROI_eggRotBBox[4] = -1

ySorted = self.currROI_eggBoxPoints[np.argsort(self.currROI_eggBoxPoints[:, 1]), :]

# Get bottom most, and top most sorted corner points

bottomMost = ySorted[:2, :]

topMost = ySorted[2:, :]

# Get bottom most

bottomMost = bottomMost[np.argsort(bottomMost[:, 1]), :]

(bl, br) = bottomMost

# Use bottom-left coordinate as anchor to calculate the Euclidean distance between the

# The point with the largest distance will be our bottom-right point

D = dist.cdist(bl[np.newaxis], topMost, "euclidean")[0]

(tl, tr) = topMost[np.argsort(D)[::-1], :]

self.roi = pg.ROI([bl[0], bl[1]], [self.currROI_eggRotBBox[2], self.currROI_eggRotBBox[3]])

elif self.currROI_eggRotBBox[4] == -180:

#self.currROI_eggRotBBox[4] = -179

ySorted = self.currROI_eggBoxPoints[np.argsort(self.currROI_eggBoxPoints[:, 1]), :]

# Get bottom most, and top most sorted corner points

bottomMost = ySorted[:2, :]

topMost = ySorted[2:, :]

# Get bottom most

bottomMost = bottomMost[np.argsort(bottomMost[:, 1]), :]

(bl, br) = bottomMost

# Use bottom-left coordinate as anchor to calculate the Euclidean distance between the

# The point with the largest distance will be our bottom-right point

D = dist.cdist(bl[np.newaxis], topMost, "euclidean")[0]

(tl, tr) = topMost[np.argsort(D)[::-1], :]

self.roi = pg.ROI([bl[0], bl[1]], [self.currROI_eggRotBBox[2], self.currROI_eggRotBBox[3]])

else:

# Get rotated bounding box points

ySorted = self.currROI_eggBoxPoints[np.argsort(self.currROI_eggBoxPoints[:, 1]), :]

# Get bottom most, and top most sorted corner points

bottomMost = ySorted[:2, :]

topMost = ySorted[2:, :]

# Get bottom most

bottomMost = bottomMost[np.argsort(bottomMost[:, 1]), :]

(bl, br) = bottomMost

# Use bottom-left coordinate as anchor to calculate the Euclidean distance between the

# The point with the largest distance will be our bottom-right point

D = dist.cdist(bl[np.newaxis], topMost, "euclidean")[0]

(tl, tr) = topMost[np.argsort(D)[::-1], :]

# Make ROI - note non 0,or 90 degree angles, require different of the X size

# Rectangular ROI used to enable more easy handling of corner handles for tracking user chagnges.

if (self.currROI_eggRotBBox[4] == -90.0) | (self.currROI_eggRotBBox[4] == -0.0)| (self.currROI_eggRotBBox[4] == 0.0):

self.roi = pg.ROI([bl[0], bl[1]], [self.currROI_eggRotBBox[2], self.currROI_eggRotBBox[3]])

# roi = pg.EllipseROI([bottomMost[0][0], bottomMost[0][1]], [eggRotBBox[vidTime][2], eggRotBBox[vidTime][3]])

# Debug

# print 'no angle'

else:

# Random angle ROIs

self.roi = pg.ROI([bottomMost[0][0], bottomMost[0][1]], [-self.currROI_eggRotBBox[2], self.currROI_eggRotBBox[3]])

self.roi.setAngle(self.currROI_eggRotBBox[4], update=True)

# Add handles

self.roi.addRotateHandle([1, 0],[0.5,0.5])

self.roi.addRotateHandle([0, 1], [0.5,0.5])

self.roi.addScaleHandle([1, 1], [0, 0])

self.roi.addScaleHandle([0, 0], [1, 1])

self.roi.setPen('y',width=3)

self.roi.removable

self.roi.invertible = 'True'

# Make var for dealing with modifications to roi

self.updatedEggROI=[]

### Still to do...

self.diag.imv.addItem(self.roi)

self.roi.sigRegionChangeFinished.connect(self.updateROI)

#==============================================================================

# Update ROI.

#==============================================================================

def updateROI(self):

#global vidTime, xyPosHandles, ellipse, changeAngle, roiChanges,updatedEggROI, changeX, changeY, changeScaleX, changeScaleY, changeAngle

# Get changes to ROI scale, angle and position

roiChanges = self.roi.getGlobalTransform()

changeX = -roiChanges.getTranslation()[0]

changeY = roiChanges.getTranslation()[1]

changeScaleX = roiChanges.getScale()[0]

changeScaleY = roiChanges.getScale()[1]

changeAngle = roiChanges.getAngle()

# Update ROI, either updating the previously updated or taking the unaltered ROI from OpenCV as a starting point.

#if len(self.updatedEggROI) == 0:

self.updatedEggROI = (((self.currROI_eggRotBBox[0]-changeX),(self.currROI_eggRotBBox[1]+changeY)),((max((self.currROI_eggRotBBox[3]*changeScaleX),(self.currROI_eggRotBBox[2]*changeScaleY))),(min((self.currROI_eggRotBBox[3]*changeScaleX),(self.currROI_eggRotBBox[2]*changeScaleY)))),self.currROI_eggRotBBox[4]+changeAngle)

#else:

#self.updatedEggROI = (((self.updatedEggROI[0][0]-changeX),(self.updatedEggROI[0][1]+changeY)),((max((self.updatedEggROI[1][0]*changeScaleX),(self.updatedEggROI[1][1]*changeScaleY))),(min((self.updatedEggROI[1][0]*changeScaleX),(self.updatedEggROI[1][1]*changeScaleY)))),self.updatedEggROI[2]+changeAngle)

hh = self.roi.getHandles()

hh = [self.roi.mapToItem(self.diag.imv.getImageItem(), h.pos()) for h in hh]

# Handle on each corner. Get handle positions

self.xyPosHandles =[]

for h in hh:

self.xyPosHandles.append([h.x(),h.y()])

(eggBBX, eggBBY), (eggBBW, eggBBH), eggBBAng = cv2.minAreaRect(np.array(self.xyPosHandles, dtype=np.int32) )

if eggBBAng == -90:

eggBBAng = -89

elif eggBBAng == -180:

eggBBAng = -179

elif eggBBAng == -0:

eggBBAng = -1

# Save updated

# If more than one frame eggID per sequence..

if self.eggInt != 1234:

self.eggRotBBox[self.intDivVal,self.withinSeqVal] = [eggBBX, eggBBY, eggBBW, eggBBH, eggBBAng]

self.eggBoxPoints[self.intDivVal,self.withinSeqVal] = cv2.boxPoints(((eggBBX, eggBBY), (eggBBW, eggBBH), eggBBAng))

# Otherwise just save simply

else:

self.eggRotBBox[0,self.intDivVal] = [eggBBX, eggBBY, eggBBW, eggBBH, eggBBAng]

self.eggBoxPoints[0,self.intDivVal] = cv2.boxPoints(((eggBBX, eggBBY), (eggBBW, eggBBH), eggBBAng))

#==============================================================================

# Copy ROI on button click.

#==============================================================================

def cpROI(self):

self.originalEggRotBBox = self.currROI_eggRotBBox

self.originalEggBoxPoints = self.currROI_eggBoxPoints

#==============================================================================

# Assign nan to current ROI if 'No Egg' button clicked

#==============================================================================

def recordNoEgg(self):

# Remove ROI

self.diag.imv.removeItem(self.roi)

# Store nans in place of ROI

if self.eggInt != 1234:

self.eggRotBBox[self.intDivVal,self.withinSeqVal] = [np.nan, np.nan, np.nan, np.nan, np.nan]

self.eggBoxPoints[0,self.intDivVal] = [np.nan,np.nan,np.nan,np.nan]

else:

self.eggBoxPoints[0,self.intDivVal] = [np.nan,np.nan,np.nan,np.nan]

self.eggRotBBox[0,self.intDivVal] = [np.nan, np.nan, np.nan, np.nan, np.nan]

#==============================================================================

# Copy ROI on button click.

#==============================================================================

def applyCopiedROI(self):

self.getSeqValsAndCurrROI()

# Store copied ROI to embryo sequence ROIs

if self.eggInt != 1234:

self.divVal = self.diag.imv.currentIndex/float(len(self.eggRotBBox[1]))

self.intDivVal = int(self.divVal)

self.withinSeqVal = int((self.divVal - self.intDivVal)*len(self.eggRotBBox[self.intDivVal]))

self.eggRotBBox[self.intDivVal,self.withinSeqVal] = self.originalEggRotBBox

self.eggBoxPoints[self.intDivVal,self.withinSeqVal] = self.originalEggBoxPoints

else:

self.divVal = self.diag.imv.currentIndex

self.intDivVal = int(self.divVal)

self.eggRotBBox[0,self.intDivVal] = self.originalEggRotBBox

self.eggBoxPoints[0,self.intDivVal] = self.originalEggBoxPoints

self.updateOpenCVEggROICurrEmbryo()

#==============================================================================

#

#==============================================================================

#==============================================================================

# Close button - not implemented (hidden)

#==============================================================================

#==============================================================================

# def closeEvent(self, event):

#

# quit_msg = "Are you sure you want to exit the program?"

# reply = QtGui.QMessageBox.question(self, 'Message',

# quit_msg, QtGui.QMessageBox.Yes, QtGui.QMessageBox.No)

#

# if reply == QtGui.QMessageBox.Yes:

# #event.accept()

# app.quit()

# else:

# event.ignore()

#

#==============================================================================

#==============================================================================

# #self.originalEggRotBBox = eggRotBBox.copy()

# #self.originalEggBoxPoints = eggBoxPoints.copy()

# #self.currROI_eggRotBBox = self.eggRotBBox[self.intDivVal,self.withinSeqVal]

# #self.currROI_eggBoxPoints = self.eggBoxPoints[self.intDivVal,self.withinSeqVal]

#

# # Modified version of updateOpenCVEggROICurrEmbryo

# # Remove previous

# self.diag.imv.removeItem(self.roi)

# # Get relevant video position and ROI.

# self.getSeqValsAndCurrROI()

# # Get rotated bounding box points

# ySorted = self.originalEggBoxPoints[np.argsort(self.originalEggBoxPoints[:, 1]), :]

# # Get bottom most, and top most sorted corner points

# bottomMost = ySorted[:2, :]

# topMost = ySorted[2:, :]

# # Get bottom most

# bottomMost = bottomMost[np.argsort(bottomMost[:, 1]), :]

# (bl, br) = bottomMost

# # Use bottom-left coordinate as anchor to calculate the Euclidean distance between the

# # The point with the largest distance will be our bottom-right point

# D = dist.cdist(bl[np.newaxis], topMost, "euclidean")[0]

# (tl, tr) = topMost[np.argsort(D)[::-1], :]

# # Make ROI - note non 0,or 90 degree angles, require different of the X size

# # Rectangular ROI used to enable more easy handling of corner handles for tracking user chagnges.

# if (self.originalEggRotBBox[4] == -90.0) | (self.originalEggRotBBox[4] == -0.0)| (self.originalEggRotBBox[4] == 0.0):

# self.roi = pg.ROI([bottomMost[0][0], bottomMost[0][1]], [self.originalEggRotBBox[2], self.originalEggRotBBox[3]])

# # roi = pg.EllipseROI([bottomMost[0][0], bottomMost[0][1]], [eggRotBBox[vidTime][2], eggRotBBox[vidTime][3]])

# else:

# # Random angle ROIs

# self.roi = pg.ROI([bottomMost[0][0], bottomMost[0][1]], [-self.originalEggRotBBox[2], self.originalEggRotBBox[3]])

# self.roi.setAngle(self.originalEggRotBBox[4], update=True)

# # roi = pg.EllipseROI([bottomMost[0][0], bottomMost[0][1]], [-eggRotBBox[vidTime][2], eggRotBBox[vidTime][3]])

# # Add handles

# self.roi.addRotateHandle([1, 0],[0.5,0.5])

# self.roi.addRotateHandle([0, 1], [0.5,0.5])

# self.roi.addScaleHandle([1, 1], [0, 0])

# self.roi.addScaleHandle([0, 0], [1, 1])

# self.roi.setPen('y',width=3)

# self.roi.removable

# self.roi.invertible = 'True'

# # Make var for dealing with modifications to roi

# self.updatedEggROI=[]

# ### Still to do...

# self.diag.imv.addItem(self.roi)

# self.roi.sigRegionChangeFinished.connect(self.updateROI)

#======================================================================
