# Supplementary material for: A high-throughput and open-source platform for embryo phenomics
Source: PLoS Biol. 2018 Dec 13;16(12):e3000074. doi: 10.1371/journal.pbio.3000074 (PMC6292576; doi:10.1371/journal.pbio.3000074)
Supplement: S4 Code — (DOCX) [file pbio.3000074.s004.docx]

**Supplementary 4. ImageJ macro used for recording beat to beat timings in cardiac activity.**

macro "InstanceRecord [i]" {

print(getTitle());

id=getImageID();

lastSlice=-2;

while(true) {

slice = getSliceNumber();

if(isKeyDown("space") && slice>(lastSlice + 2)) {

if(getImageID()!=id) exit("different image, recording of frames stopped");

print(slice); beep();

lastSlice=slice;

}

wait(100);

}
