# Supplementary material for: A high-throughput and open-source platform for embryo phenomics
Source: PLoS Biol. 2018 Dec 13;16(12):e3000074. doi: 10.1371/journal.pbio.3000074 (PMC6292576; doi:10.1371/journal.pbio.3000074)
Supplement: S1 Table — OpenVIM, Open-source Video Microscope. (PDF) [file pbio.3000074.s006.pdf]

OpenVIM

Open-source-video microscope

Component	Experiments (current, or previous publications)	Specification	Notes
Lenses			
Keyence VHZ20R	• Used in experiments 1 – 4.<br>• Supported [1, 2, 3]	20 – 200 X magnification	Extremely high depth of field lens, excellent for low magnification imaging of aquatic embryos, 20-200 X digital magnification. **Approx cost** = £5,000
Keyence VHZ100R	• Supported [3, 4]	100 – 1000 X magnification	High depth of field 100 - 1000 X digital magnification lens. **Approx cost** = £6,000
Keyence VHZ500R		500 – 5000 X magnification	High magnification lens, good for imaging Protozoa. **Approx cost** = £6,500
Qioptiq Optem Micro Lens	• Supported [5, 6, 7]	Wide range of operating possibilities due to being highly customisable.	Highly modular lens system with a wide range of magnification using combinations of auxillary lenses and TV tubes. **Approx cost** = from £2,000
Lighting			
CCS LDR2-42-SW2	• Used in experiments 1 - 4	An angled 42 mm OD LED ring light for focussed dark field lighting	**Approx cost** = £600 (with power supply)
Keyence CA-DRW4F	• Supported [1, 3, 7, 8]	A non-focussed 43 mm OD LED ring light.	**Approx cost** = £600 (with power supply)

Schott EasyLED gooseneck lights		Gooseneck spotlights, can be mounted on optic stainless steel support pole.	A useful lighting apparatus for imaging different types of speciment, although less suited to repeatable lighting. **Approx cost** = £300
Motorised stages			
Marzhauser SCAN IM 130 x 85	• Supported [2]	A motorised stage with 130 x 85 mm of travel – sufficient for a multiwell plate.	The Marzhauser Scan Tango XY stage is a very reliable and robust stage with very good technical and MicroManager support. Platform independant MicroManager support. Controlled with the Tango Desktop controller **Approx cost** = £7,000 (with controller)
Prior ES111 Optiscan stage	• Supported [1, 3, 7, 8]	A motorised stage with 125 x 75 mm of travel – sufficient for a multiwell plate.	A cost effective stage with platform independant MicroManager support. Controlled with previous versions of the ES11 Controller. **Approx cost** = £6,000 (with controller)
Prior ES107 Inverted Optiscan stage		A motorised stage with 114 x 75 mm of travel – sufficient for a multiwell plate. Inverted so suitable for reduced working distance optics.	An inverted XY stage, good for samples requiring high magnification to get the necessarily small working distance required, for example using the VHZ500R lens. Note that this stage requires different support leg offsets

			in the alumnium base owing to its greater footprint. **Approx cost** = £8,000 (with controller)
Cameras			
Allied Vision Technologies Pike 421B	• Used in experiments 1 – 4<br>• Supported [1 ,3, 4, 7, 8]	Monochrome 2048 x 2048, 7.4 µm pixels.	An excellent camera for darkfield imaging, used with dc1394b FW800 connection. Great MicroManager support owing to new developments with the IIDC driver. Allows custom ROI, recording of full 14 bits and precise manipulation of frame rates via control over packet size. Image capture time is recorded with high precision. **Approx cost** = £5,000
Allied Vision Technologies Pike 210C	• Supported [5]	Color 1980 x 1080, 7.4 µm pixels	A lower resolution model of the Pike 421C colour camera **Approx cost** = £5,000
The Imaging Source		Resolution from 0.4-5 MP. Monochrome and colour.	Various colour and monochrome models from the DMK range successfully used. **Approx cost** = from £300
QImaging Retiga (R3 and R6)		QImaging R3 = 3MP and QImaging R6 = 6 MP. Monochrome and colour.	USB 3.0 cameras available in monochrome and colour, with excellent image quality. However, note that drivers for

			MicroManager are only available for Windows. **Approx cost** = £4,000 / £4,500
Incubation chambers			
Oko-lab Cryo Boldline	• Used in experiments 1 – 4<br>• Supported [1, 9, 10]	Min T = 10-15 °C below ambient, Max T = 60 °C, sample resolution = 0.01 °C . Humidity > 90%. Compatible with digital and manual OKO-Lab gas mixers.	Enables heating and cooling of samples in multiwell plates, with humidification (to reduce evaporation) and excellent data logging facilities. Can also be used with gas mixing modules, including combined mixing of nitrogen, oxygen and carbon dioxide. OKO-Labs can design gas mixing units to user specifications if required. **Approx cost** = £15,000
Oko-lab electrically heated chamber	• Supported [2]	Min T = 3 °C above ambient, Max T = 60 °C. Sample resolution = 0.1 °C Humidity > 90 %.	Allows heating of sample within multiwell plates using electrical filament heaters built into an incubation chamber. System integrates this with data logging and controlled temperature humidification. This particular model is discontinued. **Approx cost** = £6,000
Custom parts			
Aluminium base	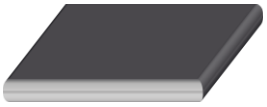	**Material** : HE9 alumnium plate - 20 mm x 300 mm x 220 mm	

		**Material cost** : £65<br><br>**Machining** : Four tapped holes added to underside for attaching threaded feet (aids in level and keeping stable), and four untapped holes with centres matching the mounting points of the motorised stage - to which the aluminium legs will attach.	
Aluminium legs	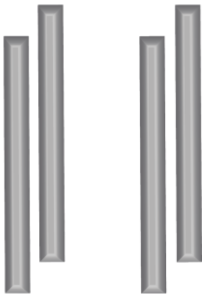	**Material** : HE9 alumnium pole - 25 mm diameter x 600 mm<br><br>**Material cost** : Approx £45<br><br>**Machining** : Both ends of each leg should be tapped to allow attachment to the motorised stage and alumnium base - via machine bolt	
Aluminium optic support block	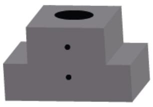	**Material** : HE9 alumnium block - 50 mm x 100 mm x 50 mm.<br><br>**Material cost** : Approx £30<br><br>**Machining** : Four vertical holes for attaching to the alumnium base via machine bolt, one large vertical hole to receive the stainless steel optic pole and two horizontal	

		threaded holes for grub screw tightening of the stainless steel optic pole.	
Stainless steel optic pole	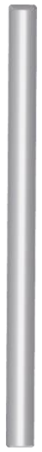	**Material** : 304 stainless steel pole, 15 mm diameter x 900 mm<br><br>**Material cost** : Approx £18<br><br>**Machining** : No machining necessary, unless cutting to length is required	
Delrin lens support mount	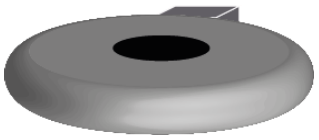	**Material** : Delrin thermoplastic - 150 mm diameter x 100 mm length<br><br>**Material cost** : Approx £50<br><br>**Machining** : Delrin machined to a circular profile using a laithe. The profile should be circular (diameter to suit the optics carrier chosen - see below) have a central hole for receipt of the lens (see below), a horizontal slit for tightening, using a recessed machine bolt, and a lip on the upper side so that it is supported by the optics carrier component that is chosen.	

Acrylic light mount	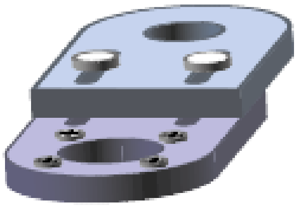	**Material** : Acrylic 10 mm x 200 mm x 100 mm<br><br>**Material cost** : Approx £5<br><br>**Machining** : Acrylic lighting mount, best produced using a CNC machine. Profile tailored to suit the light chosen and it is mounted on the stainless steel optic pole, secured using a grub screw and subsequently can be moved both vertically and laterally for optimisation and convenience.	

References

1. Tills O, Sun X, Rundle SD, Heimbach T, Gibson T, Cartwright A, et al. Reduced pH affects pulsing behaviour and body size in ephyrae of the moon jellyfish, *Aurelia aurita*. JEMBE. 2016; 480: 54-61.
2. Truebano M, Fenner P, Tills O, Rundle SD, Rezende EL. Thermal strategies vary with life history stage. JEB. 2018; 221:171629.
3. Tills O, Bitterli T, Culverhouse P, Spicer JI, Rundle SD. A novel application of motion analysis for detecting stress responses in embryos at different stages of development. BMC Bioinformatics. 2013;14: 37.
4. Rudin-Bitterli TS, Tills O, Spicer JI, Culverhouse PF, Wielhouwer EM, Richardson MK, et al. Combining motion analysis and microfluidics--a novel approach for detecting whole-animal responses to test substances. PLOS One. 2014; 9: e113235.
5. Hallett KC, Atfield A, Comber S, Hutchinson TH. Developmental toxicity of metaldehyde in the embryos of *Lymnaea stagnalis* (Gastropoda: Pulmonata) co-exposed to the synergist piperonyl butoxide. Sci Tot Environ. 2016; 543: 37-43.
6. Rudin-Bitterli TS, Spicer JI, Rundle SD. Differences in the timing of cardio-respiratory development determine whether marine gastropod embryos survive or die in hypoxia. JEB. 2016; 219: 1076-1085.
7. Tills O Rundle SD, Spicer JI. Variance in developmental event timing is greatest at low biological levels: implications for heterochrony. Biol J Linn Soc. 2013; 110: 581-590.
8. Tills O, Rundle SD, Spicer JI. Parent-offspring similarity in the timing of developmental events: an origin of heterochrony? Proc Roy Soc B: Biol Sci. 2013; 280: 20131479-20131479.

9. Tills O, Truebano M, Rundle SD. An embryonic transcriptome of the pulmonate snail *Radix balthica*. Mar Genom. 2015; 24: 259-260.
10. Tills O, Truebano M, Feldmeyer B, Pfenninger M, Morgenroth H, Schell T, et al. Transcriptomic responses to predator kairomones in embryos of the aquatic snail Radix balthica. Ecol Evol. 2018; 31: 79-12.
