# Supplementary material for: A high-throughput and open-source platform for embryo phenomics
Source: PLoS Biol. 2018 Dec 13;16(12):e3000074. doi: 10.1371/journal.pbio.3000074 (PMC6292576; doi:10.1371/journal.pbio.3000074)
Supplement: S1 Text — OpenVIM, Open-source Video Microscope. (PDF) [file pbio.3000074.s007.pdf]

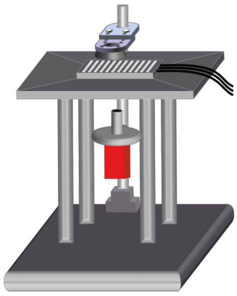

OpenVIM
Open-source-video microscope

Assembling an OpenVIM

An OpenVIM can use a range of different components, but the assembly once these are chosen and the relevant custom parts are machined is quite straight forward.

Place base in a suitable location

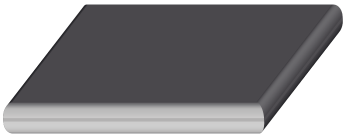

i) : For efficient recording, as with any microscopy, the OpenVIM needs to be isolated from vibration. A number of suggestions are made components of ways we have found of acheiving this, but consider citing the OpenVIM where laboratory users and air flow are minimised to help address the problem.

Attach legs to support the XY stage

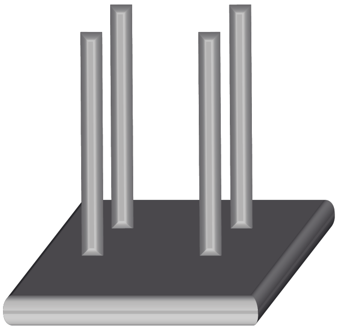

ii) : Feed machine bolts from below the pre-drilled holes in the aluminium base to the tapped holes in each aluminium leg and tighten

Attach aluminium optic support block

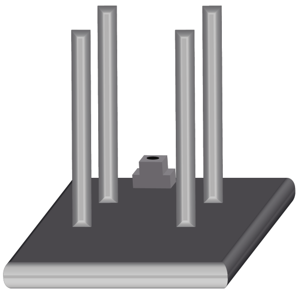

iii) : Feed machine bolts downwards through the optic support block and into the threaded holes in the alumnium base

Insert stainless steel support pole for the optics

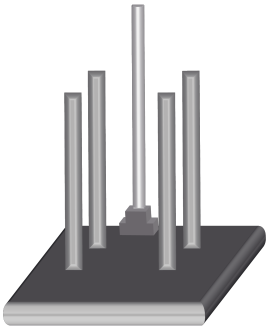

iv) : Insert the stainless steel support pole for the optics into the aluminium optic support block and tighten the grub screws to secure.

Attach motorised stage to aluminium support legs

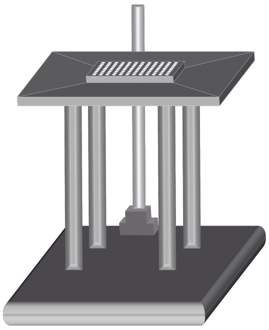

v) : Use machine head bolts to secure the motorised stage to the alumnium support legs, taking care to not exert any mechanical force over the stage. If holes fail to align, try loosening the bolts securing the legs to the base to allow the centers to locate correctly. Once the stage is fixed, fit the incubation chamber into the manufacturer provided mount atop the stage.

Attach the lens mount to the stainless steel optic support pole

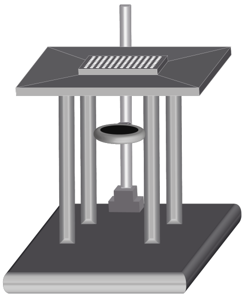

vi) : Slide the lens holder and mount down the stainless steel optic support pole.

Insert the lens into the support mount and attach camera

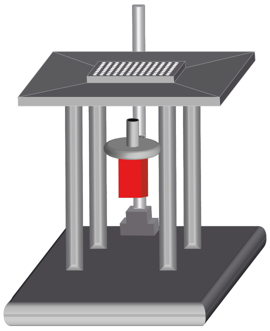

vii) : Insert the lens into the support mount inverted and tighten. Then attach the lens to the camera in an inverted orientation.

Attach lighting mount to the stainless steel optic support pole and attach LED ringlight

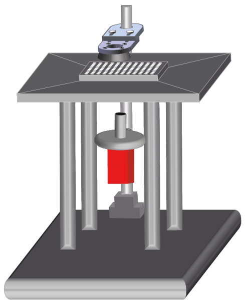

viii) : Slide the lighting mount down the stainless steel optic support pole and screw the LED ringlight to the support. Note holes will be determined by the LED, but manufacturers supply the drawings for relative mounting hole locations.

Connect motorised stage and camera to computer and launch MicroManager.

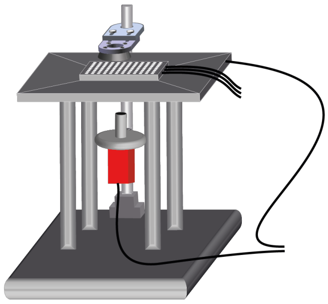

ix) : Connect the camera and motorised stage to MicroManager and initiate the 'Hardware Configuration Wizard'. This will allow you to discover the hardware and control settings. MicroManager has a good user forum and details on device support and appropriate settings can be found on the MicroManager Devices page.
